# Diabetes risk and provision of diabetes prevention activities in 44 low-income and middle-income countries: a cross-sectional analysis of nationally representative, individual-level survey data

**DOI:** 10.1016/S2214-109X(23)00348-0

**Published:** 2023-10

**Authors:** Nicholas Errol Rahim, David Flood, Maja E Marcus, Michaela Theilmann, Taing N Aung, Kokou Agoudavi, Krishna Kumar Aryal, Silver Bahendeka, Brice Bicaba, Pascal Bovet, Alpha Oumar Diallo, Farshad Farzadfar, David Guwatudde, Corine Houehanou, Dismand Houinato, Nahla Hwalla, Jutta Jorgensen, Gibson Bernard Kagaruki, Mary Mayige, Roy Wong-McClure, Bagher Larijani, Sahar Saeedi Moghaddam, Omar Mwalim, Kibachio Joseph Mwangi, Sudipa Sarkar, Abla M Sibai, Lela Sturua, Chea Wesseh, Pascal Geldsetzer, Rifat Atun, Sebastian Vollmer, Till Bärnighausen, Justine Davies, Mohammed K Ali, Jacqueline A Seiglie, Jennifer Manne-Goehler

**Affiliations:** Medical Practice Evaluation Center, Massachusetts General Hospital, Harvard Medical School, Boston, MA, USA; Department of Medicine, University of Michigan, Ann Arbor, MI, USA; Division of Infectious Diseases, Brigham and Women’s Hospital, Harvard Medical School, Boston, MA, USA; Heidelberg Institute of Global Health, Faculty of Medicine and University Hospital, Heidelberg University, Heidelberg, Germany; Behavioral Science for Disease Prevention and Health Care, Department of Sport and Health Sciences, Technical University of Munich, Munich, Germany; Medical Practice Evaluation Center, Massachusetts General Hospital, Harvard Medical School, Boston, MA, USA; Togo Ministry of Health, Lome, Togo; Bergen Centre for Ethics and Priority Setting, Department of Global Public Health and Primary Care, University of Bergen, Bergen, Norway; Diabetes and Endocrinology, Saint Francis Hospital Nsambya, Kampala, Uganda; National Institute of Public Health, Ouagadougou, Burkina Faso; University Center for General Medicine and Public Health (Unisanté), Lausanne, Switzerland; Ministry of Health, Victoria, Seychelles; Department of Epidemiology, Gillings School of Global Public Health, University of North Carolina at Chapel Hill, Chapel Hill, NC, USA; Non-Communicable Diseases Research Center, Endocrinology and Metabolism Population Sciences Institute, Tehran University of Medical Sciences, Tehran, Iran; Department of Epidemiology and Biostatistics, School of Public Health, Makerere University, Kampala, Uganda; Laboratory of Epidemiology of Chronic and Neurological Diseases, Faculty of Health Sciences, University of Abomey-Calavi, Cotonou, Benin; Laboratory of Epidemiology of Chronic and Neurological Diseases, Faculty of Health Sciences, University of Abomey-Calavi, Cotonou, Benin; Faculty of Agricultural and Food Sciences, American University of Beirut, Beirut, Lebanon; Institute of Global Health, Department of Public Health and Epidemiology, Copenhagen University, Copenhagen, Denmark; National Institute for Medical Research, Dar es Salaam, Tanzania; National Institute for Medical Research, Dar es Salaam, Tanzania; Costa Rican Social Security Fund, San José, Costa Rica; Endocrinology and Metabolism Research Center, Endocrinology and Metabolism Clinical Sciences Institute, Tehran University of Medical Sciences, Tehran, Iran; Endocrinology and Metabolism Research Center, Endocrinology and Metabolism Clinical Sciences Institute, Tehran University of Medical Sciences, Tehran, Iran; Kiel Institute for the World Economy, Kiel, Germany; Ministry of Health, Zanzibar City, Tanzania; Division of Non-Communicable Diseases, Ministry of Health, Nairobi, Kenya; World Health Organization Country Office, Pretoria, South Africa; Division of Endocrinology, Diabetes, and Metabolism, John Hopkins University, Baltimore, MD, USA; Department of Epidemiology and Population Health, Faculty of Health Sciences, American University of Beirut, Beirut, Lebanon; Non-Communicable Disease Department, National Center for Disease Control and Public Health, Tbilisi, Georgia; Ministry of Health, Monrovia, Liberia; Division of Primary Care and Population Health, Department of Medicine, Stanford University, Stanford, CA, USA; Chan Zuckerberg Biohub—San Francisco, San Francisco, CA, USA; Department of Global Health and Population, Harvard T H Chan School of Public Health, Harvard University, Boston, MA, USA; Department of Global Health and Social Medicine, Harvard Medical School, Harvard University, Boston, MA, USA; Department of Global Health and Social Medicine, Harvard Medical School, Harvard University, Boston, MA, USA; Department of Economics and Centre for Modern Indian Studies, University of Göttingen, Göttingen, Germany; Heidelberg Institute of Global Health, Faculty of Medicine and University Hospital, Heidelberg University, Heidelberg, Germany; Department of Global Health and Social Medicine, Harvard Medical School, Harvard University, Boston, MA, USA; Africa Health Research Institute, Somkhele, South Africa; MRC/Wits Rural Public Health and Health Transitions Research Unit, School of Public Health, University of Witwatersrand, Johannesburg, South Africa; Institute of Applied Health Research, University of Birmingham, Birmingham, UK; Centre for Global Surgery, Department of Global Health, Stellenbosch University, Cape Town, South Africa; Hubert Department of Global Health, Rollins School of Public Health, Emory University, Atlanta, GA, USA; Department of Family and Prevention Medicine, School of Medicine, Emory University, Atlanta, GA, USA; Diabetes Unit, Massachusetts General Hospital, Boston, MA, USA; Department of Medicine, Harvard Medical School, Boston, MA, USA; Division of Infectious Diseases, Brigham and Women’s Hospital, Harvard Medical School, Boston, MA, USA; Medical Practice Evaluation Center, Massachusetts General Hospital, Harvard Medical School, Boston, MA, USA

## Abstract

**Background:**

The global burden of diabetes is rising rapidly, yet there is little evidence on individual-level diabetes prevention activities undertaken by health systems in low-income and middle-income countries (LMICs). Here we describe the population at high risk of developing diabetes, estimate diabetes prevention activities, and explore sociodemographic variation in these activities across LMICs.

**Methods:**

We performed a pooled, cross-sectional analysis of individual-level data from nationally representative, population-based surveys conducted in 44 LMICs between October, 2009, and May, 2019. Our sample included all participants older than 25 years who did not have diabetes and were not pregnant. We defined the population at high risk of diabetes on the basis of either the presence of impaired fasting glucose (or prediabetes in countries with a haemoglobin A_1c_ available) or overweight or obesity, consistent with the WHO Package of Essential Noncommunicable Disease Guidelines for type 2 diabetes management. We estimated the proportion of survey participants that were at high risk of developing diabetes based on this definition. We also estimated the proportion of the population at high risk that reported each of four fundamental diabetes prevention activities: physical activity counselling, weight loss counselling, dietary counselling, and blood glucose screening, overall and stratified by World Bank income group. Finally, we used multivariable Poisson regression models to evaluate associations between sociodemographic characteristics and these activities.

**Findings:**

The final pooled sample included 145 739 adults (86 269 [59·2%] of whom were female and 59 468 [40·4%] of whom were male) across 44 LMICs, of whom 59 308 (40·6% [95% CI 38·5–42·8]) were considered at high risk of diabetes (20·6% [19·8–21·5] in low-income countries, 38·0% [37·2–38·9] in lower-middle-income countries, and 57·5% [54·3–60·6] in upper-middle-income countries). Overall, the reach of diabetes prevention activities was low at 40·0% (38·6–41·4) for physical activity counselling, 37·1% (35·9–38·4) for weight loss counselling, 42·7% (41·6–43·7) for dietary counselling, and 37·1% (34·7–39·6) for blood glucose screening. Diabetes prevention varied widely by national-level wealth: 68·1% (64·6–71·4) of people at high risk of diabetes in low-income countries reported none of these activities, whereas 49·0% (47·4–50·7) at high risk in upper-middle-income countries reported at least three activities. Educational attainment was associated with diabetes prevention, with estimated increases in the predicted probability of receipt ranging between 6·5 (3·6–9·4) percentage points for dietary fruit and vegetable counselling and 21·3 (19·5–23·2) percentage points for blood glucose screening, among people with some secondary schooling compared with people with no formal education.

**Interpretation:**

A large proportion of individuals across LMICs are at high risk of diabetes but less than half reported receiving fundamental prevention activities overall, with the lowest receipt of these activities among people in low-income countries and with no formal education. These findings offer foundational evidence to inform future global targets for diabetes prevention and to strengthen policies and programmes to prevent continued increases in diabetes worldwide.

**Funding:**

Harvard T H Chan School of Public Health McLennan Fund: Dean’s Challenge Grant Program and the EU’s Research and Innovation programme Horizon 2020.

## Introduction

Diabetes is one of the fastest growing chronic diseases globally and a leading cause of premature mortality, disability, and health-system costs.^1,2^ As of 2021, an estimated 537 million adults (aged 20–79 years) had diabetes worldwide and 80% of these adults lived in low-income and middle-income countries (LMICs).^3^ The steep rise in diabetes prevalence over the past two decades^4^ and its pivotal role in delaying progress toward the Sustainable Development Goals^5^ led WHO to launch the Global Diabetes Compact in April, 2021.^6^ One of the goals of the Global Diabetes Compact is to provide a comprehensive and inclusive plan to support countries in implementing effective programmes for the prevention of diabetes in adults.^6,7^ However, an important obstacle to the implementation of prevention metrics into the Compact’s global targets^7^ has been the dearth of data on diabetes prevention activities for people at risk of diabetes across settings.

The use of non-pharmacological approaches to reduce the incidence of diabetes among individuals at high risk of this condition has been a topic of longstanding research inquiry.^8–13^ Multilevel and multicomponent interventions for diabetes prevention that target the individual have been proposed and studied in a wide variety of settings.^14,15^ First, three seminal studies of individual-level diabetes prevention were conducted in China,^11^ Finland,^12^ and the USA^8^ two decades ago. These studies showed that the incidence of diabetes could be reduced by 42–58% among adults with impaired glucose tolerance and overweight or obesity who participated in non-pharmacological lifestyle interventions, such as diet modification, bodyweight reduction, and increased physical activity.^8,11,12^ More modest reductions in diabetes risk were subsequently documented with non-pharmacological interventions implemented in real-world settings.^16,17^ Although challenges with cost of, access to, and participant engagement with these programmes have been reported, diabetes prevention at the individual-level remains an important, evidence-based component of comprehensive strategies to halt this growing epidemic.^18–20^

Given that diabetes prevention is also an important dimension of the Global Diabetes Compact,^6,7^ evaluating the state of individual-level diabetes prevention activities in LMICs can establish unmet needs for these services and inform future prevention metrics, as well as policies and programmes to prevent diabetes. In this study, we aimed to describe the proportion of the population at high risk of developing diabetes; quantify individual-level prevention activities among the population at high risk of developing diabetes across a large, heterogeneous group of LMICs; and explore variation in reported prevention activities for diabetes by country-level and individual-level characteristics.

## Methods

### Study design

In this cross-sectional observational study, we performed a pooled analysis of participant-level data from 45 nationally representative, population-based surveys in 44 LMICs (Zanzibar and mainland Tanzania each conducted an independent survey, but both are part of Tanzania) from the Global Health and Population Project on Access to Care for Cardiometabolic diseases (HPACC).^21^ These observational health surveys, conducted during October, 2009, and May, 2019, measured biomarkers of cardiovascular disease risk factors, including for diabetes, and assessed health services for these conditions. The search and harmonisation methods have been previously published and key details are also included in the appendix (pp 3–4). Countries were categorised into World Bank income groups^22^ and into six geographical regions, adapted from the NCD Risk Factor Collaboration geographical classification (appendix p 6).^4^ Country-specific sampling methods for these surveys are provided in the appendix (pp 7–26). The requirements for inclusion of a country survey from the parent dataset in this study were that the survey was conducted in 2009 or after; had data available at the individual level; was conducted in a low-income, lower-middle-income, or upper-middle-income country, according to the World Bank income groups classifications in the year that the survey was conducted;^22^ was nationally representative, such that the sampling approach ensures representation of the underlying national population by sex and age; had at least 60% participation in diabetes biomarker collection (either a blood glucose or haemoglobin A_1c_ [HbA_1c_] measurement); and contained at least one question concerning a diabetes prevention activity of interest, without conditioning on a previous diagnosis of diabetes or any other health condition.

Ethics approval for the data in this study was obtained in-country at the time of data collection by each country’s respective surveying team. Standard ethics procedures were followed, which included asking for participants’ informed consent to participate in the respective survey. Surveys were collated into the final analytical dataset by the Global Health and Population Project on Access to Care for Cardiometabolic diseases. The resulting dataset was designated as Non-Human Subjects Research by the Harvard T H Chan School of Public Health in 2018 under protocol IRB16–1915.

The reporting of this manuscript follows the STROBE guidelines (appendix pp 74–76).

### Participants

Our study sample included participants aged 25 years and older, given that 25 years was the minimum age for inclusion in most surveys in this analysis (appendix pp 7–26). We further restricted our analysis to participants who were not pregnant, who had a diabetes biomarker collected during the survey, and who did not meet biochemical criteria for diabetes as defined by a fasting plasma glucose of 7·0 mmol/L or more,^23,24^ a HbA_1c_ of 6·5% or higher,^23,24^ or self-reported use of glucose-lowering medication.

### Procedures

Among the individuals in the study sample, we defined the population at high risk of developing diabetes (and thus eligible for diabetes prevention activities) on the basis of recommendations in the WHO Package of Essential Noncommunicable (PEN) Disease Interventions for Primary Health Care.^25^ These WHO PEN recommendations are generally consistent with eligibility criteria for randomised controlled trials that aim to test the effectiveness of diabetes prevention interventions.^8,11,12^ One important difference between the data available in the surveys and both the WHO criteria to diagnose impaired glucose tolerance and randomised-controlled-trial eligibility criteria to participate in diabetes prevention trials is the absence of oral glucose tolerance tests in these surveys that, if present, could be used to diagnose impaired glucose tolerance^23^—another common criterion for identifying populations at high risk of diabetes. In this study, we defined participants as being at high risk of developing diabetes if they had any of the following: fasting plasma glucose between 6·1 and 6·9 mmol/L (110–125 mg/dL) based on WHO criteria for impaired fasting glucose;^26^ HbA_1c_ between 5·7% and 6·4% based on the American Diabetes Association criteria for prediabetes^23^ (WHO does not use HbA_1c_ in its definition of impaired fasting glucose);^26^ presence of obesity (with a threshold BMI of at least 30 kg/m²) among individuals younger than 40 years; or presence of overweight (with a threshold BMI of at least 25 kg/m²) among individuals aged 40 years or older.^25^ For the east, south, and southeast Asia region, we used a BMI threshold of at least 23 kg/m² among individuals aged 40 years or older, on the basis of WHO guidelines for the appropriate BMI cutoffs to diagnose overweight in Asian populations.^27^

We used point-of-care fasting capillary glucose (appendix p 27) in 39 of the 45 included surveys in which it was the only diabetes biomarker collected. Plasma equivalents were provided by all but five of these surveys. For these five surveys, we multiplied capillary glucose values by 1·11 so that all values used in our study would be plasma equivalents. This adjustment is standard in population-based diabetes analyses that use pooled data and is based on evidence that capillary glucose often underestimates plasma glucose concentrations.^28,29^ For 11 surveys that did not specify the device used to measure capillary glucose, we assumed no correction was required as plasma glucose concentration was the most common form of reporting across surveys (ie, no plasma equivalent was computed). In four of the 45 surveys (Bangladesh, Costa Rica, Iraq, and Lebanon), a laboratory-based measurement of fasting plasma glucose was the only diabetes biomarker used. In two surveys (Iran and Seychelles) in which both HbA_1c_ and fasting glucose were available, we used HbA_1c_ as the biomarker of interest. All surveys required a minimum of 8 h of fasting, which was defined as no food or drink intake other than water. Details on fasting instructions for each survey are provided in the appendix (p 28).

In all surveys, height was measured in the standing position with a portable height-measuring board, such as those from seca or Shorr Productions.^30,31^ Weight was measured with a portable weighing scale, such as the seca scale or Tanita HS301 Solar Scale.^30,31^ Participant BMI values were then calculated by dividing the individual’s weight (measured in kg) by height (measured in metres) squared.

Our metrics of interest were four diabetes prevention activities recommended in the WHO PEN.^25^ We defined these activities on the basis of respondents’ self-report of having received the following from a health-care professional: counselling to increase physical activity, counselling to reduce bodyweight, counselling to increase dietary fruit and vegetable intake, and blood glucose testing (ie, screening). The first three metrics were consistent with key lifestyle interventions shown to be effective in preventing diabetes among high-risk populations. Although not necessarily delivered in the context of a comprehensive diabetes prevention programme, these interventions are all well accepted, fundamental prevention activities.^8,11–13,17^ These survey questions were not conditioned on having a previously diagnosed health condition, including hypertension or diabetes, and an overview of the survey wording used to ask whether a participant had been counselled is provided in the appendix (p 35). Although the long-term benefits of diabetes screening have not been clearly shown in randomised trials,^32^ blood glucose testing among subgroups at high risk is widely recommended in guidelines—not only guidelines from the WHO PEN^25^ but also those from the International Diabetes Federation,^33^ the American Diabetes Association,^23^ and the US Prevention Services Task Force.^34^ This screening is also a crucial step in identifying people with prediabetes. Finally, increasing diagnosis of diabetes is also a key metric for WHO’s Global Diabetes Compact Targets.^7^

We explored variation in diabetes prevention activities based on the following individual-level sociodemographic characteristics: age (classified into the categories 25–34 years, 35–44 years, 45–54 years, 55–64 years, and ≥65 years); sex (all sex or gender data were self-reported by participants; the options for sex in the STEPwise Approach to Non-communicable Disease Risk Factor Surveillance [ie, in Tanzania and Zanzibar] and in Demographic and Health Surveys were male or female and the options for gender in the Chilean National Health Survey survey were woman or man); educational attainment (in the categories of no formal schooling, less than secondary schooling, and some secondary schooling or higher); and rural versus urban setting. In addition, in sensitivity analyses, we also considered variation by household wealth quintile. Details on the construction and harmonisation of wealth quintiles are provided in the appendix (p 29). Educational attainment was the preferred indicator of socioeconomic status on the basis of previous work that suggests education level could be a more sensitive and stable marker of socioeconomic status than household wealth quintile when diabetes is the exposure variable.^35^

### Statistical analysis

We conducted analyses focusing on both the country and individual levels. In the country-focused analysis, we estimated the proportion of respondents in each survey at high risk of developing diabetes. Next, we calculated the proportion of the population at high risk that had reported each of the four diabetes prevention activities. This calculation was performed in the overall sample and by country categories, stratified by World Bank income groups. Then, among the population at high risk, we assessed the total number of diabetes prevention activities that each respondent reported (ie, zero to four activities); this analysis was performed on a subsample of countries (n=29) in which data were available for all four metrics of interest.

In the individual-level analysis, we constructed separate univariable and multivariable Poisson regression models examining associations between individual-level sociodemographic characteristics and each outcome (appendix pp 49–56). To facilitate model interpretation, we reported regression output both as risk ratios and the absolute difference in predicted probabilities with average marginal effects.^36^ All descriptive statistics and regression models were estimated with a robust error structure, with standard errors adjusted for clustering at the primary sampling unit through Stata’s suite of survey commands that calculate standard errors with the linearised variance estimator. We used sampling weights to adjust for the probability of selection, non-response, and differences between the sample population and the target population. Moreover, in pooled analyses, we rescaled the survey weights such that each country was weighted equally. We performed this rescaling because the health system was the primary unit of interest in this analysis; thus, each survey contributes equally and no single survey from a highly populous country dominates the pooled results. All regression models accounted for country-level fixed effects. Data were analysed with Stata version 16.1.

We conducted several robustness checks to test the validity of our results. First, as noted above, we included household wealth quintile in the multivariable Poisson models for the subset of countries with available data (n=36 surveys). Second, we re-estimated the multivariable models on the subset of the population that was defined as high risk of developing diabetes only on the basis of the BMI criteria outlined previously. Finally, we redefined individuals at high risk of developing diabetes with an adaptation of the Finnish Diabetes Risk Score.^37^ With the available data, we were able to include all elements of the score except for family history and amount of physical activity. We then calculated the proportion of the sample that had scores of 9 or higher, as a rescaled threshold based on the lower theoretical maximum score of 19 (as compared with the original maximum score of 26) for being considered at high risk of diabetes (detailed explanation provided in the appendix; pp 57–58). We then re-estimated the proportion of participants considered at high risk who reported receiving each of the preventive activities. The detailed results of all sensitivity analyses are provided in the appendix (pp 59–70).

### Role of the funding source

The funders of the study had no role in study design, data collection, data analysis, data interpretation, or writing of the report.

## Results

The survey characteristics are summarised in the appendix (pp 36–37). The final study sample included 145 739 individuals in 44 LMICs across 45 surveys conducted between October, 2009, and May, 2019. A flow diagram of participant inclusion is provided in the appendix (p 5). 86 269 (59·2%) of 145 739 participants were female and 59 468 (40·4%) were male. The mean response rate across surveys was 88·7% (SD 11·8).

Among our sample of participants older than 25 years who were not pregnant and did not have diabetes, a total of 59 308 (40·7% [95% CI 38·5–42·8]) individuals were considered at high risk of developing diabetes. Among the individuals considered at high risk, 5895 (9·9% [8·0–12·3]) were at high risk due to presence of prediabetes (defined with HbA_1c_) or impaired fasting glucose, 45 517 (weighted percentage 78·4% [95% CI 74·5–81·8]) were at high risk based on BMI criteria, and 7896 (weighted percentage 11·8% [10·1–13·6]) were at high risk due to both prediabetes or impaired fasting glucose and BMI criteria. The age category with the highest weighted percentage in the total sample was the group aged 25–34 years (35·3%), whereas the age category with the highest weighted percentage among individuals considered at high risk of diabetes was the group aged 45–54 years (28·8%). Both in the total sample and population at high risk, the highest weighted percentage of individuals had at least some secondary education. Individual-level characteristics are fully described in the table.

**Figure 1:**
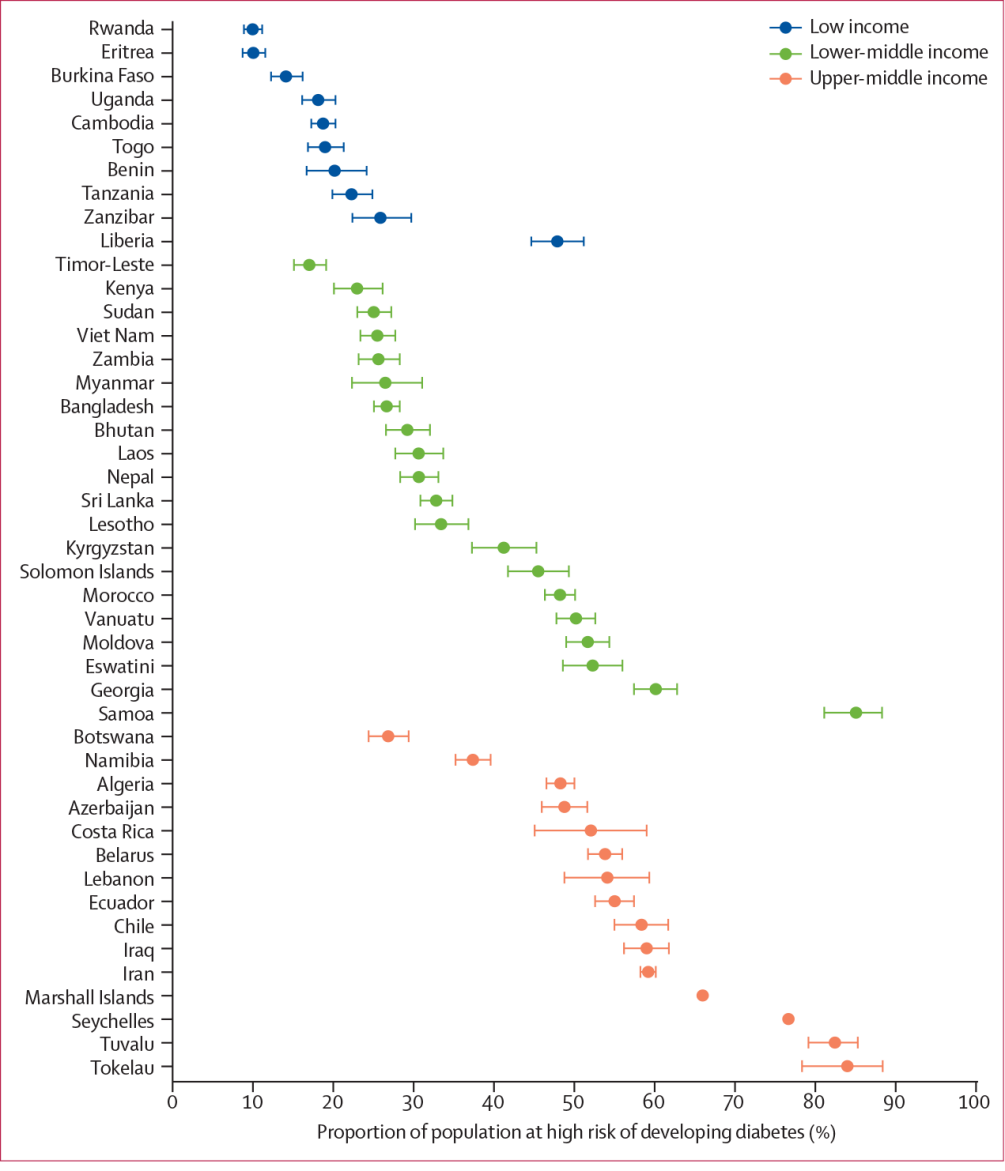
Proportion of the population at high risk of developing diabetes by country Figure shows the weighted estimated proportion of each country’s population deemed at high risk of developing diabetes based on the WHO Package of Essential Noncommunicable Disease Guidelines. Division of income is according to World Bank income group. Sample excludes those who meet criteria for a diagnosis of diabetes or are pregnant. Error bars represent 95% CIs.

The proportion of each country’s overall sample that was considered at high risk of diabetes is illustrated in figure 1 and provided in the appendix (pp 39–43). When stratified by World Bank income groups, this proportion was 20·6% (95% CI 19·8–21·5) for low-income countries, 38·0% (37·2–38·9) for lower-middle-income countries, and 57·5% (54·3–0·6) for upper-middle-income countries. Generally, the proportion of the population at high risk increased with World Bank income groups and ranged from 10·0% (95% CI 8·9–11·2 [Rwanda] and 8·71–1·5 [Eritrea]) to 47·9% (44·7–51·2 [Liberia]) for low-income countries, 17·0% (15·1–19·1 [Timor-Leste]) to 85·1% (81·1–88·3 [Samoa]) for lower-middle income countries, and 26·8% (24·4–29·4 [Botswana]) to 84·0% (78·4–88·4 [Tokelau]) for upper-middle income countries.

**Figure 2:**
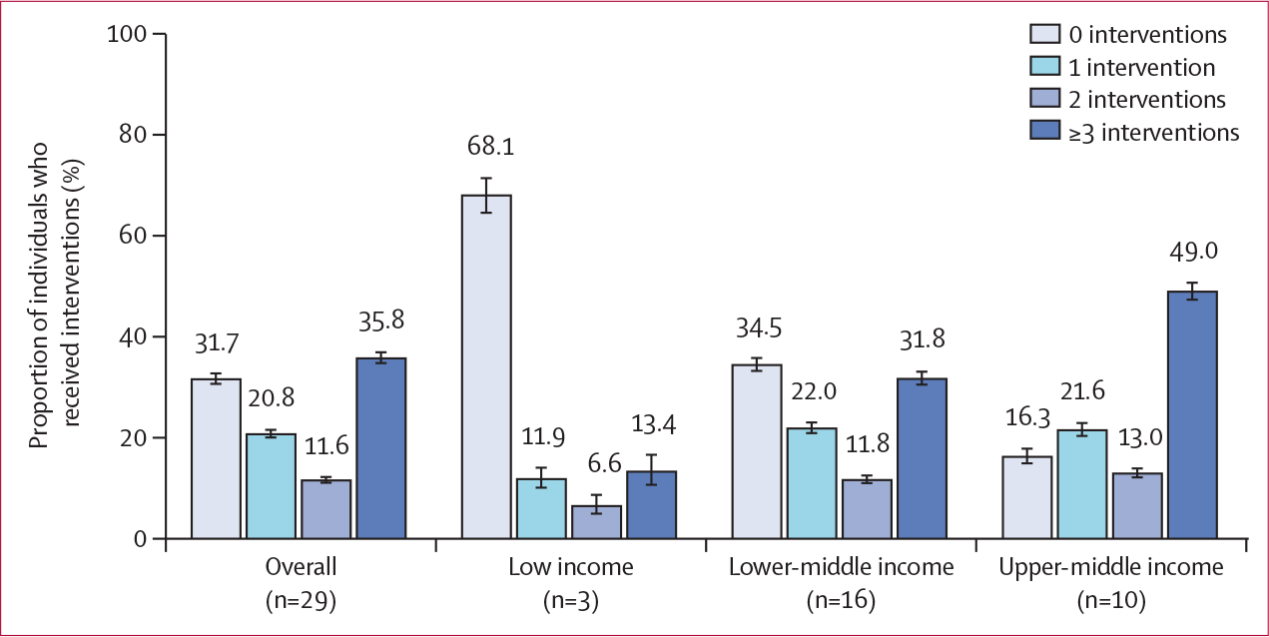
Self-reported number of diabetes preventive services received Number of countries in each World Bank income group listed below each label. Estimates of prevalence were calculated with sampling weights readjusted to weigh each country equally. Error bars represent 95% CIs.

Figure 2 shows the number of diabetes prevention activities reported, overall and by World Bank income groups. Overall, 31·7% (95% CI 30·7–32·7) of 38 879 respondents in this analysis reported no prevention activities, 20·8% (20·1–21·6) reported one prevention activity, 11·6% (11·1–12·2) reported two prevention activities, and 35·8% (34·8–36·9) reported three or more prevention activities. When stratified by World Bank income groups, the largest segment of the population at high risk in low-income and lower-middle-income countries reported no prevention activities (68·1% [64·6–71·4] in low-income countries and 34·5% [33·2–35·8] in lower-middle-income countries), whereas the largest segment of the population at high risk in upper-middle-income countries reported three or more prevention activities (49·0%; [47·4–50·7]).

**Figure 3:**
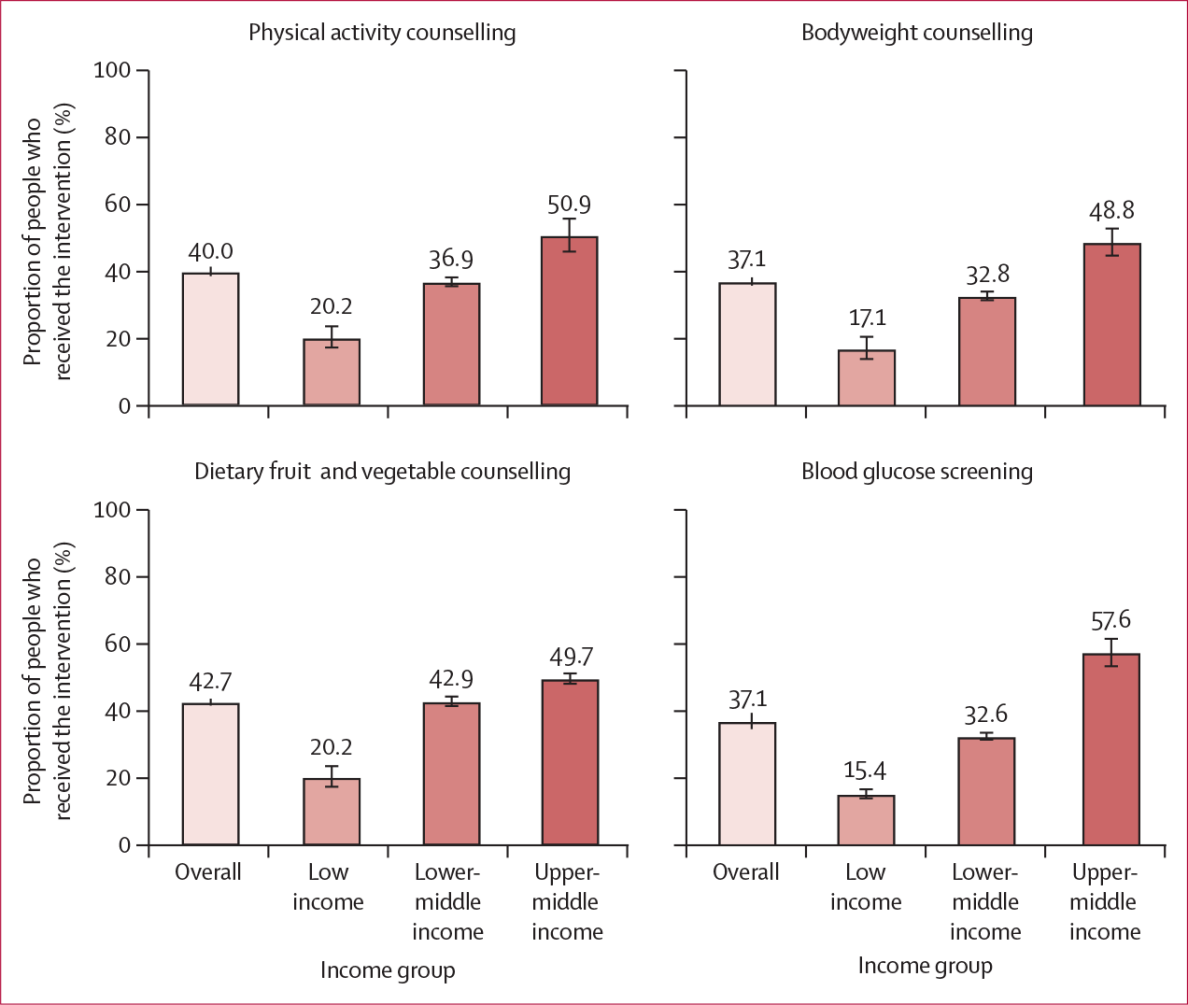
Proportion of the population at high risk who self-reported having received each diabetes preventive intervention by World Bank income group Sampling weights were adjusted to represent each country equally. Error bars represent 95% CIs.

Figure 3 illustrates the proportion of the population at high risk that reported each of the prevention activities of interest, categorised by World Bank income groups (appendix pp 44–48). There were similar rates of attainment across metrics, with 40·0% (38·6–41·4) counselled to increase physical activity, 37·1% (35·9–38·4) counselled to reduce bodyweight, 42·7% (41·6–43·7) counselled to increase dietary fruit and vegetable intake, and 37·1% (34·7–39·6) screened via blood glucose. When stratified by World Bank income groups, the lowest attainment rate for all four activities was observed in low-income countries and ranged from 15·4% (14·1–16·8) for blood glucose screening to 20·2% (17·3–23·5) for physical activity and dietary fruit and vegetable counselling. In contrast, the proportion of people who received a prevention service in lower-middle-income countries ranged from 32·6% (31·6–33·7) for blood glucose screening to 42·9% (41·5–44·3) for dietary fruit and vegetable counselling. Upper-middle-income countries had the greatest attainment for all four activities, ranging from 49·7% (48·2–51·2) for dietary fruit and vegetable counselling to 57·6% (53·5–61·7) for blood glucose screening.

**Figure 4:**
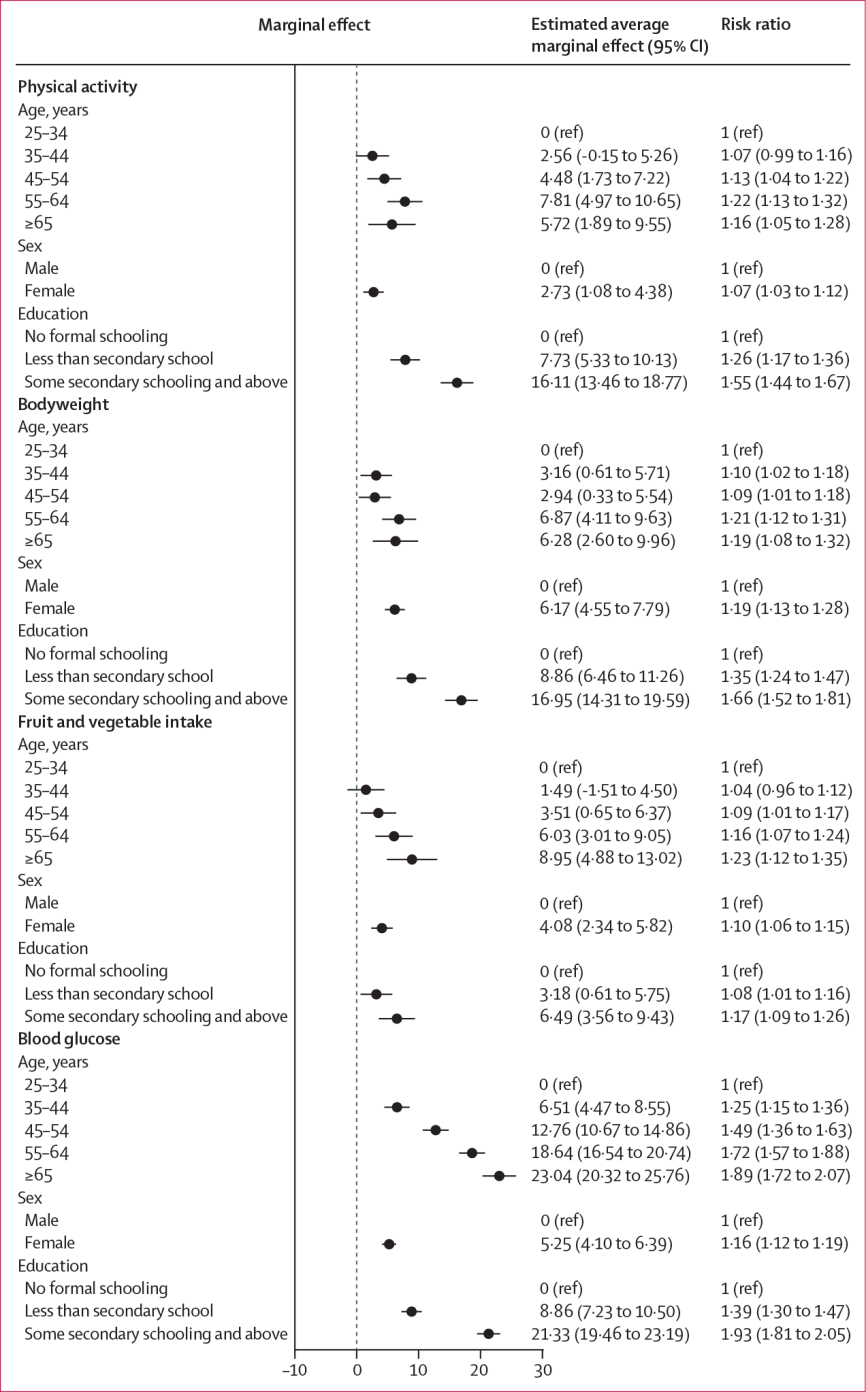
Association between individual characteristics and having received diabetes preventive services The marginal differences and 95% CIs in predicted probability of reporting having received each preventive service are depicted, with the base category for each sociodemographic characteristic serving as the reference point. Units for the estimated average marginal effect are percentage point differences in predicted probability. Error bars represent 95% CIs. Models were estimated with sampling weights readjusted to represent each country equally, and accounted for country-level fixed effects.

Results from univariable Poisson regression models are presented in the appendix (pp 49–52). In multivariable models, we observed that some secondary education or above was associated with both counselling to increase physical activity and to reduce bodyweight, with differences in predicted probabilities of 16·1 (95% CI 13·5–18·8) percentage points for physical activity counselling and 17·0 (14·3–19·6) percentage points for bodyweight counselling, compared with having no formal schooling (figure 4; appendix pp 53–56). Being aged 65 years and older was the characteristic most strongly associated with counselling to increase dietary fruit and vegetable intake and with blood glucose screening, with marginal effects of 8·9 (4·9–13·0) and 23·0 (20·3–25·8) percentage points, respectively, compared with being aged 25–34 years. Being female and having secondary educational attainment or more had consistent, positive associations with all four prevention activities.

Sensitivity analyses, including wealth, area of residence (ie, rural or urban), and those in which the models were re-estimated on the subsample that was considered high risk on the basis of BMI criteria only (ie, physical activity n=35 889, bodyweight n=37 012, diet n=34 878, and blood glucose n=51 498), did not substantially alter the results (appendix pp 59–70). When compared with the proportion of the sample considered high risk of developing diabetes through our primary criteria, the sensitivity analysis with the adapted Finnish Diabetes Risk Score yielded a smaller population at high risk (21 694 [weighted percentage 16·0%; 95% CI 15·4–16·6] of 125 448). However, trends in reported prevention activities by World Bank income groups were consistent between analyses with the Finnish Diabetes Risk Score and our original criteria (appendix pp 71–73).

## Discussion

In this study of 145 739 adults aged 25 years or older across 44 LMICs, we found that approximately two in every five individuals were considered at high risk of developing diabetes as defined by impaired fasting glucose or presence of overweight or obesity. Overall, less than half of people who were deemed to be at high risk of developing diabetes reported having received any one of the four key diabetes-prevention activities of interest in this study that are recommended in current international guidelines.^25^ Additionally, there were large disparities in individual-level prevention activities reported by national-level wealth: approximately two in three people at high risk of diabetes in low-income countries reported no prevention activities, whereas about half of people at high risk in upper-middle-income countries reported at least three prevention activities (figure 2). Given that one of the key goals of the Global Diabetes Compact^6,7^ is the integration and broader reach of diabetes prevention, these findings are important to inform future global prevention targets and facilitate the development and implementation of programmes to identify and more rapidly link individuals at high risk to evidence-based programmes for diabetes prevention in these settings.

Overall, counselling on dietary fruit and vegetable intake was the most frequently reported prevention activity, followed by counselling to increase physical activity, and finally counselling to reduce bodyweight and blood glucose screening. However, when stratified by World Bank income groups, we found the greatest disparity for blood glucose screening, whereby the proportion of the population at high risk of diabetes who had been screened was more than three times greater in upper-middle-income countries (57·6%) than in low-income countries (15·4%). These disparities could be attributed to barriers at the patient level (eg, low awareness of the condition), clinician level (eg, few guidelines and little knowledge about diabetes risk assessment), and system level (eg, access to laboratory materials and services), and underscore broader challenges regarding achievement of global targets for diagnosis of individuals at high risk of diabetes in low-resource settings.^38^

We also found that the greatest proportion of individuals at high risk of diabetes lived in urban areas and in upper-middle-income countries. This finding is consistent with the nutrition and obesity transition frameworks, which posit that the increase in metabolic disease occurring in LMICs can be tracked along discrete stages defined by economic development.^39,40^ Although these patterns have been described for overweight, obesity, and diabetes previously,^35,40^ our study expands upon this literature by showing that the population considered at high risk of diabetes is greatest in LMICs at the most advanced stages of economic development and that people in upper-middle-income countries were most likely to report diabetes prevention activities. Some individual-level sociodemographic characteristics, including older age (ie, ≥65 years), being female, and having educational attainment at the level of secondary school or above were associated with a greater probability of receiving one or more prevention activities than younger age, being male, and having less than secondary schooling. Given that diabetes risk is strongly linked to an individual’s social context^41^ and is expected to shift from high to low socioeconomic status populations in LMICs with economic development,^39,40^ interventions that increase access to these prevention services across socioeconomic strata in resource-limited settings are urgently needed.

Two decades after the landmark Diabetes Prevention Program studies^8,11,12^ showed that intensive lifestyle interventions (including dietary modification and exercise) could substantially reduce the incidence of diabetes among individuals at high risk, real-world implementation of effective individual-level diabetes prevention remains a pressing challenge.^20^ Our study adds to this evidence base by also showing the very small reach of a suite of fundamental diabetes prevention activities in many LMICs. Adaptations of the Diabetes Prevention Program have been conducted in various settings,^16,17^ and although these studies were initially limited to mostly high-income contexts, a growing number of studies focused on diabetes prevention have been or are being conducted in LMICs.^42–45^ It is noteworthy that 39 of the 44 LMICs in this study have implemented health-systems programmes that formally include a diabetes prevention component (appendix pp 30–34). Many of these programmes have been led in partnership with the World Diabetes Foundation. Although the outcome of such programmes in terms of diabetes incidence reduction remains unclear, this study offers an initial assessment of the reach of the most fundamental prevention activities, including key lifestyle messages and diabetes screening efforts, across contexts. Previous research has detailed numerous barriers to the successful and sustained implementation of programmes similar to the Diabetes Prevention Program and activities at the health-systems level (ie, the cost to sustain such programmes, inadequate referral systems, and a poor testing infrastructure to identify individuals at high risk of diabetes), the health-care provider level (ie, little knowledge or belief in the effectiveness of such programmes in real-world settings), and the patient level (ie, competing priorities and a perception that the benefit might not outweigh the costs and time required to participate).^15,16,18,19^ Strategies that could be considered to strengthen and scale these programmes include the use of lay educators, which has shown similar effectiveness with reduced costs;^16^ incorporating behavioural economics principles to improve motivation and incentivise participation;^16,19,46,47^ and implementing mobile technology, which is increasing in LMICs^48^ and offers an important tool to improve user engagement and sustainability.^49,50^

In addition to the individual-level diabetes prevention activities discussed in this study, it is important to acknowledge the growing focus on population-wide policy approaches that aim to address so-called upstream risk factors for diabetes, the effectiveness of which does not rely on a sustained agentic individual response.^15,51^ For instance, over 50 countries have introduced taxes on sugar-sweetened beverages,^52^ with data from the UK,^53^ Mexico,^54,55^ and South Africa^56^ showing a reduction in the purchase or consumption of such beverages following policy implementation, even when increases in sugar intake from untaxed items increased.^56^ Although these outcomes are generally encouraging, long-term evaluation to assess the societal effect of these policies on diabetes or diabetes risk factor incidence is needed. Finally, these population-wide interventions are likely to offer an important complementary or adjunctive approach to individual-level diabetes prevention messages and programmes.^57^

Broadly, our findings have important implications for the WHO Global Diabetes Compact,^6,7^ which aims to respond to the increased burden of diabetes globally, in part by supporting efforts to prevent diabetes in regions with accelerating epidemics. Unifying stakeholders to improve the prevention of modifiable risk factors for diabetes, such as physical inactivity or unhealthy diet, and integrating diabetes prevention into primary health care are two priorities of the Compact.^6,7^ To our knowledge, our study provides the first empirical evidence about individual-level prevention activities for the large populations at highest risk of developing diabetes in these settings. Previously, the scarcity of data about these activities in LMICs has impeded the inclusion of a prevention-focused target that might motivate scale-up of these services. These findings provide an evidence-base for such a metric in the future and can be used to aid resource prioritisation and allocation, furthering the Global Diabetes Compact’s ambitious vision.^7^

Our study has several limitations. First, although we used the World Bank groupings to describe the population at high risk of diabetes and the receipt of prevention activities in LMICs, this categorisation could mask heterogeneity in health-system infrastructure and capacity. Given the complex forces driving the rising burden of diabetes in these settings, the disparities reported in this study should be interpreted within the context of each country’s epidemiology and health system. Second, the definition of high risk for diabetes by biochemical measures was limited to a single glucose measurement in some countries and was based on capillary measurement in many surveys. Although widely accepted measurements in epidemiological research, single glucose values are subject to measurement errors, including regression to the mean, that can affect population-based estimates of diabetes.^58^ Although some of these surveys have previously been criticised for incorrect interpretation of these glucose measures, we have rigorously researched the glucometers used for measurement and worked with national and international experts to ensure the interpretation of these values is accurate (appendix p 27). We also chose to make a widely used adjustment in five surveys to ensure that capillary glucose approximates plasma glucose as accurately as possible; this adjustment is supported by expert consensus and is commonly used in population-health research. Third, the prevention activities explored in this study are self-reported and thus could be subject to recall and social desirability bias since respondents might have perceived that an affirmative response was desired; this reporting also does not clearly capture whether this advice was offered through an intensive diabetes prevention programme or in a one-time manner during routine care. Fourth, there was heterogeneity across surveys in terms of which questions about prevention activities were asked; we attempted to address this by denoting specific instances in which surveys were missing items. Along with heterogeneity in survey instruments, there is also heterogeneity in the sizes of the populations being surveyed. Because the countries featured in this study have populations of varying sizes, we cannot rule out the possibility that the size of a national health system could affect its organisation and, consequently, its performance. Furthermore, because these surveys were conducted in different years, it is possible that differences in estimates of prevention activities were due, at least in part, to period effects. Related to this, because this study is cross-sectional, we are only able to gain a snapshot of data for these countries in the year of the survey but cannot track progress of their health systems or establish causal relationships. Finally, we included data from 44 LMICs and both prevalence estimates, and the relationships observed in this cohort might not apply to LMICs not included in this study.

Although the original diabetes prevention trials^8,11,12^ were conducted among individuals with impaired glucose tolerance (defined on the basis of oral glucose tolerance test data), oral glucose tolerance tests are not generally performed in population-level surveys in LMICs. Thus, the definition of the population at risk of diabetes was developed on the basis of the best currently available, individual-level biomarker data in LMICs and the most widely accepted global guidelines. To further address potential limitations in the chosen approach to defining the population at high risk of diabetes, we conducted a sensitivity analysis in which we used an adapted Finnish Diabetes Risk Score as an alternative approach to define the population at high risk of diabetes. The results of this analysis identified a substantially smaller population deemed at high risk of diabetes, modestly higher absolute estimates of prevention activities received overall, and a nearly identical pattern of disparities in prevention activities across World Bank income groups than the original analysis. This sensitivity analysis thus offers an informative set of alternative estimates to those in our main analysis and reaffirms the national wealth trends identified in the main analysis but does so with criteria that have been widely accepted in epidemiological research on diabetes. That being said, it is important to highlight that the accuracy of estimates based on the Finnish Diabetes Risk Score are uncertain because this scoring approach has not been validated in most LMICs included in our dataset and we had to adapt the overall score due to the absence of two variables that are typically included but were unavailable to us.^59,60^

In conclusion, diabetes is a major global problem, and there are large and growing populations of adults at high risk of progression to diabetes. This study highlights substantial populations of people at high risk of diabetes, the large unmet need for individual diabetes prevention activities globally, and staggering disparities in these prevention activities across countries at different levels of wealth. Future research should evaluate how best to mitigate these disparities and improve the implementation, scalability, and sustainability of diabetes prevention activities in LMICs.

## Supplementary Material

1

## Figures and Tables

**Table:** Individual characteristics of the overall sample and group at high risk of diabetes

	Total population (N=145 739)		Population at high risk (N=59 308)	
	Unweighted, n	Weighted, %	Unweighted, n	Weighted, %

**Age**				
25–34 years	40 599	35⋅3%	7015	19⋅3%
35–44 years	39 912	28⋅3%	14 964	28⋅1%
45–54 years	32 794	19⋅8%	18 561	28⋅3%
55–64 years	23 846	12⋅9%	13 430	18⋅6%
≥65 years	8588	3⋅6%	5338	5⋅3%
**Sex**				
Male	59 468	48⋅1%	21 420	43⋅0%
Female	86 269	51⋅9%	37 887	57⋅0%
**Education**				
No formal schooling	33 948	19⋅0%	11 181	14⋅2%
Lessthan secondary schooling	52 585	35⋅0%	19 751	32⋅5%
Some secondary schooling and above	57 574	46⋅0%	27 161	53⋅3%
**Area of residence**				
Urban	44 268	42⋅7%	22 832	52⋅0%
Rural	51 874	57⋅3%	18 145	48⋅0%
**Wealth quintile**				
Poorest	23 908	21⋅6%	8763	20⋅1%
Poorer	21 939	20⋅3%	8451	19⋅6%
Middle	20 910	20⋅6%	8492	20⋅5%
Richer	18 698	18⋅7%	7869	19⋅2%
Richest	17 785	18⋅8%	8184	20⋅6%

Proportions were calculated with weights provided by the individual surveys, readjusted such that each country is weighted equally.

## Data Availability

A majority of the surveys included in the HPACC dataset are publicly available. The two most common data sources are the WHO data repository (https://extranet.who.int/ncdsmicrodata/index.php/home) and the Demographic and Health Surveys’ website (https://dhsprogram. com/data/). Several additional surveys have been obtained through formal requests to survey teams whose data are not already made public. Information on where surveys were sourced can be found in the appendix (pp 7–26). The pooled, harmonised, de-identified participant-level HPACC dataset and accompanying data dictionary have been created through a partnership between Harvard University, University of Göttingen, and Heidelberg University, in collaboration with all country-level survey teams. Access can be requested by contacting the corresponding author. More information about HPACC, including additional contact information for the collaboration, can be found on https://www.hpaccproject.org/.

## Research in context

### Evidence before this study

We searched PubMed on March 8, 2023, without language or date restrictions with the search terms “diabetes prevention program” AND “nationally representative” AND “((reach) OR (adoption) OR (receipt) OR (utilization))” in all fields. This search yielded two studies examining diabetes prevention activities through nationally representative surveys. Both studies analysed data from the National Health Interview Survey (NHIS), which is a cross-sectional survey of US households conducted annually. The first study analysed data from the 2016 NHIS to estimate the prevalence of diabetes prevention programme referral and participation among adults probably eligible for diabetes prevention programming (with self-reported diagnosis of prediabetes or gestational diabetes and meeting body mass index criteria). Among 28 345 respondents, 2341 adults (8·1% of the study sample) were eligible for diabetes prevention activities, of whom 4·2% reported ever being referred to a 12-month diabetes prevention programme and 2·4% reported ever participating in a diabetes prevention programme. The second study analysed data from the 2016 and 2017 NHISs. From a sample of over 50 000 respondents, the authors found that 36·0% of individuals met their criteria for being at high risk of diabetes; among them, 73·5% of people who had been diagnosed with prediabetes and half of the people who were at high risk but not formally diagnosed with prediabetes reported receiving advice or referrals for diabetes risk reduction from their health-care professional. We did not find comparable studies from low-income and middle-income settings.

### Added value of this study

In this study, we analysed nationally representative data from over 145 000 individuals across 44 low-income and middle-income countries (LMICs) to evaluate diabetes prevention activities for people at high risk of diabetes. Our study adds value to the current literature in several ways. First, we use survey data from a large suite of LMICs to describe diabetes prevention activities undertaken by health systems globally, whereas the literature to date has almost exclusively centred on high-income countries. Second, BMI and glucose were measured in each of these surveys, allowing us to robustly identify the population at risk of diabetes who would be eligible for prevention according to current international guidelines. Third, we have detailed self-reported information on a range of important counselling activities and glucose screening and associated individual-level sociodemographic characteristics, allowing us to offer a nuanced assessment of variation in the receipt of diabetes prevention activities across settings.

### Implications of all the available evidence

We found large unmet needs for fundamental individual-level diabetes prevention activities across this group of countries. However, prevention activities increased steeply with rising national-level wealth, such that half of all people at high risk of diabetes reported three or more activities in upper-middle-income countries, whereas over two-thirds of individuals living in low-income countries reported none of these activities. Strengthening of prevention activities is a highly important component of efforts to halt the rise of diabetes worldwide, including through the Global Diabetes Compact.
