# Supplementary material for: Diabetes risk and provision of diabetes prevention activities in 44 low-income and middle-income countries: a cross-sectional analysis of nationally representative, individual-level survey data
Source: Lancet Glob Health. Author manuscript; Available in PMC 2023 Oct 7. (PMC10560068; doi:10.1016/S2214-109X(23)00348-0)
Supplement: 1 [file NIHMS1932569-supplement-1.pdf]

THE LANCET
Global Health

Supplementary appendix

This appendix formed part of the original submission and has been peer reviewed. We post it as supplied by the authors.

Supplement to: Rahim NE, Flood D, Marcus ME, et al. Diabetes risk and provision of diabetes prevention activities in 44 low-income and middle-income countries: a cross-sectional analysis of nationally representative, individual-level survey data. *Lancet Glob Health* 2023; **11:** e1576–86.

Appendix:

Appendix 1: Data Search 3
Appendix 2: Flow diagram for participant inclusion in the analysis 5
Appendix 3: Country categories* 6
Appendix 4: Country-Specific Sampling Methods 7
Appendix 5: Detailed methodology for diabetes biomarkers by country 27
Appendix 6: Fasting requirements per survey 28
Appendix 7: Detailed methodology for household wealth index calculation 29
Appendix 8: List of national, regional, or sub-regional programs with a diabetes prevention component available in 39 of the 44 LMICs included in this study 30
Appendix 9. Survey questionnaire wording and availability 35
Appendix 10: Characteristics of surveys conducted in 44 LMICs between 2010 and 2019, classified by World Bank income group 36
Appendix 11. Countries with available data for each diabetes prevention service 38
Appendix 12. Proportion deemed at high risk of diabetes, classified by geographic region 39
Appendix 13. Table of prevalence rates for the population deemed at high risk of diabetes, classified by geographic region 40
Appendix 14. Table of prevalence rates for the population deemed at high risk of diabetes, classified by World Bank income groups 42
Appendix 15. Table of proportion of the population deemed at high risk of diabetes who reported receiving physical activity counseling, classified by World Bank income groups 44
Appendix 16. Table of proportion of the population deemed at high risk of diabetes who reported receiving body weight counseling, classified by World Bank income groups 45
Appendix 17. Table of proportion of the population deemed at high risk of diabetes who reported receiving dietary fruit and vegetable counseling, classified by World Bank income groups 46
Appendix 18. Table of proportion of the population deemed at high risk of diabetes who reported receiving blood glucose screening, classified by World Bank income groups 47
Appendix 19. Univariate regression models of physical activity counseling 499
Appendix 20. Univariate regression models of body weight counseling 50
Appendix 21. Univariate regression models of dietary fruit and vegetable counseling 51
Appendix 22. Univariate regression models of blood glucose measuring 52
Appendix 23. Multivariate regression model of physical activity counseling 53
Appendix 24. Multivariate regression models of body weight counseling 54
Appendix 25. Multivariate regression model of dietary fruit and vegetable counseling 55
Appendix 26. Multivariate regression model of blood glucose measuring 56
Appendix 27. Defining high-risk for developing diabetes using the Finnish Diabetes Risk Score (FINDRISC) 57
Appendix 28. Multivariate regression model of physical activity counseling, adjusting for wealth 59
Appendix 29. Multivariate regression model of body weight counseling, adjusting for wealth 60
Appendix 30. Multivariate regression model of dietary fruit and vegetable counseling, adjusting for wealth 61
Appendix 31. Multivariate regression model of blood glucose measuring, adjusting for wealth 62
Appendix 32. Multivariate regression model of physical activity counseling, adjusting for rurality 63

Appendix 33. Multivariate regression model of body weight counseling, adjusting for rurality **64**
Appendix 34. Multivariate regression model of dietary fruit and vegetable counseling, adjusting for rurality **65**
Appendix 35. Multivariate regression model of blood glucose measuring, adjusting for rurality **66**
Appendix 36. Multivariate regression model of physical activity counseling, restricted to those considered at high risk of diabetes due to BMI and age. **67**
Appendix 37. Multivariate regression model of body weight counseling, restricted to those considered at high risk of diabetes due to BMI and age. **68**
Appendix 38. Multivariate regression model of dietary fruit and vegetable counseling, restricted to those considered at high risk of diabetes due to BMI and age **69**
Appendix 39. Multivariate regression model of blood glucose measuring, restricted to those considered at high risk of diabetes due to BMI and age **70**
Appendix 40. Table of prevalence rates for the population deemed at high risk of diabetes by FINDRISC, classified by World Bank income group **71**
Appendix 41. Proportion of the population deemed high-risk by FINRISC who self-reported having received each diabetes preventive intervention by World Bank income group **73**
Appendix 42: STROBE checklist **74**

Appendix 1: Data Search

We conducted a multistage search process in April 2021 to identify surveys included in our broader research collaboration, the Health Project on Access to Care for Cardiometabolic Diseases (HPACC; www.hpaccproject.org). For this search, we sought surveys conducted in any of the 218 countries listed in the historical World Bank income group analytic classifications (https://datahelpdesk.worldbank.org/knowledgebase/articles/906519-world-bank-country-and-lending-groups). We used the following survey inclusion criteria:

1. Nationally representative of adult population; and
2. Conducted in 2010 or later in any country or territory defined by the World Bank; and
3. Individual-level data available; and
4. Contained diabetes, blood pressure, or cholesterol biomarker data; and
5. Response rate ≥50%.

Our search process was conducted by two independent analysts using the following four-step process (Figure). Disagreements were resolved by consensus and, if needed, in consultation with a third member of our research collaboration.

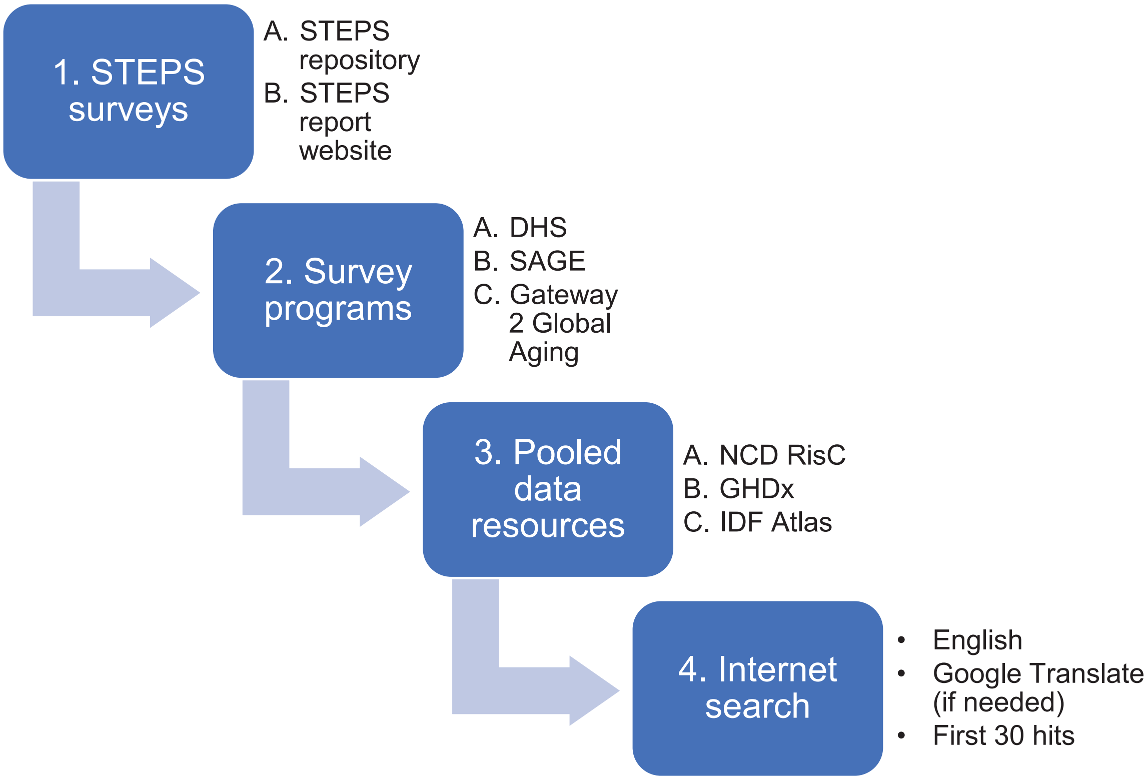

Figure: HPACC search process

1. We searched for countries in which a WHO Stepwise Approach to NCD Risk-Factor Surveillance (STEPS) survey had been conducted. We searched both the WHO STEPS Repository (https://extranet.who.int/ncdsmicrodata/index.php/catalog/STEPS) and the STEPS Country Report website (https://www.who.int/teams/noncommunicable-diseases/surveillance/data).

2. We searched for eligible surveys in the following survey programs:
   a. Demographic and Health Surveys (DHS): https://dhsprogram.com/data/available-datasets.cfm
   b. WHO Study on global AGEing and adult health (SAGE): https://apps.who.int/healthinfo/systems/surveydata/index.php/catalog/sage
   c. Gateway to Global Aging Data: https://g2aging.org/

3. We searched for eligible surveys using the following pooled data resources:
   a. NCD Risk Factor Collaboration (NCD RisC): https://ncdrisc.org/
   b. Global Health Data Exchange (GHDx): http://ghdx.healthdata.org/
   c. IDF Diabetes Atlas: https://diabetesatlas.org/

4. For countries with no identified survey in 1-3, we conducted a systematic internet search as described below:

   Search engine: Google

   Language: No language restrictions. The search was conducted in English, and all non-English hits were be reviewed using Google Translate.

   Search terms: "[country]" AND survey AND ("national" OR "population-based" OR "nationally representative") AND (diabetes OR "blood glucose" OR "plasma glucose" OR "blood sugar" OR glycated OR A1c OR HbA1c OR A1C OR "blood pressure" OR hypertension OR hypertensive OR hyperlipidemia OR hypercholesterolemia OR cholesterol OR LDL OR HDL OR lipoprotein OR triglycerides OR triglyceride OR lipid OR lipids)

   Number of hits reviewed: First 30 hits.

In this analysis, we used our research group's most recent pooled dataset, which was released on September 23, 2021. This dataset release contained n=64 country surveys conducted in 2010 or after in low- and middle-income countries as defined by the World Bank criteria in the year the survey was conducted. Of these, we included n=44 surveys with an available diabetes biomarker (glucose or hemoglobin A1c).

Appendix 2: Flow diagram for participant inclusion in the analysis

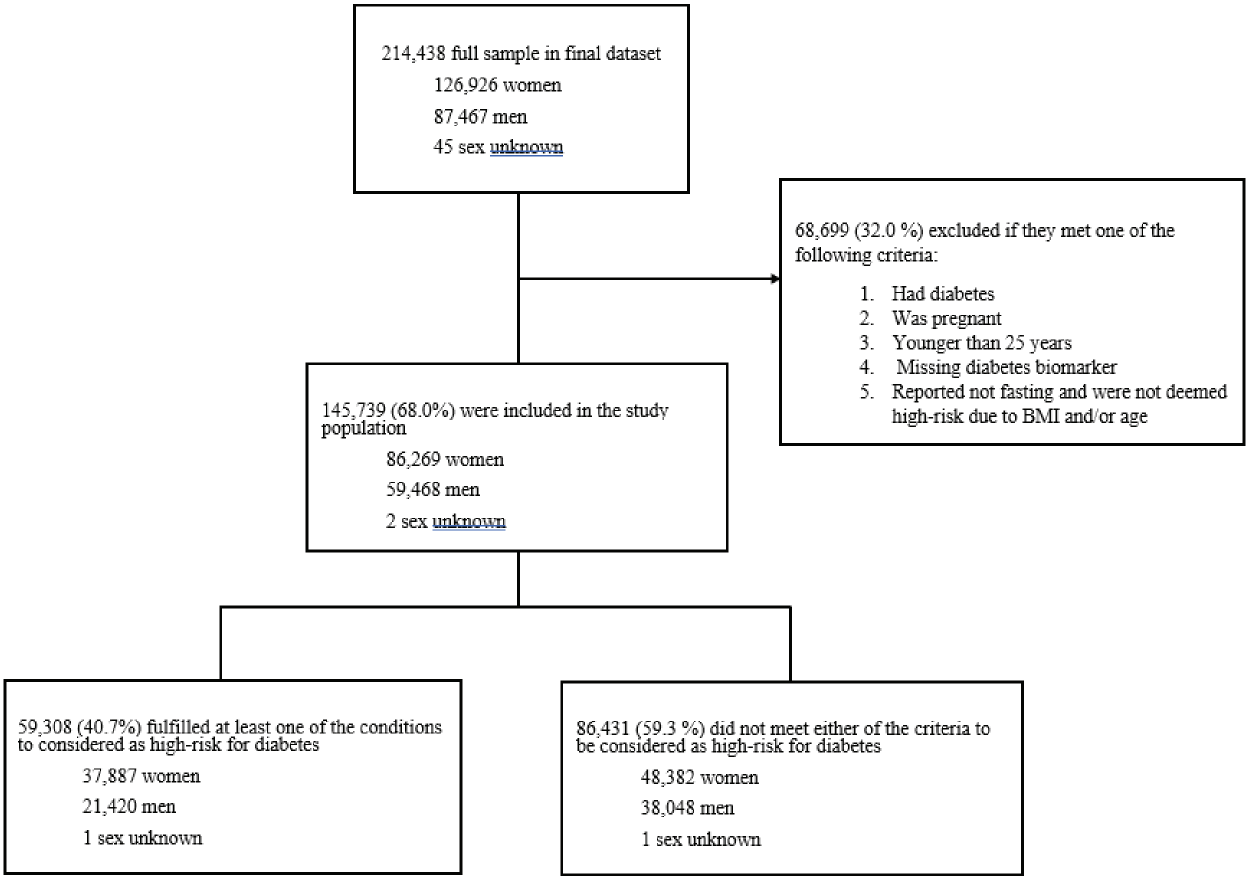

Notes: Flow diagram illustrating the exclusion criteria applied to construct of our study population. Share of individuals at each stage in relation to previous stage noted as percentages in parentheses

Appendix 3: Country categories*

World Bank income group	Countries
Low-income countries	Benin, Burkina Faso, Cambodia, Eritrea, Liberia, Rwanda, Tanzania (including Zanzibar), Togo, Uganda,
Lower-middle income countries	Bangladesh, Bhutan, Eswatini, Georgia, Kenya, Kyrgzystan, Laos, Lesotho, Moldova, Morocco, Myanmar, Nepal, Samoa, Solomon Islands, Sri Lanka, Sudan, Timor Leste, Vanuatu, Vietnam, Zambia
Upper-middle income countries	Algeria, Azerbaijan, Belarus, Botswana, Chile, Costa Rica, Ecuador, Iran, Iraq, Lebanon, Marshall Islands, Namibia, Seychelles, Tokelau, Tuvalu

Geographic region	Countries
South/East/Southeast Asia	Bangladesh, Bhutan, Cambodia, Laos, Myanmar, Nepal, Sri Lanka, Timor Leste, Vietnam
Europe and Central Asia	Azerbaijan, Belarus, Georgia, Kyrgyzstan, Moldova
Oceania	Marshall Islands, Samoa, Solomon Islands, Tokelau, Tuvalu, Vanuatu
Latin America & the Caribbean	Chile, Costa Rica, Ecuador
Middle East and North Africa	Algeria, Iran, Iraq, Lebanon, Morocco
Sub-Saharan Africa	Benin, Botswana, Burkina Faso, Eritrea, Eswatini, Kenya, Lesotho, Liberia, Namibia, Rwanda, Seychelles, Sudan, Tanzania (including Zanzibar), Togo, Uganda, Zambia

*Geographical regions were adapted from the classification scheme of the NCD Risk Factor Collaboration.[1]

1. NCD Risk Factor Collaboration (NCD-RisC). Worldwide trends in diabetes since 1980: a pooled analysis of 751 population-based studies with 4.4 million participants. *Lancet*. 2016;387(10027):1513-1530. doi:10.1016/S0140-6736(16)00618-8

Appendix 4: Country-Specific Sampling Methods

<u>Country-Specific Sampling Methods</u>

Note: In order to ensure accuracy in reporting, sampling methods are pasted verbatim from specified sources.

Algeria STEPS 2016-2017

A multi-stage cluster sample of households. One individual within the age range of the survey was selected per household. Analysis weights were calculated by taking the inverse of the probability of selection of each participant. These weights were adjusted for differences in the age-sex composition of the sample population as compared to the target population.
Different weight variables are available per Step:
wStep1 - for interview data
wStep2 - for physical measures
wStep3 - for biochemical measures
This allows for differences in the weight calculation for each Step of the survey as the age-sex composition of the respondents to each Step can differ slightly due to refusal or drop out.
Additionally, some countries perform subsampling for Step 2 and/or Step 3. When no subsampling is done and response rates do not differ across Steps of the survey, the 3 weight variables will be the same.
<u>Age range of participants included:</u> 18-69 years
Source: no report or fact sheet available. Sampling information obtained from: https://extranet.who.int/ncdsmicrodata/index.php/catalog/91/study-description

Azerbaijan STEPS 2017

A multi-stage cluster sample of households. One individual within the age range of the survey was selected per household. Analysis weights were calculated by taking the inverse of the probability of selection of each participant. These weights were adjusted for differences in the age-sex composition of the sample population as compared to the target population.
Different weight variables are available per Step:
wStep1 - for interview data
wStep2 - for physical measures
wStep3 - for biochemical measures
This allows for differences in the weight calculation for each Step of the survey as the age-sex composition of the respondents to each Step can differ slightly due to refusal or drop out.
Additionally, some countries perform subsampling for Step 2 and/or Step 3. When no subsampling is done and response rates do not differ across Steps of the survey, the 3 weight variables will be the same.
<u>Age range of participants included:</u> 18-69 years
Source: no report or fact sheet available. Sampling information obtained from: https://extranet.who.int/ncdsmicrodata/index.php/catalog/127/studydescription#page=overview&tab=study-desc

Bangladesh: STEPS 2018

A cross–sectional survey was carried out from February to May 2018 among adult population aged l8-69 years including men and women residing in the households in all the divisions of Bangladesh. Sampling was done by multistage, geographically stratified probability based sampling on the basis of Primary Sampling Unit (PSU) developed by Bangladesh Bureau of Statistics (BBS) for census 2011. The sample size was calculated considering prevalence of different NCD risk factors, relative precision rate and feasibility of the survey. To calculate the final sample size, the design effect and non-response rate at the household and individual level were considered. Considering the findings of Demographic Health survey and previous BBS surveys, the person non-response rate and household non-coverage rate and design effect, security issue and non-clearance of local administration, the final adjusted sample size was 9,900 adults of 495 PSUs. However, based-on eligibility, refusal etc. finally,

out of 9900 complete data were gotten from 8185 respondents, physical measurements could be done in 7208 participants, and blood and urine sample was collected from 7065 and 7028 respondents respectively. Both the blood and urine samples were given by 6901participants.
Age range of participants included: 18-69 years
Source: https://extranet.who.int/ncdsmicrodata/index.php/catalog/770

Belarus: STEPS 2016
The sampling frame is a collection of data and materials from which are selected for the survey. The optimal sampling frame should be complete, accurate and current. Best of all, the above criteria are met by the results of the population census, which became the basis for constructing the sample for the STEPS study. Census population represents a representative territorial sampling frame in the form a hierarchical set of parcels grouped in a certain way. Plots censuses are, on average, about the same size. For each site there is a schematic map that provides a clear, non-overlapping demarcation of geographic districts, as well as information on the population and the number of households.

The largest in size is the census area, which includes several instructor sites. The smallest unit in the hierarchical structure of parcels by censuses - enumeration areas.A positive aspect of using enumeration areas as primary sampling units (PSUs) is that they have a small and approximately the same size (each includes about 100 HHs on average). Consequently this, the PSU is a territory within which it is possible to effectively organize field work. To conduct a population census, the territory of the Republic of Belarus was divided into almost 32 thousand enumeration areas. Due to the fact that the last population census in the Republic of Belarus was carried out in 2009, to update the sample, the current data of polyclinics were used, medical outpatient clinics, FAPs and rural Soviet accounting in rural areas.
Age range of participants included: 18-69 years
Source: Translated directly from the Belarus STEPS 2016 report. Available at: https://extranet.who.int/ncdsmicrodata/index.php/catalog/100/related_materials

Benin: STEPS 2015
"The STEPS survey on risk factors for non-communicable diseases in Benin was conducted from October to December 2015. It was a population-based survey of adults aged 18 to 69 years. A 3-stage sampling frame was used to produce representative data for this age group in Benin. The information required for the investigation was collected electronically using a manual device. The survey was implemented by the National Program for the Fight against Non-Communicable Diseases (PNLMNT) of the Ministry of Health of Benin. A total of 5,126 adults participated in the STEPS survey conducted in Benin. The overall response rate was 98.6%. The 1st survey took place in 2008. A third survey is planned for 2020 if the financial situation allows it."
Age range of participants included: 25-64 years
Source: Translated directly from the Benin STEPS 2015 report. Available at: https://extranet.who.int/ncdsmicrodata/index.php/catalog/107/download/1044.

Bhutan: STEPS 2014
"To achieve a nationally representative sample, a multistage sampling method was used to select enumeration areas, households and eligible participants at each of the selected households in three stages. The 2005 National Census was chosen as the basis for the sampling frame, with "Geogs" (blocks) in rural areas and towns in urban areas forming the primary sampling units (PSUs). Since the population distribution for urbanicity is 70:30 (rural:urban), 63 PSUs in rural and 14 PSUs in urban areas were chosen. PSUs were selected through the probability proportionate to size (PPS) sampling using the number of households in each PSU. Two secondary sampling units (SSUs) for every rural PSU and 4 SSUs for every urban PSU were selected. This led to the selection of 126 SSUs from rural and 56 SSUs from urban areas. This was also carried out by PPS sampling, using the number of households in each SSU. A total of 16 households from each SSU (both rural and urban) were selected using systematic random sampling. The sampling frame for this was the list of households with a

unique identification number (ID) developed by the enumerators for the survey. At the household level, the Kish sampling method was used to randomly select one eligible member (aged 18–69 years) of the household for the survey. The Kish method ranks eligible household members in order of decreasing age, starting with males and then females, and randomly selects a respondent using the automated program for Kish selection in the handheld personal digital assistant (PDA)."
Age range of participants included: 18-69 years
Source: National survey for noncommunicable disease risk factors and mental health using approach WHO Steps Approach in Bhutan – 2014. Available at: http://www.who.int/chp/steps/bhutan/en/. Additional reference: World Health Organization Regional Office for South-East Asia. National survey for noncommunicable disease risk factors and mental health using WHO STEPS approach in Bhutan—2014. Geneva: World Health Organization; 2014.

Botswana STEPS 2014
Botswana has a population of over 2 million with 27 districts and 4,845 enumeration areas and sample size of 300 enumeration areas with a target population of 6,400 people was systematically drawn from a pool of the whole enumeration areas. Against the identified enumeration areas numbers of households were listed and proportion of participants was calculated from the total sample size required for the country. Finally a computer generated random number was drawn to go into specific households in that specific enumeration area and at the end eligible participants residing in the household were listed into the electronic hand held data assistant (PDA) and at the end a name was picked automatically to participate in the survey.
Age range of participants included: 15-69 years
Source: Botswana STEPS report. Available at: https://extranet.who.int/ncdsmicrodata/index.php/catalog/318

Burkina Faso: STEPS 2013
"Sampling methodology: The study was conducted on a sample obtained from a three-stage cluster stratified as recommended by the WHO STEPS. The sampling frame used was that derived from the general census of the population and habitat 2006 (RGPH 2006) and updated in 2010 during the survey Demographic and Health Survey of Burkina Faso (EDS-BF, 2010). This update concerned the enumeration areas (EAs) that correspond to the cluster as part of this study.
Selection of clusters: The choice of clusters was made according to a systematic random selection proportional to their size (in number of households) within strata (regions). To do this clusters were organized by stratum and place of residence (urban / rural). A total of 240 clusters of which 185 were in rural areas and 55 in urban areas were selected for the investigation.
Selection of households: Households were randomly drawn after an enumeration exhaustive list of all households in the cluster. A draw tool designed on Excel by the team. The technique was used in the field for selecting households to investigate. In total, 20 households in clusters were selected to participate in the study.
Selection of individuals: The choice of individuals was made randomly using Kish's method. In total, an individual aged 25 to 64 living in a selected household was fired for participate in the survey."
Age range of participants included: 25-64 years
Source, translated from: Rapport de l'enquete national sur la prevalence des principaux facteurs de risques communs aux maladies non transmissibles au Burkina Faso Enquete STEPS 2013. Available at: http://www.who.int/chp/steps/burkina_faso/en/.

Cambodia STEPS 2010
"The initial planned sample size was designed to involve 5,760 persons in accordance with the NCD multi-stage cluster survey method (1.5 design effect, 95% confidence interval, 5% margin or error, and 50% baseline levels of the indicators) in order to provide an equivalent distribution of the participants in regards to age groups and gender after taking into consideration that the estimated potential rate for non-response in each group and refusals in the nest stages would equal to 20%. Estimates were obtained for each of the following eight age/sex groups: men ahed 25-34 years, 35-44 years, 45-54 years, and 55-64 years; and women aged 25-34 years, 35-44 years, 45-54 years, and 55-64 years.

The survey was designed to cover all geographical areas of Cambodia and a 3-stage sampling process as part of the multi-stage cluster sampling was carried out to randomly select the target population: random selection of communes (Khum in rural areas and its equivalent Sangkat in urban area) as primary sampling unit (PSU), followed by villages (Phum) for the second sampling unit (SSU), and by households for the elementary units (EU). Finally, all members of the randomly chose households aged 25-64 years were invited to participate in this survey. The selection process was performed identically for urban and rural areas in order to get a self-weighted estimate for the whole population of the country. A total of 180 clusters with 34 clusters from the urban area and 146 clusters from the rural area were randomly selected."
Age range of participants included: 25-64 years
Source: Cambodia STEPS 2010 survey report. Available at: https://www.who.int/ncds/surveillance/steps/cambodia/en/

Chile: NHS 2009-10
"The sampling frame was constituted from the Population and Housing Census 2002. The design of the study was transversal, with a random sample of complex type households (stratified and multi-stage by clusters) with national, regional and area representation rural / urban. The target population was adults older than or equal to 15 years. The survey had a response rate in the eligible population of 85%. The refusal rate was of 12%. 5,434 people were interviewed. A nurse performed clinical and examinations to 5,043 participants and 4,956 accepted laboratory tests (blood and urine). The total sample loss of the oversized sample was 28% (this including rejection, non-contact and other causes of random loss). The raw sample was designed with overrepresentation of some population groups (older adults, regions other than the Metropolitan Region and rural areas) to increase sample efficiency and homogenize the accuracy of the estimators. The expansion of the sample data is because it grants each participant the weight that corresponds to it according to the design sample and at the same time corrects the distortion of the raw sample, making it coincide with the census population projection for January 2010 for Chilean adults over 15 years of age."
Age range of participants included: 15 years or older
Source, translated from: Resumen Ejecutivo: Encuesta Nacional de Salud ENS Chile 2009-10. Available at: http://epi.minsal.cl/encuesta-ens-anteriores/.

Costa Rica: STEPS 2010
"The Costa Rican NCRFSS survey was a cross-sectional survey based on a probabilistic cluster sampling design. The NCRFSS survey was conducted during 2010 under the supervision of the Caja Costarricense de Seguro Social, a government public healthcare provider, and covers the overall adult population aged ≥20 years. Multistage cluster sampling was performed stratified by geographical areas, age groups (20–39, 40–64, and ≥65 years) and gender. The first sample stage was the randomized selection of the country's geographical areas as primary sample units followed by the random selection of sectors in selected areas as secondary sample units. The random selection of areas and sectors was performed with probability proportional to size; the area or sector size was determined by the population >20 years during 2009, as estimated by the Costa Rican Census and Statistics National Institute (INEC). Households were chosen through a random number generator using dwelling lists obtained from the health technician assistant in every community until all age group and gender strata sample sizes were achieved. A family dwelling was defined as a group of people who share the same table to eat. Survey participants were selected by the Kish method, which samples participants within a household with equal probability of selection, as recommended by the WHO STEPwise methodology. To be eligible for inclusion in the study, subjects had to be ≥20 years of age, permanently residing in the selected homes, and to have provided written consent. Pregnant or lactating mothers and those who were within 6 months postpartum were excluded from the study. Each participant selected for the study was informed of the study objectives and details before agreeing to participate in the investigation. In all, 3653 noninstitutionalized adults were surveyed, with an 87.8% response rate of the eligible population."
Age range of participants included: 20 years or older

Source: Wong-McClure R, Gregg EW, Barcelo A, Sanabria-Lopez L, Lee K, Abarca-Gomez L, Cervantes-Loaiza M, Luman ET. Prevalence of diabetes and impaired fasting glucose in Costa Rica: Costa Rican National Cardiovascular Risk Factors Survey, 2010. J Diabetes. 2016 Sep;8(5):686-92.

Ecuador: STEPS 2018

The STEPS sample design used probability sampling techniques to ensure the geographic representativeness and of the study domains of the survey, and to calculate the factors expansion and errors associated with sampling. The target population or study universe included the total of adults aged 18 to 69 years, disaggregated by men and women, residents in the territory of Ecuador, except Galapagos. According to the INEC population projection, it included 10,249,369 people. The unit of observation and elementary unit of analysis were people between 18 and 69 years of the territory Ecuadorian, except Galapagos. The sampling frame for the STEPS Survey was defined from the Sampling Frame for household surveys of the National Institute of Statistics and Censuses - INEC. Nevertheless, due to the scope of the MSP-INEC inter-institutional cooperation agreement, the information cartographic information for gathering information was restricted to that used for the Census of Population and Housing - CPV 2010. The delimited frame contains mainly variables of ID; location variables; stratification variables; and, design and control variables selection units. Sample selection. The selection of PSUs, according to the established size, was carried out independently in a random way in each of the strata. They were also selected randomly 12 dwellings from each previously selected cluster. From second period of uprising, given the high rates of occupation change, 16 were chosen homes per cluster, to counteract this effect. The change affected the 230 remaining conglomerates, giving a total of 6,680 dwellings to be surveyed. Finally, a enlistment of the eligible persons within each dwelling, selecting in a manner
random one of them.

For the STEPS Ecuador 2018 survey, the standard STEPS version 3.2 instrument was used in Spanish, revised and adapted for the Ecuadorian context by the MSP, INEC and PAHO / WHO. All three steps were included:

- Step 1 - questionnaires on the behavior of tobacco and alcohol consumption, consumption
- of fruits, vegetables and salt, practice of physical activity, and history of measurement and diagnosis of hypertension, diabetes and high blood cholesterol. The modules were also applied options of: colon, prostate and breast cancer health screening, tobacco, and oral health.
- Step 2 - anthropometric data (weight, height, waist circumference, BMI, blood pressure).
- Step 3 - biochemical data (glucose and total cholesterol in capillary blood).

<u>Age range of participants included:</u> 18-69 years

Source: Translated from Ecuador STEPS 2018 report: https://extranet.who.int/ncdsmicrodata/index.php/catalog/774

Eritrea STEPS 2010

"A multi-stage cluster sample of households. One individual within the age range of the survey was selected per household.

Analysis weights were calculated by taking the inverse of the probability of selection of each participant. These weights were adjusted for differences in the age-sex composition of the sample population as compared to the target population.

Different weight variables are available per Step:
wStep1 - for interview data
wStep2 - for physical measures
wStep3 - for biochemical measures

This allows for differences in the weight calculation for each Step of the survey as the age-sex composition of the respondents to each Step can differ slightly due to refusal or drop out. Additionally, some countries perform subsampling for Step 2 and/or Step 3. When no subsampling is done and response rates do not differ across Steps of the survey, the 3 weight variables will be the same."

<u>Age range of participants included:</u> 25-74 years

Source: no report available. Sampling information obtained from: https://extranet.who.int/ncdsmicrodata/index.php/catalog/589/study-description#page=sampling&tab=study-desc

Eswatini: STEPS 2014
"A Multi-stage cluster sampling design was applied. The survey covered all the four regions of the country. The size of the country and the distances between the regions and communities made it possible for the survey to sample a population representing all the 4 regions. The Multi-stage sampling procedure was implemented in the following procedural steps:

Stage 1: All four regions were included as a sampling frame of our Primary Sampling Unit (PSU).The number of the PSUs at this stage ensured precision in the survey estimates and as a result 216 PSUs were selected using probability proportional to size sampling.

Stage 2: The second stage of cluster sampling procedure entailed listing, sorting and random systematic sampling of the Secondary Sampling Units (Households) within the PSUs selected in stage1 where 20 households were selected from each PSU. Based on census data, only households with eligible participants were systematically sampled through random systematic sampling.

Stage 3: At this level, all the eligible participants within a household were sequentially listed into the PDAs and only one participant per household was randomly sampled using KISH method built into the PDAs. The KISH method is a widely used technique that uses a pre-assigned table of random numbers to identify the person to be interviewed."
<u>Age range of participants included:</u> 15 to 69 years
Source: WHO STEPS: Noncommunicable Disease Risk Factor Surveillance Report Swaziland 2014. Available at: http://www.who.int/chp/steps/swaziland/en/.

Georgia: STEPS 2016
"The STEPS survey of noncommunicable disease (NCD) risk factors in Georgia was carried out from June 2016 to September 2016. Georgia carried out Step 1, Step 2 and Step 3. Socio demographic and behavioural information was collected in Step 1. Physical measurements such as height, weight and blood pressure were collected in Step 2. Biochemical measurements were collected to assess blood glucose and cholesterol levels in Step 3. The survey was a population-based survey of adults aged 18-69. A Multi-stage cluster sampling design was used to produce representative data for that age range in Georgia. A total of 5554 adults participated in the survey. The overall response rate was 75.7%."
<u>Age range of participants included:</u> 18 to 69 years
Source: Georgia STEPS Survey 2016 Fact Sheet.
Available at: http://www.who.int/chp/steps/georgia/en/.

Iran: STEPS 2016
"The sampling part, which includes determining the sample size and the cluster head, belongs to the pre-study phase and was planned in the form of a specific protocol for sample size and statistical sampling. All experts in the quality control team supervised the finding of samples and cluster heads. In order to estimate the prevalence rate of the risk factors for non-communicable diseases in the country in 1395, a sampling method proportionate to the population was used, which is a common approach in survey studies. Therefore, the selected sample size was proportionated to the population of that province. On the other hand, for estimating the prevalence of the risk factors in the province, in order to be on the safe side, the smallest sample size for achieving the predicted rates was calculated at 95%. This rate was equal to 384 samples, which was selected as the smallest sample size in the least populated province, Ilam. The required sample size for other provinces was therefore calculated according to the population of that province proportionate to the population of the reference province, Ilam. Besides, to control the non-response error, 10% was added to the calculated sample size in each province. In order to decrease costs and increase efficiency, for provinces with 800 samples or more, weights were given to their samples. Weight-giving is an effective method used in surveys in order to

decrease the sample size. This was achieved in the selected provinces by considering the calculated sample size as half and the sampling weight as double. The total sample size was calculated to be 30150 and to achieve this sample size, sampling from 3015 clusters was required."
Age range of participants included: 18 and older
Source: Iran STEPS 2015 report.
Available at: https://www.who.int/ncds/surveillance/steps/STEPS_2016_Atlas_EN.pdf?ua=1

Iraq: STEPS 2015
"The sample frame consisted of the population of Iraq of (18+) years for both sexes residing in the urban and rural area. It was based on the results of listing and numbering operation for the year 2009 that covered all governorates. Due to the unstable conditions at the time of the survey three governorates (Naynawa, Salahaddin and Al-Anbar) were excluded. A major challenge confronted was the late demographic change due to population movement, displacement and migration. All permanent residents of (18+) years of age, who were resident in Iraq within one month at the time of implementation of the survey were considered eligible.

A cross-sectional community based survey covering 15 governorates in Iraq. A Multi-stage cluster sampling technique was depended to select the minimum representative sample size to estimate the prevalence of the risk factors of noncommunicable disease through direct interview, physical examination and laboratory examination of blood samples of study participants. A total of 412 clusters were randomly selected each contain ten households. One subject from each household was randomly selected using KISH table to participate in the survey with a total sample size of 4120. The Sample was designed to provide estimates on a number of indicators on the situation of Noncommunicable diseases risk factors in Iraq at the national level. A national based rather than a governorate based sample is selected. A multi stage cluster sampling was used with stratification to urban and rural areas. Primary sampling units (PSUs) were the blocks, which consisted of 70 households or more before selection."
Age range of participants included: 18 years and older
Source: Iraq STEPS 2015 report.
Available at: https://www.who.int/ncds/surveillance/steps/Iraq_2015_STEPS_Report.pdf

Kenya: STEPS 2015
"The 2015 Kenya STEPs survey was a national cross-sectional household survey designed to provide estimates for indicators on risk factors for non-communicable diseases for persons age 18 – 69 years. The sample was designed with a sample size of 6,000 individuals to allow national estimates by sex (male and female) and residence (urban and rural areas). The survey used the fifth National Sample Surveys and Evaluation Programme (NASSEP V) master sample frame that was developed and maintained by KNBS. The frame was developed using the Enumeration Areas (EAs) generated from the 2009 Kenya Population and Housing Census to form 5,360 clusters split into four equal sub-samples. A three-stage cluster sample design was adopted for the survey involving selection of clusters, households and eligible individuals. In the first stage, 200 clusters (100 urban and 100 rural) were selected from one sub-sample of NASSEP V frame. A uniform sample of 30 households from the listed households in each cluster was selected in the second stage of sampling. The last stage of sampling was done using Personal Digital Assistants (PDAs) at the time of survey, where one individual was randomly selected from all eligible listed household members using a programmed KISH method of sampling."
Age range of participants included: 18 to 69 years
Source: WHO: Kenya STEPwise Survey for Non Communicable Diseases Risk Factors 2015 Report.
Available at: http://www.who.int/chp/steps/Kenya_2015_STEPS_Report.pdf?ua=1.

Kyrgyzstan: STEPS 2013
A multi-stage cluster sample of households. One individual within the age range of the survey was selected per household.

Analysis weights were calculated by taking the inverse of the probability of selection of each participant. These weights were adjusted for differences in the age-sex composition of the sample population as compared to the target population.

Different weight variables are available per Step:
wStep1 - for interview data
wStep2 - for physical measures
wStep3 - for biochemical measures
This allows for differences in the weight calculation for each Step of the survey as the age-sex composition of the respondents to each Step can differ slightly due to refusal or drop out.
Age range of participants included: 25 to 64 years
Source: no report or fact sheet available. Sampling information obtained from: https://extranet.who.int/ncdsmicrodata/index.php/catalog/271/study-description#page=overview&tab=study-desc

Lao People's Democratic Republic: STEPS 2013
A multi-stage cluster sample of households. One individual within the age range of the survey was selected per household. Analysis weights were calculated by taking the inverse of the probability of selection of each participant. These weights were adjusted for differences in the age-sex composition of the sample population as compared to the target population.

Different weight variables are available per Step:
wStep1 - for interview data
wStep2 - for physical measures
wStep3 - for biochemical measures
This allows for differences in the weight calculation for each Step of the survey as the age-sex composition of the respondents to each Step can differ slightly due to refusal or drop out. Additionally, some countries perform subsampling for Step 2 and/or Step 3. When no subsampling is done and response rates do not differ across Steps of the survey, the 3 weight variables will be the same.
Age range of participants included: 18 to 64 years
Source: no report or fact sheet available. Sampling information obtained from: https://extranet.who.int/ncdsmicrodata/index.php/catalog/588/study-description#page=sampling&tab=study-desc

Lebanon: STEPS 2017
"A national cross-sectional survey adopting a two-stage cluster sampling design was conducted for Steps 1, 2 and 3. The sampling frames references used were the population distribution in Lebanon 2014, retrieved from the Central Administration for Statistics (CAS) and the Syrian population distribution data 2015, retrieved from UNHCR. 144 clusters were selected for the Lebanese sample and 144 clusters for the Syrian sample. The Primary Sampling Units (PSUs) were cadastral areas (cadasters) and the Secondary Sampling Units (SSUs) were the households. Twenty participants were recruited from each cluster. The latest available population estimates (cadastral data) were used, to randomly recruit PSUs by Probability Proportionate to Size (PPS). To account for the issue of the variability in the cadasters' sizes, very small cadasters (<200 individuals) were combined with neighboring PSUs before selecting the sample, to enhance the likelihood of finding 20 target participants. On the other hand, cadasters with a large population size that were guaranteed to be sampled at least twice were handled as strata and each stratum were assigned a fixed number of random starting points based on how often it was selected with certainty. This was done using satellite images divided into grids, previously obtained from the Centers for Disease Control and Prevention (CDC) for all Lebanese cadasters.

For the Lebanese sample, the research team relied on the standard Expanded Program for Immunization (EPI) method for a systematic random selection of the households. Accordingly, within

each selected PSU, households were identified using a systematic random approach following the WHO-UNICEF-EPI cluster method. The fieldworkers started with the highest floor on the right side of a building. If the household hosted an eligible participant, they proceeded with data collection, if not, they visited a second household which is selected by skipping 5 households. If during sampling, non-Lebanese households were selected, the fieldworker skipped them in a straight line until a Lebanese household was identified. This method has been previously used for national surveys in Lebanon. One participant was randomly selected within each household, using the eSTEPS application. Households were chosen until the target of 20 participants was reached.

The PSUs for the Syrian refugees' sample were identified, using the most recent available refugee estimates to randomly recruit PSUs by PPS. The same measures aforementioned were done to account for the variation in the cadasters' sizes. The WHO-UNICEF- EPI cluster method was employed to select households. The fieldworkers targeted Syrian households; accordingly, when during sampling, non-Syrian households were selected, the fieldworker skipped them in a straight line until a Syrian household was identified. One participant was randomly selected within each household, using the eSTEPS application.

For both samples, following STEPS' team recommendations, sampling of participants was done without replacement, i.e. once a person was selected that person was not replaced with another one. Efforts were made to include all selected households. If the house was unoccupied at the time of the visit or if an adult was not available for an interview at the time of the visit, that house was revisited up to 4 times, with different visiting times. The number of refusals and non-responses was recorded."
Age range of participants included: 18 to 69 years
Source: Lebanon STEPS 2016-2017 report. Available at:
https://www.who.int/ncds/surveillance/steps/Lebanon_STEPS_report_2016-2017.pdf?ua=1

Lesotho STEPS 2012
A multi-stage cluster sample of households. One individual within the age range of the survey was selected per household. Analysis weights were calculated by taking the inverse of the probability of selection of each participant. These weights were adjusted for differences in the age-sex composition of the sample population as compared to the target population.
Different weight variables are available per Step:
wStep1 - for interview data
wStep2 - for physical measures
wStep3 - for biochemical measures
This allows for differences in the weight calculation for each Step of the survey as the age-sex composition of the respondents to each Step can differ slightly due to refusal or drop out. Additionally, some countries perform subsampling for Step 2 and/or Step 3. When no subsampling is done and response rates do not differ across Steps of the survey, the 3 weight variables will be the same.
Age range of participants included: 25-64 years
Source: Source: no report available. Sampling information obtained from:
https://extranet.who.int/ncdsmicrodata/index.php/catalog/491/study-description#page=sampling&tab=study-desc

Liberia: STEPS 2011
"Random multi-cluster sampling method was used to collect data during this survey in 5 of the 15 counties of Liberia with the district serving as the primary sampling unit. Different sampling frames were designed and used at the district (Primary Sampling Unit-PSU), Chiefdoms (Secondary Sampling Unit-SSU) and household levels. Households listing generated from the 2008 National Population Census was used, and in each household, the list of individuals' resident was obtained and the Kish Method was used. Kish Method is a household sampling technique developed by WHO for STEPS. The field team selected households by using nutrition sampling method (throwing a pencil to get a selected direction). When the household enumeration sampling point is established, the interviewer

counts all the households and using interval sample to get the household number. In each household, one person was selected using the Kish method."
Age range of participants included: 25 to 64 years
Source: WHO: The Final Report on the Liberia STEPS Survey 2011. Available at: http://www.who.int/chp/steps/Liberia_2011_STEPS_Report.pdf?ua=1.

Marshall Islands: STEPS 2017
Participants eligible for the RMI Hybrid survey will include all RMI residents aged 18 years and over residing in Majuro, Kwajalein, Arno, Jaluit, Wotje, and Kili who were able to comprehend either English or Marshallese and provide consent. Data collection began on July 7, 2017 and ended on April 5, 2018. A total of 2,869 respondents completed the survey and measurements. All interviews and measurements were performed by trained surveyors recruited by the Marshall Islands Epidemiology Prevention Initiative (MIEPI). The original sample included 3107 adults. Sample size was determined based on overall adult populations on selected islands in the Republic of the Marshall Islands. (Majuro = 1659; Ebeye = 627; Kili = 200; Wotje = 207; Jaluit = 207; Arno = 207). The final response rate was 92.3%).
Sampling procedures:
Stage 1: Households were identified at random according to geographical stratification in Majuro and Ebeye. The country was stratified into two major groups, Urban (Majuro and Ebeye) and Rural (all outer islands). In Majuro and Ebeye, household cluster sampling was used to randomly select households in these areas. Stage 2: In Majuro and Ebeye, one individual was selected at random from each household using the KISH table method. All adults in Kili, Arno, Wotje, and Jabwor, Jaluit atolls were included in the sample because the adult populations are about 200 each on these atolls."
Age range of participants included: 18 years and older
Source: Marshall Islands STEPS 2017 Report. Available at: https://extranet.who.int/ncdsmicrodata/index.php/catalog/742

Moldova: STEPS 2013
"A total of 4807 randomly selected respondents participated in the survey. They were all aged 18–69 years, and the group comprised both sexes, as well as residents of all districts and the territorial administrative unit "Gagauz-Yeri", along with Chişinău and Balti municipalities. The survey did not cover the districts from the left bank of the Nistru River and the municipality of Bender. A two-stage cluster sampling procedure was carried out to select randomly participants from among the target population. Cluster sectors from the 2004 Moldova Population Census were used as a basic unit. Given the differences in lifestyle and disease status between populations in urban and rural areas, the target population was stratified into urban and rural areas of residence for the STEPS survey. At the first stage, within each stratum, primary sampling units (PSUs) (enumeration areas (EAs)) were selected systematically with probability proportional to the 2004 Population Census EAs (measure of size equal to the number of population in the EAs, provided by the census). Before selection, the census sectors were sorted geographically from north to south within each stratum, in order to ensure additional implicit stratification according to geographical criteria. A total of 400 clusters representing 400 EAs were selected from the 10 991 census EAs. These probabilistically selected clusters were used also in Moldova's DHS conducted in 2005, and the Multiple Indicator Cluster Surveys (MICS) conducted in 2012. Cartographic materials from the Population Census conducted in Moldova in 2004 were not available, thus it was not possible to use them for the STEPS survey. Therefore, for the first stage the probabilistic samples from the abovementioned surveys were used.

Out of the 400 selected clusters, 167 were rural and 233 were urban. The distribution of the sample of 400 PSUs (EAs) for the DHS/MICS surveys was inversely proportional to the number of population within each stratum, taking into account that the response rate is lower in urban areas than rural owing to the smaller average size of the households in urban areas compared with rural areas. Thus, disproportional allocation with oversampling for urban areas was applied in the STEPS survey. A final weighting adjustment procedure was carried out to enable estimates at national and urban/rural levels.

At the second stage, 15 households (secondary sampling units (SSUs)) were selected within each of the 400 PSUs. From the updated list of households used for the MICS 2012 survey, 15 households were selected randomly per cluster, using the Microsoft Excel® random sample tool. A total of 6000 individuals were selected from among the 400 clusters. The Kish method *(17)* was applied for the random selection of one individual aged 18–69 years from each household."
<u>Age of participants included:</u> 18-69 years
Source: Republic of Moldova STEPS 2013 report. Available at: https://www.who.int/ncds/surveillance/steps/Moldova_2013_STEPS_Report.pdf

Morocco STEPS 2017
One of the essential elements for establishing a probability sampling plan is the constitution an adequate sampling frame. For the purpose of the STEPS survey, the sampling frame used to meet the sampling need was the 2014 master sample, developed by the HCP based on data from the 2014 population and housing census. It has the advantage extrapolate the sample results to the target population and estimate the accuracy desired. The stratification of observation units belonging to any sampling frame makes it possible to design sampling plans ensuring optimal sample size; a significant reduction in costs and a substantial improvement in the accuracy of expected estimators. However, the choice of criteria allowing the population to be divided into homogeneous groups (strata) and having recent and reliable data on these criteria is a task that requires generally considerable efforts (updating the sampling frame) both in terms of methodological than that of data collection.

In Morocco, the particularity of cities containing several social categories for which, synthesizing the vector of heterogeneous demographic and socioeconomic behavior into a representative characteristic makes stratification a difficult task. The stratification adopted was geographical for the two environments according to the weight in terms of households, each of which has a specific stratification: For urban units, the criteria used were the administrative division into regions, provinces / prefectures and the dominant habitat type. As for the rural environment, the primary units were stratified according to the geographical criterion, and the type of relief dominant at the municipal level.
<u>Age range of participants included</u>: 18 years and older
Source: Morocco STEPS report [translated online]: https://extranet.who.int/ncdsmicrodata/index.php/catalog/544/study-description

Myanmar STEPS 2014
To achieve a nationally representative sample, a multi-stage sampling method was used to select townships, wards and villages, households and eligible participants at each of the selected households.
Stage 1: Selection of primary sampling units (PSUs)
Administratively, Myanmar is divided into 330 townships. A township is subdivided into wards for urban settings and village tracts and then villages for rural settings. The list of townships has been used as the sampling frame at the first stage of sampling. Townships form the Primary Sampling Units (PSUs). Out of the total 330 PSUs, 52 PSUs were selected using Probability Proportionate to Size of population in each PSU (PPS).
Stage 2: Selection of Secondary Sampling Units (SSUs)
From each selected PSU (township), 6 SSUs (wards and villages) were chosen using probability proportionate to population size, totaling 312 SSUs for the whole country.
Stage 3: Selection of eligible participants at household level
From each selected SSU (ward/village), 30 households were selected using systematic random sampling. The sampling frame for this sampling is the list of households with unique identification number (ID) developed from a recent listing of households available from the Basic Health Staff.
Stage 4: Selection of eligible participants at household level
One eligible participant (aged between 25 and 64 years) in the selected households was recruited for the survey. The Kish sampling method was used to randomly select one eligible member of the household. Using the Kish Method, eligible participants (adults aged 25 to 64

years) in each household were ranked in order of 8 decreasing age, starting with males then females, then randomly selected using the automated program for Kish selection in the handheld PDA. Each PSU (township) was estimated to contribute 180 participants, totaling **9,360** participants for 52 selected townships for the whole country. In actual study, the total sample size was 8757 participants.
Age range of participants included: 18 years and older
Source: STEPwise approach to chronic disease risk factor surveillance report 2014. Available at: https://www.who.int/ncds/surveillance/steps/myanmar/en/

Namibia: DHS 2013
"The sample for the 2013 NDHS was a stratified sample selected in two stages. In the first stage, 554 EAs were selected with a stratified probability proportional to size within the sampling frame. The EA size is the number of households residing in the EA and recorded in the 2011 NPHC. Stratification was achieved by separating each region into urban and rural areas. Therefore, the 13 regions were stratified into 26 sampling strata: 13 rural strata, and 13 urban strata. Samples were selected independently in each stratum, with a predetermined number of EAs selected as shown in Table A.3. Implicit stratification with proportional allocation was achieved at each of the lower administrative unit levels by sorting the sampling frame before the sample selection. Sorting was done according to the constituency and the EA code within a sampling stratum, and by using a probability proportional-to-size selection procedure.

After the selection of EAs and before the main survey, a household listing operation was carried out in all selected EAs, and the resulting lists of households served as a sampling frame for the selection of households in the second stage. Some of the selected EAs may large. To limit the amount of work done to list each household, selected EAs with more than 200 households were segmented by the listing team in the field before the household listing. Only one segment was selected for the survey, with probability proportional to the segment size. Household listing was conducted only in the selected segment (see detailed instructions for segmentation in the DHS Manual for Household Listing). So a 2013 NDHS cluster is either an EA or a segment of an EA. In the second-stage selection, a fixed number of 20 households was selected in every urban cluster and rural cluster, by equal probability systematic sampling. A spreadsheet indicating the selected household numbers for each cluster was prepared. The survey interviewers interviewed only the pre-selected households. To prevent bias, no replacements and no changes of the pre-selected households were allowed in the implementing stages. In half of the selected households where there was no male survey, all women age 15-49 were interviewed; in the other half of the selected households where there was a male survey, all males and females age 15-64 were interviewed."
Age range of participants included: women 15 to 64 years
Source: The Nambia Ministry of Health and Social Services (MoHSS) and ICF International. 2014. The Namibia Demographic and Health Survey 2013. Windhoek, Namibia, and Rockville, Maryland, USA: MoHSS and ICF International.

Nepal: STEPS 2019
"Survey population included men and women aged 15-69 years who have been the usual residents of the household for at least six months and have stayed in the household the night. The nationally representative sample was selected through multistage cluster sampling. Sampling frame consisting of the distribution of old wards as in census 2011 was obtained from the Central Bureau of Statistics (CBS). A total of 25 households were sampled from each of the clusters. A sampling frame of all the households in the samples PSUs was obtained through a complete household listing and mapping carried out in the samples PSUs in September 6 to December 6 2018. Sampling frame for selection of households from each PSU was prepared by conducting household listings and mapping. The team of enumerators visited the sampled PSUs and carried out a complete mapping of all the households in the PSU. The lists of the households so prepared from all the sampled PSU served as the sampling frame for the selection of households in the next stage. From the prepared list, 25 households per PSU were sampled using equal systematic random sampling after determining the sampling interval by dividing

the number of listed household by 25 and by randomly selecting the starting number between 0 and the sampling interval."
Age range of participants included: 15 to 69 years
Source: STEPS Survey Nepal 2019 Report. Available at: https://extranet.who.int/ncdsmicrodata/index.php/catalog/771/related_materials

Rwanda: STEPS 2012-2013

Participants were Rwandan residents aged 15-64 years. Because it was not feasible to conduct a census on the whole population, a representative random sample of participants was selected. To detect statistically significant differences between categories, the WHO STEPwise methodology suggests a minimum sample of 384 people for every age, sex rural/urban or province category the results will be stratified by. For the Rwandan survey the MOH was interested in looking at both males and females across five age groups (15-24 years, 25-34 years, 35-44 years, 45-54 years and 55-64 years), yielding a minimum required sample size of 3840. This was multiplied by 1.5 to account conservatively for the likelihood of a selected participant having the risk factor of interest and then divided by 0.80 assuming that only 80% of those invited to participate would actually participate. This yielded a required sample size of 7200 participants.
Multistage cluster sampling was used to select these participants from the population based on information from the last census. The three levels of clustering were: 1. Random selection of a statistical enumeration area (as defined by NISR) 2. Random selection of a household within the enumeration area 3. Random selection of an individual within the household.
Administratively, Rwanda is divided into thirty districts. In turn, each district is subdivided into sectors. Each sector is sub-divided into cells and then into villages. Villages are synonymous with enumeration area's (EAs) in Rwanda and there are a total of 14,953 EAs in Rwanda. A total of 180 EA's (or 1.2%) were randomly selected from this total using a probability proportional to size method that gives those EA's with more people living in them a higher chance of being selected. In this way, the representativeness of the selected EAs is maximized.
Age range of participants included: 15-64 years
Source: Republic of Rwanda Non-communicable Diseases Risk Factors Report 2012. Available at: https://extranet.who.int/ncdsmicrodata/index.php/catalog/709

Samoa: STEPS 2013

The STEPS survey of chronic disease risk factors in Samoa was carried out from April 2013 to May 2013. Samoa carried out Step 1, Step 2, and Step 3. Socio demographic and behavioural information was collected in Step 1. Physical measurements such as height, weight and blood pressure were collected in Step 2. Biochemical measurements were collected to assess blood glucose and cholesterol levels in Step 3. The STEPS survey was a population-based survey of adults aged 18-64. A multi-stage, cluster sample design was used to produce representative data for that age range in Samoa. A total of 1766 adults participated in the survey. The overall response rate was 64%.
Age range of participants included: 18 to 64 years
Source: Samoa STEPS Survey 2013 Fact Sheet. Available at: https://extranet.who.int/ncdsmicrodata/index.php/catalog/707

Seychelles: STEPS 2013

"The survey was performed in a sex and age stratified random sample of all adults aged 25-64 years of Seychelles between October and December 2013 on Mahé and during 2 weeks in February 2014 in the islands of Praslin and La Digue. These three islands account for >98% of the total population of Seychelles. The eligible sample was extracted from the population registry. The survey was attended by 1240 adults, with a participation rate of 73%. Participants were invited to attend the survey on selected days in study centers located in Mahé, Praslin, and La Digue. All the eligible participants who did not attend were actively traced using (telephone, local administration, announcements on radio, etc) and invited to attend the survey. Since participants were randomly selected from the general adult population, findings of the survey can be inferred to the general adult population of Seychelles."
Age of participants included: 25-64 years

Source: National Survey of Noncommunicable Diseases in Seychelles 2013-2014 (Seychelles Heart Study IV): methods and main findings. Available at: http://www.who.int/chp/steps/seychelles/en/.

Additional reference: Bovet P, Romain S, Shamlaye C, Mendis S, Darioli R, Riesen W, et al. Divergent fifteen-year trends in traditional and cardiometabolic risk factors of cardiovascular diseases in the Seychelles. Cardiovasc Diabetol. 2009; 8:34. https://doi.org/10.1186/1475-2840-8-34 PMID: 19558646

Solomon Islands: STEPS 2015

A multi-stage cluster sample design was used to produce representative data. Analysis weights were calculated by taking the inverse of the probability of selection of each participant. These weights were adjusted for differences in the age-sex composition of the sample population as compared to the target population.

Different weight variables are available per Step:
wStep1 - for interview data
wStep2 - for physical measures
wStep3 - for biochemical measures
This allows for differences in the weight calculation for each Step of the survey as the age-sex composition of the respondents to each Step can differ slightly due to refusal or drop out."
<u>Age range of participants included:</u> 18 to 69 years
Source: no report or fact sheet available. Sampling information obtained from: https://extranet.who.int/ncdsmicrodata/index.php/catalog/710/study-description#page=overview&tab=study-desc

Sri Lanka: STEPS 2014

A multi stage cluster sampling method was used to select a nationally representative sample from the total population. Department of Census and Statistics of Sri Lanka performed the selection of the study sample. Population of each divisional secretariat (DS) divisions as per the preliminary results of the Census done in 2012 was used for sampling.Sri Lanka is administratively divided in to 9 provinces and 25 districts. Each district is divided to Divisional Secretariat (DS) areas. Each DS area is divided to many Census Blocks, and each Census Block consists of many households. The primary sampling unit (PSU) was a Divisional Secretariat (DS) area. Out of 331 DS areas available, 80 DS divisions were selected using proportionate to the size (PPS) sampling. A census block was considered as a SSU. From each DS division (PSU), six secondary sampling units (SSU) were selected using the proportionate to the size (PPS) sampling technique. Therefore, a total of 480 SSUs or census blocks were selected from 80 PSUs. Number of houses in each census block depends on the area density and the population density in each DS division. Tertiary sampling unit (TSU) was the household and 15 households from each CB by random systematic sampling by the Department Census and Statistics. Therefore, a sample of 7200 (80x6x15) households were selected. In some instances, there were more than one household living in one house. People who are cooking and eating together were considered as one household. Whenever there were more than one household in a house, one household was selected randomly to be included in the study.
Only one participant from each household was included in the survey. All the eligible members in the selected family were listed in descending order according to the age. Once this was done, these data was fed to the personal digital assistants (PDAs). The PDAs then automatically selected the eligible participant using the Kish method.
<u>Age range of participants included</u>: 18 to 69 years
Source: Sri Lanka STEPS 2014 Report. Available at: https://extranet.who.int/ncdsmicrodata/index.php/catalog/614/study-description#page=overview&tab=study-desc

Sudan: STEPS 2016

A four-stage cluster sampling design was implemented. The four sampling stages were; 1) selection of states from the six regions 2) selection of clusters (a cluster was a Popular Administrative unit), 3) selection of households and 4) selection of eligible individuals. First Stage (State): Administratively Sudan is divided into 18 states which are grouped in six regions, (North, East, Khartoum, Central, Kordofan and Darfur region (Table 1). States were randomly selected from each region. No geographical areas or populations were excluded from the sampling frame. Thus 11 states were selected, probability proportional to the size, to represent the six regions. A list of the selected states is shown in Table 2.1. Second Stage (Cluster PAU): The Popular Administrative Units (PAU) is the smallest geographically border unit. These were defined as the 'cluster' in the region. Clusters were randomly sampled from all PAUs, from both urban and rural strata, according to probability proportional to size in each state, and urban/rural distribution. The PAUs inaccessible due to security conditions were not excluded from the sampling frame, because within certain areas the security status was continuously changing. However, it was planned that if a PAU was found to be inaccessible at survey time, it should be replaced. However, no replacement was required during this survey. Third Stage (Household): Within the selected PAUs, all households (HH) were included in the sampling frame. Accordingly (HH) were selected using systematic random methods.
Fourth Stage (Individual): The members of the household were first listed in the mobile application (customized software). The inclusion criteria for the listed members were: all individuals aged between 18 to 69 years, from both sexes, irrespective of his health status and living in the selected household for a minimum of 6 weeks. The application was then run and it randomly selected the individual who will be selected to participate in the study.
Age of participants included: 18-69 years.
Source: Sudan STEPS 2016 report. Available at:
https://www.who.int/ncds/surveillance/steps/Sudan_STEPwise_SURVEY_final_2016.pdf?ua=1

Tanzania: STEPS 2012

"The STEPS survey in the United Republic of Tanzania was a population-based survey of adults aged 25-64. The study used both multistage cluster and random probability sampling procedures. Fifty of 119 total districts were randomly selected as primary sampling units (PSUs). Within these PSUs, enumeration areas (EAs) of > 50 households were randomly selected. Any EA with < 50 households was merged with a neighboring EA. Within the EAs, households were randomly selected from a list of all eligible households in the EA. A total of 5762 adults participated in the Tanzania STEPS survey. Within each selected household, the Kish method was used to select the STEPS participant. This procedure was followed until the predetermined sample was obtained for the enumeration area. The response rate for this survey was 94.7%."
Age range of participants included: 25 to 64 years
Source: Tanzania STEPS Survey Report. Available at:
http://www.who.int/chp/steps/UR_Tanzania_2012_STEPS_Report.pdf?ua=1

Additional reference: Mayige M, Kagaruki G. Tanzania STEPS survey report. Dar es Salaam: National Institute of Medical Research; 2013.

Timor-Leste: STEPS 2014

"Note: Data from Census 2010 were used for all sampling considerations. Even though planning and mapping for 2015 Census is ongoing, data from the Census will only be available after July 2015.
STEP 1: Selection of Enumeration Area
(1) List of EA with number of HH by district for Census 2010 was obtained from the Directorate of Statistics. There are 1826 EAs in Timor-Leste. Out of these, 150 EAs were selected.
(2) The number of EAs to be selected from each district was based on their proportion in the country's population as per Census 2010.
(3) The numbers of Households (HH) per EAs varied from 0 to more than 300. Therefore, probability proportion to size (PPS) was used.
(4) For each district, the EAs were arranged in ascending order of HH size.

(5) Sampling interval was obtained by dividing the total number of HH in the district by the number of EA to be selected from that district.
(6) A random number was generated between one and the sampling interval for that district, using tools available at random.org.
(7) The EA where that random number fell was the first EA to be selected.
(8) Subsequently, the sampling interval was added to the random number and the EA where this new number fell was selected. For the next number, the sampling interval was added to the number and so on, till the population of HH was exhausted or target number of EA achieved.
(9) This was done separately for each district.
(10) The final list was compiled and had 150 EAs. These are spread over about 125 sucos.
STEP 2. Selection of Households in an Enumeration Area
Listing the house numbers to be visited
(1) It was decided to use the 2010 HH size of each EA. Based on past experience, it was expected that the increase would be on an average about 4–5%.
(2) The list of households to be selected by enumerators was decided centrally.
(3) Sampling interval was calculated by dividing the total number of households in the EA by 18.
(4) The first HH number was selected randomly by reading the last two digits of a currency note. If the number represented by the two digits was more than 18, the last digit was taken into consideration. For each EA, a different currency note was used. This could also be done it by using the tool at random.org. or by draw of lots.
(5) The subsequent HH are identified by adding the sampling interval as was done for selection of EA."
Age range of participants included: 18 to 69 years
Source: Timor-Leste STEPS Survey Report, [online] at http://www.who.int/entity/chp/steps/Timor-Leste_2014_STEPS_Report.pdf?ua=1

Togo: STEPS 2010
"Those included in this survey are male or female subjects, living in urban or rural areas, aged 15 to 64 on the day of the survey, residing in the enumeration area for at least 6 months and having given their informed consent to participate in this study. [...] Three hundred clusters were randomly selected in a systematic draw with probability proportional to the size of the cluster (number of households) in the 4620 areas of enumeration of the DGSCN (General Directorate of Statistics and National Accounts) sampling frame. In order to obtain the 4,800 households at the rate of 1 individual / household, 16 households per cluster were randomly selected at the second stage of survey. In each of the selected households, one individual was selected as a survey participant via the Kish Method. A household was defined as the group of persons, who regularly share the main meal (regardless of their relationship). Households were not replaced in the event of a refusal or two unsuccessful visits to the eligible person selected by Kish's method. If the selected person was unwell or not present at the time of the interview, the investigators either tried to find a new appointment or searched for the respondent."
Age range of participants included: 15 to 64 years
Source: Translated from WHO: The Final Report on the Togo STEPS Survey 2010. Available at: http://www.who.int/chp/steps/2010STEPS_Report_Togo_FR.pdf?ua=1.

Tokelau: STEPS 2014
A whole population-based (census) survey was used to produce representative data for that age range in Tokelau. Analysis weights contain adjustments for differences in the age-sex composition of the sample population as compared to the target population. Different weight variables are available per Step:
wStep1 - for interview data
wStep2 - for physical measures
wStep3 - for biochemical measures
This allows for differences in the weight calculation for each Step of the survey as the age-sex composition of the respondents to each Step can differ slightly due to refusal or drop out.

Source: Report unavailable. Sampling information obtained from: https://extranet.who.int/ncdsmicrodata/index.php/catalog/638/overview#page=sampling&tab= study-desc

Tuvalu: STEPS 2015

"The Tuvalu STEPS Survey was a population based cross-sectional survey of 18-69 year olds. Analysis weights were calculated by taking the inverse of the probability of selection of each participant. These weights were adjusted for differences in the age-sex composition of the sample population as compared to the target population.

Different weight variables are available per Step:
wStep1 - for interview data
wStep2 - for physical measures
wStep3 - for biochemical measures
This allows for differences in the weight calculation for each Step of the survey as the age-sex composition of the respondents to each Step can differ slightly due to refusal or drop out. Additionally, some countries perform subsampling for Step 2 and/or Step 3. When no subsampling is done and response rates do not differ across Steps of the survey, the 3 weight variables will be the same."
<u>Age range of participants included:</u> 18 to 69 years
Source: no report or fact sheet available. Sampling information obtained from: https://extranet.who.int/ncdsmicrodata/index.php/catalog/639/study-description#page=overview&tab=study-desc

Uganda: STEPS 2014

"Uganda has a total population of 34.9 million people, approximately 43% of which are adults aged 18 years or older [14]. The survey covered the whole country, and a three stage sampling design was used to select participants. The sampling procedure utilized the Uganda Bureau of Statistics (UBOS) master sampling frame of Enumeration Areas (EAs) that had just been demarcated throughout the country in preparation for the 2014 population and housing census. Each EA included 150–200 households. In the first stage, a random sample of 350 out of 78,950 EAs was selected with selection probability proportional to the size (PPS) of the number of households in the EAs. The EAs were stratified across the four regions of Uganda namely: Central, Eastern, Northern and Western region; and were selected with separate estimates for rural and urban areas. Urban areas were defined as EAs within government designated urban areas, or those within other geographic divisions with population density of more than 1000 per square kilometer.

After selecting the 350 EAs, trained teams of UBOS staff were dispatched throughout the country to list the households and their household heads within the 350 EAs. A household was defined as a group of individuals that usually shared meals together, and had a household head who usually made major decisions for the household. In the second stage of sampling, 14 households were randomly selected from the listed households in each of the sampled EAs.

Research Assistants (RA) that had received a five-day training on procedures and administration of the STEPs tool, enumerated eligible household members who were recorded in Personal Digital Assistants (PDA), which was then used to randomly select one subject for inclusion in the survey giving a total sample of 4900. Eligible subjects were household members aged 18 to 69 years, who had resided in the sampled households for at least six months preceding the date of interview."
<u>Age range of participants included</u>: 18 to 69 years
Source: Guwatudde D, Mutungi G, Wesonga R, Kajjura R, Kasule H, Muwonge J, et al. (2015) The Epidemiology of Hypertension in Uganda: Findings from the National Non-Communicable Diseases Risk Factor Survey. PLoS ONE 10(9): e0138991. doi:10.1371/journal.pone.0138991.

Additional reference: Bahendeka S, Wesonga R, Mutungi G, Muwonge J, Neema S, Guwatudde D. Prevalence and correlates of diabetes mellitus in Uganda: a population-based national survey. Trop Med Int Health. 2016; 21 (3):405–16. https://doi.org/10.1111/tmi.12663 PMID: 26729021

Vanuatu: STEPS 2011

"The survey used a cluster sampling design where the primary sampling unit was enumeration area (EA) and the secondary sampling unit was households. All 6 provinces in Vanuatu were included in the survey. One hundred and thirteen (113) EAs were randomly selected proportion to the size of the EA from a total of 411 EAs. Forty four (44) households were then randomly selected in each EA proportional to the number of households in each EA. The selection of participants within each household was done using the Kish method. The total number of households selected by combined Enrolment Areas was 4,972.

The required sample size was calculated as 4972 households on a margin of error of 0.05, an anticipated response rate of 89% and with 80% power to detect statistically significant differences between six age/sex groups. Accordingly, from the 4,972 selected households 4,649 individuals aged 25-64 years participated in STEP 1 and STEP 2 giving an overall response rate of 94%. The response rate dropped to 85% for STEP 3 with 4,224 people participating."

Age range of participants included: 25 to 64 years

Source: Vanuatu STEPS report [online]: https://extranet.who.int/ncdsmicrodata/index.php/catalog/714

Vietnam: STEPS 2015

At the same time of STEP survey, MOH also conduct the Global Adult Tobacco Survey (GATS) at the same scale, location, and study subjects (>15 years for GATS and 18-69 for STEPS). The sampling of STEPS was done in as part of the sampling for the (GATS) conducted in combination manner to save time and resources for these two surveys. Applied the multi-stages complex sampling process, the sampling process done by GSO was as follow: • Sampling of clusters (EA) In the first stage of sampling, the primary sampling unit (PSU) was an enumeration area (EA). There are about 170,000 EAs in the whole Viet Nam and the average number of households in each EA is different between urban and rural areas. An average number of households in an urban EA and a rural EA is 133 households and 120 households, respectively. Sample of EAs were selected from the master sample frame. The master sample frame was a cluster frame made by the GSO based on the frame of Population and Housing Census 2009 and updated with data of 2014. Based on the Population and Housing Census data 2009, GSO prepared a 15% of master sample to serve as a national survey sampling frame. The master sample frame contains 25,500 enumeration areas (EAs) from 706/708 districts of Viet Nam (2 island districts were excluded from the GSO master sample frame). The master sample frame of GSO was divided by two stratification variables: urbanization (1 = urban; 2 = rural) and district group (1 = district/town/city of province; 2 = plain and coastal district; 3 = mountainous, island district). It means that the master sample frame was divided into 6 sample frames or 6 strata. The probability proportional to size (PPS) sampling method was used to select sample of EAs from 6 strata of master sample frame. The final sample of GATS included 315 EAs in the urban and 342 EAs for the rural. From these 657 EAs, 315 EAs were systematically selected for STEPS. Sampling of households At the second stage of sampling, 10% households in each EA were selected. Thus, 15 households from the selected urban EA and 14 households from the selected rural EA were chosen using simple systematic random sampling. The total households for STEPS 2015 were 4,651 households.

Sampling of individuals: One eligible person is then randomly selected from each selected household for the STEPS 1 interview. The selection of individual is automatically done by the PDA program after eligible household members are entered into the PDA. The selection probability of an eligible individual was calculated as a product of selection probability for each stage. The sampling base weight for an eligible individual was the inverse of the selection probability shown above.

Age range of participants included: 18 to 69 years

Source: National Survey on the Risk Factors of Non-communicable diseases (STEPS) Viet Nam Report 2015. Available at: https://www.who.int/ncds/surveillance/steps/viet_nam/en/

Zambia: STEPS 2017
To ensure that the sample reflected the entire country of Zambia, a multi-stage cluster sampling technique was used to select a nationally representative sample of adults in Zambia aged 18 to 69 years. It was decided to utilize the household listing from the Zambia PopulationBased HIV Impact Assessment (ZAMPHIA) - a household-based national survey that was conducted between March and August 2016 in order to measure the status of Zambia's national HIV response. ZAMPHIA offered the most pragmatic up to date and accessible national household listing to be used as the sampling frame for this survey. The ZAMPHIA survey included 60,581 households drawn from 1,103 clusters referred to in this report as standard enumeration area (SEA) (Table 2.4.1). Thus the sample drawn for the STEPS survey was a subsample of the households selected for the ZAMPHIA survey. In the first stage of sampling, SEAs were selected from each province using probability proportional to size (PPS). In the second stage, 15 households in rural SEAs and 20 households in urban SEAs were selected systematically using appropriate sampling interval based on the number of households in that SEA. These households constituted the final list of households for the STEPS survey prepared for the field investigators (FI). In the third stage, while the FI approached the household and sought consent, all eligible members in the household were entered into the Android-based devise used for the survey. The device then selected one member from the eligible members using a simple random sampling technique. The selected member was then interviewed having gone through the ethical process of consent after being provided with information on the survey. If the selected member was not available, a scheduled visit was made. If the selected member could not be reached after two scheduled visits he or she was considered as non-response. There was no replacement strategy so as to maintain the integrity and representativeness of the sample.
Age range of participants included: 18 to 69 years
Source: STEPS 2017 Report. Available at:
https://extranet.who.int/ncdsmicrodata/index.php/catalog/620

Zanzibar STEPS 2011
"The survey took place in June and July 2011, followed by data cleaning and analysis. One Principal Investigator and five assistant researchers coordinated the survey on site, checked completed questionnaires daily, and organized logistics. The six data collection teams consisted each of six interviewers, one supervisor, one laboratory technician and one driver. Interviewers were either health care workers or professional interviewers familiar with household surveys such as DHS. The sample size was calculated to be 2800 participants. Each interviewer did on average 3 – 4 interviews a day and was assisted on site by local village guides.

The study was a cross-sectional population based survey with a sample of a sufficient size with a power to determine the proportion of adults that are exposed to selected risk factors associated with NCDs; including those having raised BP, FBG or blood lipids, had experienced injuries or traumas in recent times, and/or were mentally unwell (anxiety, depression), as well as linking these conditions with one another and with the sociodemographic and economic information obtained. People reported to be permanent residents (spending on average maximum 3 nights per week outside the house, and not holding an address in another place) in the selected households and fulfilled the inclusion criteria were enrolled into the survey. A person could only appear once in the study. Therefore we classified a husband practicing polygamy to be listed in the household of his first wife but not to be a member in the household of the following wives. Inclusion criteria was age between 25 - 64 years, able to understand the information given by the interviewer about the study prior to the beginning of the interview, signing of the informed consent for accepting participation. Exclusion criteria was inability to understand or comprehend the information given by data collector, inability to communicate through verbal expression for consent and for responding to the questionnaires, severe/terminal illness that hinders participation in the survey.

The target population is the entire population in Zanzibar whereby the whole of Zanzibar was selected as the survey site, and hence all districts included. The total population is estimated to be 1.2 million distributed unevenly between 10 districts. The sampling frame represented the entire population in Zanzibar. The sampling strategy used is a multi-stage cluster sampling with stratification. The ten districts are considered as different strata, and the total number of primary sampling units, PSU, is allocated proportionately across all strata. Each district is divided into smaller clusters. These clusters are the geographical and administrative units called Shehia11. The Shehia are divided into smaller clusters called zones (also called mitaa, vitongoji, or vijiji) which typically consist of 100-300 households. Zones smaller than that were merged to make up one larger cluster, and zones much larger were split in smaller clusters.

At the first stage clusters were selected using Simple Random Selection, SRS, from the list of clusters (Shehia) within each district. At the second stage clusters (zones) were randomly selected using probability proportionate to size (PPS). At the third stage households were randomly selected from the household lists provided by the administrative leader of the Shehia. The two last stages of sampling were done using the software STEPSsampling.xls from WHO. Finally participants were selected from the household using Kish method. The household lists were complete and included households with no eligible participants for the survey. Therefore an extra 7 households were sampled at third stage in each cluster for replacement in case a selected household had no eligible participants and had to be changed. This was done before data collectors went to the cluster.

Resources allowed for 100 PSU which was why 2800/100 = 28 households were selected from each PSU (and disproportionate from each SSU). A structured questionnaire was used, based on WHO STEPwise approach to chronic diseases risk factor surveillance.. After getting behavioural and socio-demographic information, anthropometric measurements (BP, height, weight, waist and hip circumference) was done the same day. Answers were recorded electronically during interview using a Personal Digital Assistant (PDA). Biochemical measurements (fasting blood glucose, triglyceride, and cholesterol levels) were done the next day at a central place in each study site according to appointment and were done by Laboratory technicians using dry chemistry for rapid and convenient results and to avoid suspicion surrounding sending away blood samples. Results were recorded electronically on site using a PDA, and participants received a paper copy of the results.

Every study site was visited one day for interviews. Sampled households/ participants were visited at least three times before recorded as non-respondent. The following day the site was visited for biochemical measurements. Laboratory technicians called participants who did not show up to ask them to set up appointment for the following day (at a new study site). After all study sites had been visited call-backs were made to all eligible participants (non-respondents) who's number we had obtained. A time and place near the participants was identified for data collection. Participants met fasting and started with having blood sample drawn, afterwards the interviews and anthropometric measurements were conducted. Laboratory technicians continued biochemistry measurements for another few days.
Age range of participants included: 25 to 69 years
Source: Zanzibar STEPS Survey Report, [online]
https://www.who.int/ncds/surveillance/steps/2011_Zanzibar_STEPS_Report.pdf

Appendix 5: Detailed methodology for diabetes biomarkers by country

Diabetes Biomarker	Country	Post Hoc Adjustment*
Point-of-care fasting capillary glucose		
Accu-check	Samoa, Tuvalu	None
Accutrend® Plus (Roche, Basel, Switzerland)	Cambodia, Chile, Liberia, Togo, Zanzibar	Multiplied by 1.11
CardioCheck® PA (pts Diagnostics, Indianapolis, Indiana, USA)	Belarus, Benin, Bhutan, Burkina Faso, Eswatini, Kenya, Moldova, Morocco, Nepal, Rwanda, Solomon Islands, Sri Lanka, Timor-Leste, Sudan, Uganda, Vietnam, Zambia	None
CONTOUR® (Ascensia Diabetes Care Holdings AG, Basel, Switzerland)	Seychelles	None
HemoCue® Glucose 201 Analyzer (HemoCue, Brea, California, USA)	Namibia, Tanzania	None
MultiCare-in© (Biochemical Systems International, Arezzo, Italy)	Georgia	None
SD LipidoCare Analyzer (automatic plasma equivalent)	Myanmar	None
Unknown	Algeria, Azerbaijan, Botswana, Ecuador, Eritrea, Kyrgyzstan, Laos, Lesotho, Marshall Islands, Tokelau,Vanuatu	None
Laboratory-based Assessment of Fasting Plasma Glucose		
Auto analyzer Selectrao Pro M Human ®, Germany	Bangladesh	N/A
Central laboratory was used for processing	Lebanon	N/A
Cobas 6000 and C311 analyzer (Roche Diagnostics, Indianapolis, Indiana, USA)	Iran	N/A
Enzymatic assay (glucose oxidase)	Iraq	N/A
SYNCHRON® System (Beckman Coulter, Inc., Miami, Florida, USA)	Costa Rica	N/A
Hemoglobin A1c (HbA1c)		N/A
Point-of-care Bayer Consumer Care AG (A1cNow)	Seychelles	
Plasma sample by Cobas C311 auto-analyzer (Roche kits)	Iran	N/A

*Post hoc adjustment to convert from capillary to plasma equivalents

Appendix 6: Fasting requirements per survey

Survey type	Fasting instructions
STEPS	12 hours with nothing to eat or drink, other than water
DHS	8 hours or more of fasting from the time the participant woke up until when they underwent glucose testing. Specific questions: • When was the last time you had something to eat? • When was the last time you had something to drink other than plain water?
Chile: NHS 2009-10	8 hours or more with nothing to eat or drink, other than water

Appendix 7: Detailed methodology for household wealth index calculation

Across surveys, several different wealth indicators were measured including continuous income, income categories, income quintiles, an asset index, or a combination of these (see table below). In an effort to homogenize wealth in the pooled analysis, we constructed household wealth quintiles for each survey.

Wealth Measure	Country
Asset index	Bangladesh, Kenya, Namibia, Iran
Continuous income	Bhutan, Eritrea, Laos, Myanmar, Timor-Leste
Continuous income and quintiles	Algeria, Azerbaijan, Benin, Botswana, Cambodia, Ecuador, Eswatini*, Georgia, Kyrgyzstan, Lesotho, Liberia*, Moldova, Rwanda*, Samoa*, Solomon Islands, Tanzania, Togo*, Uganda*, Vanuatu, Zambia, Zanzibar
Continuous income and categories	Benin, Lebanon, Marshall Islands, Morocco
Income categories only	Sudan
No wealth indicators assessed	Belarus, Burkina Faso, Chile, Costa Rica, Iraq, Nepal, Vietnam

*Quintiles were not used as they displayed large discrepancies with respect to continuous income range or could not be correctly identified

The construction of wealth quintiles depends on the given wealth indicator. Countries using an asset index surveyed a range of assets, dwelling characteristics, and further country-specific variables. Utilizing the standard DHS approach, we used principle component analysis to derive an asset index, from which we create unweighted wealth quintiles. Countries using an income-based measurement mainly followed the STEPS template questionnaire put forward by the WHO. In this, respondents were asked about the average earnings (taking the past year) of the household in a week, month, or year. In cases where this question was left unanswered, a pre-coded estimate of the households' annual income was indicated. This pre-coded estimate was usually expressed as quintiles and sometimes as categories that were defined by the countries' survey teams. Using both the pre-coded estimates as well as the continuous income, we again created unweighted wealth quintiles. In this, we assumed that national incomes follow a log-normal distribution and made use of the procedure put forward by Harttgen and Vollmer (2013) in combining income quintiles and categories. In seven cases, we dismissed pre-coded quintiles or income as they displayed very large discrepancies with respect to the continuous income range or could otherwise not be correctly identified. However, as the pre-coded estimates were typically only asked of respondents that had not indicated a continuous income, this led to only minor information losses.

Reference:

Harttgen, K., & Vollmer, S. (2013). Using an asset index to simulate household income. Economics Letters, 121(2), 257-

Appendix 8. List of national, regional, or sub-regional programs with a diabetes prevention component available in 39 of the 44 LMICs included in this study

Country	National, regional, or sub-regional programs with a diabetes prevention component	Project period
Azerbaijan	Establishment of diabetes schools (WDF10-535)[1]	2011-2013
Bangladesh	Primary prevention of diabetes (WDF06-193)[2]; Diabetes Prevention through Religious Leaders in Bangladesh (WDF17-1552)[3]	2007-2011; 2018-2020
Belarus	Preventing Non-Communicable Diseases, Promoting Health Lifestyle and Support to Modernization of the Health System in Belarus (BELMED)[4]	2015-2020
Benin	Prevention of diabetes in Benin (WDF14-872)[5]	2015-2019
Bhutan	Diabetes healthcare services (WDF03-060)[6]; Diabetes care services (WDF10-493)[7]	2004-2010; 2010-2014
Botswana	Improving diabetes care for diabetes and foot complications (WDF08-394)[8]; Botswana Multi-Sectoral Strategy for the Prevention and Control of Non-Communicable Diseases[9]	2010-2012; 2018-2023
Burkina Faso	Strengthening diabetes care (WDF09-424)[10]; Improving diabetes prevention and care in Burkina Faso (WDF12-753)[11]; Strengthen diabetes care and prevention in Burkina Faso (WDF1901734)[12]	2009; 2014; 2020-2023
Cambodia	Provincial diabetes peer educator network (WDF09-463)[13]; Coordinated DM-TB Services in Cambodia (WDF14-852)[14]; Cambodia project for early detection and management of diabetes in primary care (WDF17-1504)[15]	2009-2011; 2014-2016; 2019-2022
Chile	Vida Sana[16]	Ongoing
Costa Rica	Estrategia Nacional Abordaje Integral de las Enfermedades Cronicas no Transmisibles y Obesidad 2013-2012[17]	2013-2021
Ecuador	Salud al Paso[18]	2015-2019
Eritrea	Reduction of the diabetes burden (WDF06-194)[19]	2007-2011
Eswatini	Integrating diabetes screening into HIV services in Swaziland (WDF17-1467)[20]	2017-2019
Georgia	Diabetes prevention and care improvement (WDF12-720)[21]; Diabetes prevention in rural Georgia (WDF13-800)[22]; Improved control of diabetes in remote areas in Kvemo Kartli region (WDF17-1471)[23]	2012-2014; 2014-2016; 2018-2021
Iran	National Diabetes Prevention and Control Program[24]; Diabetes clinics (WDF07-282)[25]	1999-; 2008-2011
Iraq	The National Strategy for Prevention and Control of Noncommunicable diseases[26]	2013-2017
Kenya	Kenya National Diabetes/NCD Response Programme support (phase 1: WDF09-436/phase 2: WDF16-1344)[27]; Promoting health living in schools (WDF13-807)[28]; Action for diabetes in Kenya (WDF15-1267)[29]	2009-2015; 2014-2018; 2017-2020
Kyrgyzstan	Improving diabetes prevention and care (WDF09-466)[30]; Enhancing Diabetes Prevention and Self-Management (WDF14-899)[31]	2010-2013; 2015-2017
Lebanon	Managing diabetes for older people (WDF16-1431)[32]	2017-2019
Lesotho	Improving community and facility-based diabetes care in rural Lesotho (WDF20-1778)[33]	2020-2024
Liberia	Improving access to diabetes care (WDF09-483)[34]; Community based diabetes care (WDF15-1273)[35]; Prevention and care of diabetes in Liberia (WDF17-1488)[36]	2019-2012; 2016-2018; 2018-2020
Marshall Islands	Education in lifestyle diseases (WDF10-524)[37]; Majuro Youth Lifetime Program (WDF16-1418)[38]	2011-2014; 2017-2020
Moldova	Diabetes Help Network- Integrated Care (WDF-F12-028)[39]; Diabetes Schools Network for Strengthening Efforts in Prevention (WDF16-1412)[40]	2013-2015; 2017-2019
Myanmar	Expanding access to primary health care for NCDs in Myanmar (WDF15-1286)[41]	2017-2019
Nepal	Improving access to diabetes care in Nepal (WDF15-1215)[42]; School Based Diabetes Prevention (WDF16-1432)[43]; Diabetes prevention and management by lay health workers in Nepal (WDF16-1441)[44]; Nurse-led continuum of care approach for addressing diabetes in Nepal (WDF17-1483)[45]	2016-2018; 2017-2019; 2017-2021; 2018-2021
Rwanda	National Diabetes Prevention and Control Program (WDF15-1232)[46]	2017-2020

Samoa	1Touch MoH transformation program to tackle diabetes and NCDs in Samoa (WDF17-1465)[47]	2017-2019
Seychelles	WHO Package of Essential Non-communicable disease interventions[48]	2019-
Solomon Islands	Improving diabetes care (WDF08-396)[49]	2010-2012
Sri Lanka	General diabetes care (WDF09-411)[50]; Sri Lanka national NCD response programme support (WDF12-683)[51]; Sri Lanka Diabetes and CVD initiative (WDF15-1291)[52]	2009-2012; 2012-2015; 2016-2017
Tanzania	Diabetes awareness, care and referral in Lake Zone- a comprehensive model of care (WDF07-265)[53]; Community and school outreach (WDF09-450)[54]; Tanzania National NCD Response Programme support (WDF06-221)[55]; National Diabetes Primary Prevention Program (WDF13-719)[56]	2017-2011; 2009-2012; 2012-2018; 2014-2018
Timor-Leste	NCD National Action Plan[57]	2014-2018
Togo	Training of diabetes awareness educators (WDF07-237)[58]; Improvement in care of diabetes in Togo through decentralization of care (WDF14-917)[59]; Diabetes and HIV/AIDS prevention and care in Togo (WDF17-1537)[60]	2008-2011; 2015; 2018-2021
Tuvalu	Tuvalu National Strategic Plan for Non Communicable Diseases 2011-2015[61]	2011-2015
Uganda	National NCD Programme (WDF06-222)[62]; Diabetes Prevention through School Programme (WDF13-819)[63]; Optimising Care through Integrating NCD Services in Primary Care (WDF19-1721)[64]	2008; 2015-2018; 2020-2022
Vanuatu	The 10,000 Toes Campaign[65]	2020-
Vietnam	Abundant Health ; Communities for Healthy Vietnam[66]	2016-2020; 2020-
Zambia	Diabetes Awareness and Treatment Programme (WDF14-933)[67]	2015-2018
Zanzibar	NCD Support Programme Zanzibar (WDF11-593)[68]	2012-2019

This list was compiled following a systematic google search using the advanced function with the terms "diabetes prevention program" and no language restrictions in each of the 44 countries included in this study. We reviewed the first 30 hits. Given that the World Diabetes Foundation has sponsored the majority of diabetes prevention programs in LMICs, we additionally searched for projects listed on the World Diabetes Foundation website[69] using their search engine. This search did not identify diabetes prevention programs in Algeria, Laos, Morocco, Namibia, and Tokelau, though it is possible that such programs exist and may be identified through other search engines.

References:

1. Establishment of diabetes schools WDF10-535 [Internet]. World diabetes foundation. 2012 [cited 2022 Aug 18]. Available from: https://www.worlddiabetesfoundation.org/projects/azerbaijan-wdf10-535
2. Primary prevention of diabetes WDF06-193 [Internet]. World diabetes foundation. 2012 [cited 2022 Aug 17]. Available from: https://www.worlddiabetesfoundation.org/projects/bangladesh-wdf06-193
3. Diabetes Prevention through Religious Leaders in Bangladesh, WDF17-1552 [Internet]. World diabetes foundation. 2018 [cited 2022 Aug 18]. Available from: https://www.worlddiabetesfoundation.org/projects/bangladesh-wdf17-1552
4. Preventing Non-communicable Diseases, Promoting Healthy Lifestyle and Support to Modernization of the Health System in Belarus (BELMED) | United Nations Development Programme [Internet]. UNDP. [cited 2022 Aug 18]. Available from: https://www.undp.org/belarus/projects/preventing-non-communicable-diseases-promoting-healthy-lifestyle-and-support-modernization-health-system-belarus-belmed
5. Prevention of Diabetes in Benin WDF14-872 [Internet]. World diabetes foundation. 2015 [cited 2022 Aug 17]. Available from: https://www.worlddiabetesfoundation.org/projects/benin-wdf14-872
6. Diabetes care services WDF10-493 [Internet]. World diabetes foundation. 2013 [cited 2022 Aug 17]. Available from: https://www.worlddiabetesfoundation.org/projects/bhutan-wdf10-493
7. Diabetes care services WDF10-493 [Internet]. World diabetes foundation. 2013 [cited 2022 Aug 18]. Available from: https://www.worlddiabetesfoundation.org/projects/bhutan-wdf10-493
8. Improving diabetes care for diabetes and foot complications WDF08-394 [Internet]. World diabetes foundation. 2012 [cited 2022 Aug 18]. Available from: https://www.worlddiabetesfoundation.org/projects/botswana-wdf08-394
9. Policy - Botswana Multi-Sectoral Strategy for the Prevention and Control of Non-Communicable Diseases | Global database on the Implementation of Nutrition Action (GINA) [Internet]. [cited 2022 Aug 17]. Available from: https://extranet.who.int/nutrition/gina/en/node/39766
10. Strengthening diabetes care WDF09-424 [Internet]. World diabetes foundation. 2012 [cited 2022 Aug 18]. Available from: https://www.worlddiabetesfoundation.org/projects/burkina-faso-wdf09-424

11. Improving diabetes prevention and care in Burkina Faso WDF12-753 [Internet]. World diabetes foundation. 2014 [cited 2022 Aug 17]. Available from: https://www.worlddiabetesfoundation.org/projects/burkina-faso-wdf12-753
12. Strengthen diabetes care and prevention in Burkina Faso (WDF19-1734) [Internet]. World diabetes foundation. 2020 [cited 2022 Aug 18]. Available from: https://www.worlddiabetesfoundation.org/projects/wdf19-1734
13. Provincial diabetes peer educator network WDF09-463 [Internet]. World diabetes foundation. 2013 [cited 2022 Aug 18]. Available from: https://www.worlddiabetesfoundation.org/projects/cambodia-wdf09-463
14. Coordinated DM-TB Services in Cambodia WDF14-852 [Internet]. World diabetes foundation. 2014 [cited 2022 Aug 18]. Available from: https://www.worlddiabetesfoundation.org/projects/cambodia-wdf14-852
15. Cambodia project for early detection and management of diabetes in primary care, WDF17-1504 [Internet]. World diabetes foundation. 2020 [cited 2022 Aug 17]. Available from: https://www.worlddiabetesfoundation.org/projects/wdf17-1504
16. Vida Sana [Internet]. SSMS. [cited 2022 Aug 18]. Available from: https://ssms.gob.cl/como-me-cuido/programas-de-salud/vida-sana/
17. Prosperi DJ, Pérez-Flores DE, Fernández DF, Legetic DB. ICODER Ministerio de Salud Ministerio de Salud Ministerio de Salud Ministerio de Salud Ministerio de Salud Ministerio de Salud Ministerio de Salud Ministerio de Salud Ministerio MEP Ministerio de Salud CCSS. :106.
18. Sostenibilidad en la prevención de enfermedades crónicas: lecciones del programa Salud al Paso en Ecuador [Internet]. Revista Panamericana de Salud Pública; [cited 2022 Aug 18]. Available from: https://www3.paho.org/journal/es/articulos/sostenibilidad-prevencion-enfermedades-cronicas-lecciones-programa-salud-al-paso-ecuador
19. Reduction of the diabetes burden WDF06-194 [Internet]. World diabetes foundation. 2012 [cited 2022 Aug 17]. Available from: https://www.worlddiabetesfoundation.org/projects/eritrea-wdf06-194
20. Integrating diabetes screening into HIV services in Swaziland, WDF17-1467 [Internet]. World diabetes foundation. 2018 [cited 2022 Aug 18]. Available from: https://www.worlddiabetesfoundation.org/projects/swaziland-wdf17-1467
21. Diabetes prevention and care improvement WDF12-720 [Internet]. World diabetes foundation. 2013 [cited 2022 Aug 18]. Available from: https://www.worlddiabetesfoundation.org/projects/georgia-wdf12-720
22. Diabetes prevention in rural Georgia WDF 13-800 [Internet]. World diabetes foundation. 2014 [cited 2022 Aug 18]. Available from: https://www.worlddiabetesfoundation.org/projects/georgia-wdf13-800
23. Improved control of diabetes in remote areas in Kvemo Kartli region, WDF17-1471 [Internet]. World diabetes foundation. 2018 [cited 2022 Aug 18]. Available from: https://www.worlddiabetesfoundation.org/projects/georgia-wdf17-1471
24. Azizi F, Gouya MM, Vazirian P, Dolatshahi P, Habibian S. The diabetes prevention and control programme of the Islamic Republic of Iran. EMHJ - East Mediterr Health J 9 5-6 1114-1121 2003 [Internet]. 2003 [cited 2022 Aug 17]; Available from: https://apps.who.int/iris/handle/10665/119368
25. Diabetes clinics WDF07-282 [Internet]. World diabetes foundation. 2012 [cited 2022 Aug 18]. Available from: https://www.worlddiabetesfoundation.org/projects/iran-wdf07-282
26. The National Strategy for Prevention and Control of Noncommunicable Diseases [Internet]. [cited 2022 Aug 18]. Available from: https://wedocs.unep.org/bitstream/handle/20.500.11822/8846/-The%20National%20Strategy%20for%20Prevention%20and%20Control%20of%20Noncommunicable%20Diseases-2013The%20National%20Strategy%20for%20Prevention%20and%20Control%20of%20Noncommunicable%20Diseases-Eng%282%29.pdf?sequence=3&isAllowed=y
27. Kenya National Diabetes/NCD Response Programme support (phase 1: WDF09-436/phase 2:WDF16-1344) [Internet]. World diabetes foundation. 2013 [cited 2022 Aug 17]. Available from: https://www.worlddiabetesfoundation.org/projects/kenya-wdf09-436
28. Promoting healthy living in schools WDF13-807 [Internet]. World diabetes foundation. 2014 [cited 2022 Aug 18]. Available from: https://www.worlddiabetesfoundation.org/projects/kenya-wdf13-807
29. Action for diabetes in Kenya, WDF15-1267 [Internet]. World diabetes foundation. 2017 [cited 2022 Aug 18]. Available from: https://www.worlddiabetesfoundation.org/projects/kenya-wdf15-1267
30. Improving diabetes prevention and care WDF09-466 [Internet]. World diabetes foundation. 2013 [cited 2022 Aug 18]. Available from: https://www.worlddiabetesfoundation.org/projects/kyrgyzstan-wdf09-466
31. Enhancing Diabetes Prevention and Self-management, WDF14-899 | World diabetes foundation [Internet]. [cited 2022 Aug 17]. Available from: https://www.worlddiabetesfoundation.org/projects/kyrgyzstan-wdf14-899
32. Managing diabetes for older people, WDF16-1431 | World diabetes foundation [Internet]. [cited 2022 Aug 18]. Available from: https://www.worlddiabetesfoundation.org/projects/lebanon-wdf16-1431

33. Improving community and facility-based diabetes care in rural Lesotho, WDF20-1778 [Internet]. World diabetes foundation. 2021 [cited 2022 Aug 17]. Available from: https://www.worlddiabetesfoundation.org/projects/wdf20-1778
34. Improving access to diabetes care WDF09-483 [Internet]. World diabetes foundation. 2013 [cited 2022 Aug 18]. Available from: https://www.worlddiabetesfoundation.org/projects/liberia-wdf09-483
35. Community based diabetes care, WDF15-1273 [Internet]. World diabetes foundation. 2017 [cited 2022 Aug 18]. Available from: https://www.worlddiabetesfoundation.org/projects/liberia-wdf15-1273
36. Prevention and care of diabetes in Liberia, WDF17-1488 [Internet]. World diabetes foundation. 2018 [cited 2022 Aug 17]. Available from: https://www.worlddiabetesfoundation.org/projects/liberia-wdf17-1488
37. Education in lifestyle diseases WDF10-524 [Internet]. World diabetes foundation. 2012 [cited 2022 Aug 18]. Available from: https://www.worlddiabetesfoundation.org/projects/marshall-islands-wdf10-524
38. Majuro Youth Lifetime Program, WDF16-1418 [Internet]. World diabetes foundation. 2017 [cited 2022 Aug 18]. Available from: https://www.worlddiabetesfoundation.org/projects/marshall-islands-wdf16-1418
39. Diabetes Help Network – Integrated Care WDF-F12-028 [Internet]. World diabetes foundation. 2013 [cited 2022 Aug 18]. Available from: https://www.worlddiabetesfoundation.org/projects/moldova-wdf-f12-028
40. Diabetes Schools Network for Strengthening Efforts in Prevention, WDF16-1412 [Internet]. World diabetes foundation. 2017 [cited 2022 Aug 18]. Available from: https://www.worlddiabetesfoundation.org/projects/moldova-wdf16-1412
41. Expanding access to primary health care for NCDs in Myanmar, WDF15-1286 [Internet]. World diabetes foundation. 2017 [cited 2022 Aug 17]. Available from: https://www.worlddiabetesfoundation.org/projects/myanmar-wdf15-1286
42. Improving access to diabetes care in Nepal, WDF15-1215 [Internet]. World diabetes foundation. 2017 [cited 2022 Aug 18]. Available from: https://www.worlddiabetesfoundation.org/projects/nepal-wdf15-1215
43. School Based Diabetes Prevention, WDF16-1432 [Internet]. World diabetes foundation. 2017 [cited 2022 Aug 18]. Available from: https://www.worlddiabetesfoundation.org/projects/nepal-wdf16-1432-0
44. Diabetes prevention and management by lay health workers in Nepal, WDF16-1441 [Internet]. World diabetes foundation. 2017 [cited 2022 Aug 17]. Available from: https://www.worlddiabetesfoundation.org/projects/nepal-wdf16-1441
45. Nurse-led continuum of care approach for addressing diabetes in Nepal, WDF17-1483 [Internet]. World diabetes foundation. 2018 [cited 2022 Aug 18]. Available from: https://www.worlddiabetesfoundation.org/projects/nepal-wdf17-1483
46. National Diabetes Prevention and Control Program - WDF15-1232 [Internet]. World diabetes foundation. 2017 [cited 2022 Aug 17]. Available from: https://www.worlddiabetesfoundation.org/projects/rwanda-wdf15-1232
47. 1Touch MoH transformation program to tackle diabetes and NCDs in Samoa, WDF17-1465 [Internet]. World diabetes foundation. 2018 [cited 2022 Aug 18]. Available from: https://www.worlddiabetesfoundation.org/projects/samoa-wdf17-1465
48. Seychelles adopts the World Health Organisation's Package of Essential Non-communicable diseases interventions [Internet]. WHO | Regional Office for Africa. [cited 2022 Aug 17]. Available from: https://www.afro.who.int/news/seychelles-adopts-world-health-organisations-package-essential-non-communicable-diseases
49. Improving diabetes care WDF08-396 [Internet]. World diabetes foundation. 2012 [cited 2022 Aug 18]. Available from: https://www.worlddiabetesfoundation.org/projects/solomon-islands-wdf08-396
50. General diabetes care, WDF09-411 [Internet]. World diabetes foundation. 2012 [cited 2022 Aug 18]. Available from: https://www.worlddiabetesfoundation.org/projects/sri-lanka-wdf09-411
51. Sri Lanka national NCD response programme support, WDF12-683 [Internet]. World diabetes foundation. 2012 [cited 2022 Aug 18]. Available from: https://www.worlddiabetesfoundation.org/projects/sri-lanka-wdf12-683
52. Sri Lanka Diabetes and CVD initiative, WDF15-1291 [Internet]. World diabetes foundation. 2017 [cited 2022 Aug 18]. Available from: https://www.worlddiabetesfoundation.org/projects/sri-lanka-wdf15-1291
53. Diabetes awareness, care and referral in Lake Zone - a comprehensive model of care WDF07-265 [Internet]. World diabetes foundation. 2012 [cited 2022 Aug 18]. Available from: https://www.worlddiabetesfoundation.org/projects/tanzania-wdf07-265
54. Community and school outreach WDF09-450 [Internet]. World diabetes foundation. 2013 [cited 2022 Aug 18]. Available from: https://www.worlddiabetesfoundation.org/projects/tanzania-wdf09-450
55. Tanzania National NCD Response Programme support WDF06-221 [Internet]. World diabetes foundation. 2012 [cited 2022 Aug 18]. Available from: https://www.worlddiabetesfoundation.org/projects/tanzania-wdf06-221
56. National Diabetes Primary Prevention Program WDF13-719 [Internet]. World diabetes foundation. 2014 [cited 2022 Aug 18]. Available from: https://www.worlddiabetesfoundation.org/projects/tanzania-wdf13-719

57. NCD National Action Plan 2014-2018. Ministry of Health, Timor-Leste. [Internet]. [cited 2022 Aug 18]. Available from: https://extranet.who.int/ncdccs/Data/TLS_B3_NCD%20Action%20Plan,%20Injuries,%20Disabilities,%20and%20Elderly%20Care%202014-2018.pdf
58. Training of diabetes awareness educators WDF07-237 [Internet]. World diabetes foundation. 2012 [cited 2022 Aug 18]. Available from: https://www.worlddiabetesfoundation.org/projects/togo-wdf07-237
59. Improvement in care of diabetes in Togo through decentralisation of care WDF14-917 [Internet]. World diabetes foundation. 2015 [cited 2022 Aug 18]. Available from: https://www.worlddiabetesfoundation.org/projects/togo-wdf-14-917
60. Diabetes and HIV/AIDS prevention and care in Togo, WDF17-1537 [Internet]. World diabetes foundation. 2018 [cited 2022 Aug 18]. Available from: https://www.worlddiabetesfoundation.org/projects/togo-wdf17-1537
61. Tuvalu National Strategic Plan for Non Communicable Diseases 2011-2015 [Internet]. [cited 2022 Aug 18]. Available from: https://extranet.who.int/ncdccs/Data/TUV_B3_Tuvalu%20National%20Strategic%20Plan%20NCDs%202011-15%20final%2022Mar11.pdf
62. National NCD Programme WDF06-222 [Internet]. World diabetes foundation. 2012 [cited 2022 Aug 18]. Available from: https://www.worlddiabetesfoundation.org/projects/uganda-wdf06-222
63. Diabetes Prevention through School Programme WDF13-819 [Internet]. World diabetes foundation. 2015 [cited 2022 Aug 18]. Available from: https://www.worlddiabetesfoundation.org/projects/uganda-wdf13-819
64. Optimising Care through Integrating NCD Services in Primary Care, WDF19-1721 [Internet]. World diabetes foundation. 2020 [cited 2022 Aug 18]. Available from: https://www.worlddiabetesfoundation.org/projects/wdf19-1721
65. Strategy Rollout in the Solomon Islands [Internet]. The 10000 Toes Campaign. [cited 2022 Aug 18]. Available from: https://www.10000toes.com/diabetes-prevention-strategy-rollout/
66. Bringing health screenings closer to communities in Vietnam [Internet]. [cited 2022 Aug 18]. Available from: https://www.path.org/articles/bringing-health-screenings-closer-communities-vietnam/
67. Diabetes Awareness and Treatment Programme WDF14-933 [Internet]. World diabetes foundation. 2015 [cited 2022 Aug 18]. Available from: https://www.worlddiabetesfoundation.org/projects/zambia-wdf14-933
68. NCD Support Programme Zanzibar WDF11-593 [Internet]. World diabetes foundation. 2013 [cited 2022 Aug 18]. Available from: https://www.worlddiabetesfoundation.org/projects/zanzibar-tanzania-wdf11-593
69. World Diabetes Foundation [Internet]. World diabetes foundation. [cited 2022 Aug 18]. Available from: https://www.worlddiabetesfoundation.org/projects/search

Appendix 9. Survey questionnaire wording and availability

During the past three years, has a doctor or other health worker advised you to do any of the following:	**Countries**
Start or practice more physical activity	Algeria, Bangladesh, Belarus, Benin, Bhutan, Botswana, Burkina Faso, Ecuador, Eswatini, Georgia, Iran, Iraq, Kenya, Kyrgyzstan, Lebanon, Moldova, Morocco, Myanmar, Nepal, Seychelles, Solomon Islands, Sri Lanka, Sudan, Timor Leste, Tokelau, Tuvalu, Uganda, Vietnam, Zambia
Maintain a healthy body weight or lose weight	Algeria, Bangladesh, Belarus, Benin, Bhutan, Botswana, Burkina Faso, Costa Rica, Ecuador, Eswatini, Georgia, Iran, Iraq, Kenya, Kyrgyzstan, Lebanon, Moldova, Morocco, Myanmar, Nepal, Seychelles, Solomon Islands, Sri Lanka, Sudan, Timor Leste, Tokelau, Tuvalu, Uganda, Vietnam, Zambia
Eat at least five servings of fruit and/or vegetables each day	Algeria, Bangladesh, Belarus, Benin, Bhutan, Botswana, Burkina Faso, Ecuador, Eswatini, Georgia, Iran, Iraq, Kenya, Kyrgyzstan, Lebanon, Moldova, Morocco, Myanmar, Nepal, Solomon Islands, Sri Lanka, Sudan, Timor Leste, Tokelau, Tuvalu, Uganda, Vietnam, Zambia

Notes: Bangladesh, Ecuador, Nepal, and Seychelles' surveys ask respondents about counseling in the past year instead of past three years. Costa Rica's counseling question is conditioned on whether the respondent has diabetes, hypertension, or lipids disorder, but data is available for 96.9% of the respondents included in the analysis.

Appendix 10. Characteristics of surveys conducted in 44 LMICs between 2010 and 2019, classified by World Bank income group

Country	*Survey*	*Year*	*Response rate, (%)*	*N, prior to exclusion*	*N, after exclusion*	*Female, (%)*
Low-income countries						
Benin	STEPS	2015	98.6	5,126	3,764	54.0
Burkina Faso	STEPS	2013	97.8	4,707	3,844	53.2
Cambodia	STEPS	2010	96.3	5,433	4,874	50.5
Eritrea	STEPS	2010	97.0	6,265	5,157	81.1
Liberia	STEPS	2011	87.1	2,533	1,336	53.9
Rwanda	STEPS	2012	99.0	7,226	4,993	52.4
Tanzania	STEPS	2012	94.0	5,600	4,481	49.4
Togo	STEPS	2010	96.3	4,373	2,500	51.9
Uganda	STEPS	2014	99.0	3,987	2,596	57.3
Zanzibar	STEPS	2011	97.6	2,492	2,079	50.8
Lower-middle income countries						
Bangladesh	STEPS	2018	84.6	8,185	5,521	52.7
Bhutan	STEPS	2014	96.9	2,815	2,331	41.6
Eswatini	STEPS	2014	81.8	3,531	1,890	55.7
Georgia	STEPS	2016	75.7	4,204	2,707	52.8
Kenya	STEPS	2015	95.0	4,488	3,179	50.0
Kyrgyzstan	STEPS	2013	100	2,623	2,329	48.3
Laos	STEPS	2013	99.2	2,564	1,957	57.8
Lesotho	STEPS	2012	80.0	2,310	1,888	49.1
Moldova	STEPS	2013	83.5	4,807	3,057	49.9
Morocco	STEPS	2017	89.0	5,429	3,544	49.7
Myanmar	STEPS	2014	94.0	8,271	7,143	48.6
Nepal	STEPS	2019	98.6	5,593	4,107	53.6
Samoa	STEPS	2013	64.0	1,766	975	48.0
Solomon Islands	STEPS	2015	58.4	2,525	1,384	51.5
Sri Lanka	STEPS	2014	72.0	5,188	3,305	49.4
Sudan	STEPS	2016	88.0	7,722	4,786	45.2
Timor Leste	STEPS	2014	94.7	2,609	1,961	56.5
Vanuatu	STEPS	2011	94.0	4,643	3,634	52.4
Vietnam	STEPS	2015	79.8	3,758	2,539	50.3
Zambia	STEPS	2017	74.3	4,302	2,334	49.7
Upper-middle income countries						
Algeria	STEPS	2016	93.0	6,989	4,501	48.3
Azerbaijan	STEPS	2017	97.0	2,801	2,131	50.4
Belarus	STEPS	2016	87.1	5,010	4,161	52.2
Botswana	STEPS	2014	64.0	4,070	2,456	48.5
*Chile	NHS	2009-10	85.0	5,293	3,606	51.6

Costa Rica	STEPS	2010	87.5	3,684	2,058	50.5
Ecuador	STEPS	2018	69.5	4,638	3,020	52.1
Iran	STEPS	2016	99.0	30,541	17,493	53.6
Iraq	STEPS	2015	93.0	4,071	2,539	47.7
Lebanon	STEPS	2017	65.9	1,899	931	54.0
Marshall Islands	STEPS	2017	92.3	3,015	1,543	51.1
Namibia	DHS	2013	96.9	4,403	3,026	60.4
Seychelles	STEPS	2013	73.0	1,240	1,061	50.4
Tokelau	STEPS	2014	70.0	554	277	54.0
Tuvalu	STEPS	2015	76.0	1,155	741	44.3
Global		2010-2019	87.0	214,438	145,739	51.9

Note: Participants who were pregnant, below the age of 25, or had diabetes were not included in the analysis. Additionally, those who responded no to fasting at time of response and did not meet the BMI criterion to be considered high-risk for diabetes were likewise excluded. Female (%) calculated using sampling weights with equal weighting for each survey. *The Chile NHS survey began data collection in 2009, which continued until 2010. Abbreviations: DHS= Demographic and Health Survey, NHS=National Health Survey, STEPS=Stepwise Approach to Surveillance

Appendix 11. Countries with available data for each diabetes prevention service

Prevention service	Countries
Counseling to increase physical activity	Algeria, Bangladesh, Belarus, Benin, Bhutan, Botswana, Burkina Faso, Ecuador, Eswatini, Georgia, Iran, Iraq, Kenya, Kyrgyzstan, Lebanon, Liberia, Moldova, Morocco, Myanmar, Nepal, Seychelles, Solomon Islands, Sri Lanka, Sudan, Timor Leste, Tokelau, Tuvalu, Uganda, Vietnam, Zambia
Counseling to reduce body weight	Algeria, Bangladesh, Belarus, Benin, Bhutan, Botswana, Burkina Faso, Costa Rica, Ecuador, Eswatini, Georgia, Iran, Iraq, Kenya, Kyrgyzstan, Lebanon, Moldova, Morocco, Myanmar, Nepal, Seychelles, Solomon Islands, Sri Lanka, Sudan, Timor Leste, Tokelau, Tuvalu, Uganda, Vietnam, Zambia
Counseling to increase dietary fruit and vegetable intake	Algeria, Bangladesh, Belarus, Benin, Bhutan, Botswana, Burkina Faso, Ecuador, Eswatini, Georgia, Iran, Iraq, Kenya, Kyrgyzstan, Lebanon, Moldova, Morocco, Myanmar, Nepal, Solomon Islands, Sri Lanka, Sudan, Timor Leste, Tokelau, Tuvalu, Uganda, Vietnam, Zambia
Blood glucose screening	Algeria, Azerbaijan, Bangladesh, Belarus, Benin, Bhutan, Botswana, Burkina Faso, Cambodia, Chile, Costa Rica, Ecuador, Eritrea, Eswatini, Georgia, Iran, Iraq, Kenya, Kyrgyzstan, Laos, Lebanon, Lesotho, Liberia, Marshall Islands, Moldova, Morocco, Myanmar, Namibia, Nepal, Rwanda, Samoa, Seychelles, Solomon Islands, Sri Lanka, Sudan, Tanzania, Timor Leste, Togo, Tokelau, Tuvalu, Uganda, Vanuatu, Vietnam, Zambia, Zanzibar

Appendix 12. Proportion deemed at high risk of diabetes, classified by geographic region

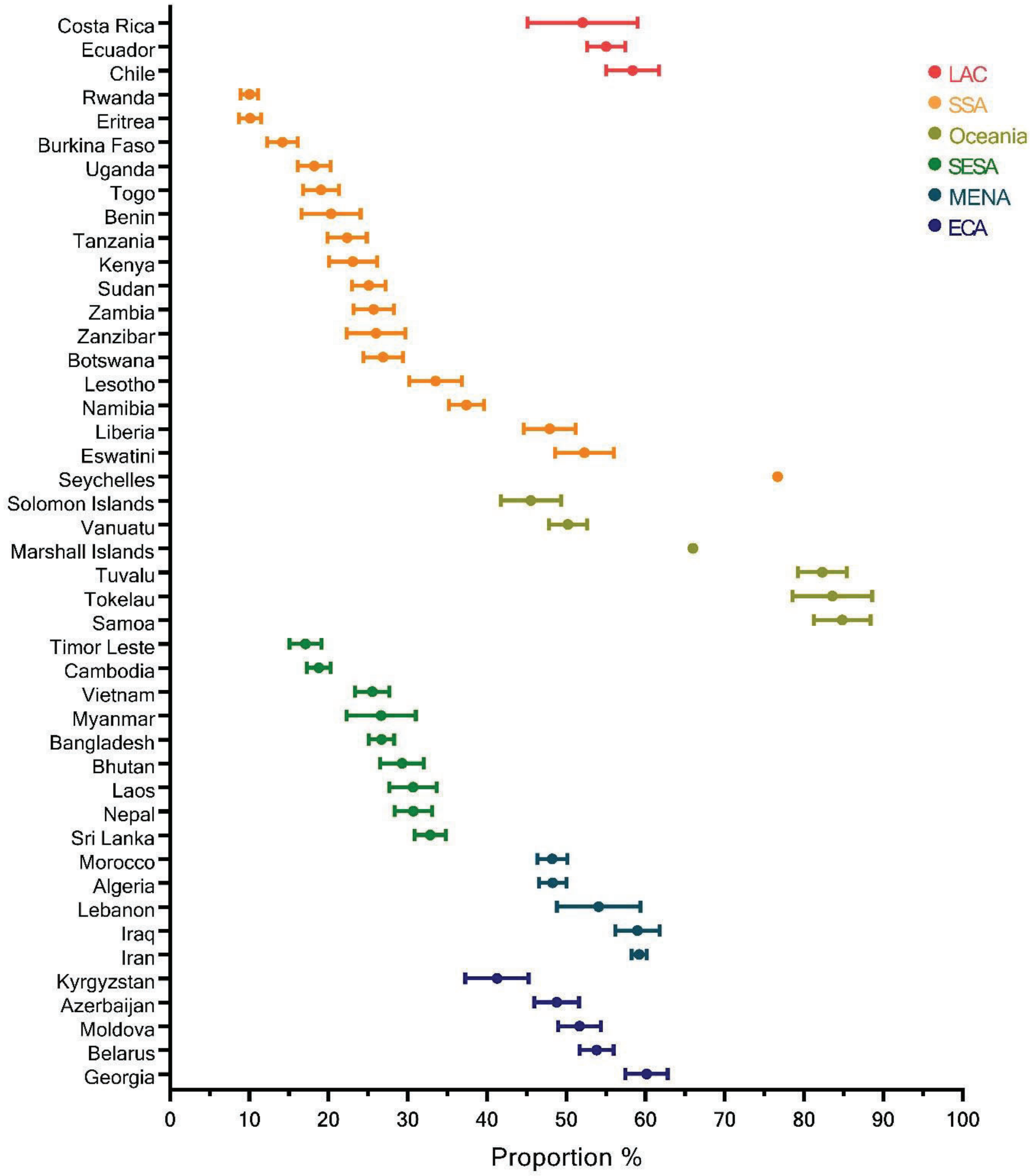

Notes: Figure shows estimated proportion of each country's population deemed high -risk for developing diabetes. Error bars represent 95% confidence intervals and were calculated with sampling weights readjusted to weigh each country equally.

Abbreviations: ECA = Europe and Central Asia; LAC = Latin America and the Caribbean; MENA = Middle East and North Africa; SESA = South, East, and Southeast Asia; SSA = sub-Saharan Africa

Appendix 13. Table of prevalence rates for the population deemed at high risk of diabetes, classified by geographic region

Country	Proportion high-risk (%)	95% Confidence interval
Latin America & the Caribbean	55.2	(52.3 - 58.0)
Chile	58.4	(55.0 - 61.7)
Costa Rica	52.1	(45.1 - 59.0)
Ecuador	55.0	(52.6 - 57.4)
Sub-Saharan Africa	28.7	(23.5 - 34.6)
Benin	20.2	(16.7 - 24.1)
Botswana	26.8	(24.4 - 29.4)
Burkina Faso	14.1	(12.3 - 16.2)
Eritrea	10.0	(8.7 - 11.5)
Eswatini	52.3	(48.6 - 56)
Kenya	23.0	(20.1 - 26.1)
Lesotho	33.4	(30.2 - 36.8)
Liberia	47.9	(44.7 - 51.2)
Namibia	37.4	(35.2 - 39.6)
Rwanda	10.0	(8.9 - 11.2)
Seychelles	76.7	(76.7 - 76.7)
Sudan	25.1	(23.0 - 27.2)
Tanzania	22.3	(19.9 - 24.9)
Togo	19.0	(16.9 - 21.3)
Uganda	18.1	(16.1 - 20.3)
Zambia	25.6	(23.2 - 28.3)
Zanzibar	25.9	(22.4 - 29.7)
Oceania	69.0	(66.2 - 71.6)
Marshall Islands	66.0	(66.0 - 66.0)
Samoa	85.1	(81.1 - 88.3)
Solomon Islands	45.5	(41.8 - 49.3)
Tokelau	84.0	(78.4 - 88.4)
Tuvalu	82.4	(79.1 - 85.3)
Vanuatu	50.2	(47.8 - 52.6)
South/East/Southeast Asia	26.4	(25.6 - 27.3)
Bangladesh	26.7	(25.1 - 28.3)
Bhutan	29.2	(26.6 - 32.1)
Cambodia	18.7	(17.3 - 20.3)
Laos	30.6	(27.7 - 33.7)
Myanmar	26.5	(22.3 - 31.1)

Nepal	30.7	(28.3 - 33.1)
Sri Lanka	32.8	(30.9 - 34.8)
Timor Leste	17.0	(15.1 - 19.1)
Vietnam	25.5	(23.4 - 27.7)
Middle East & North Africa	53.8	(52.4 - 55.1)
Algeria	48.3	(46.5 - 50.0)
Iran	59.2	(58.2 - 60.2)
Iraq	59.0	(56.2 - 61.8)
Lebanon	54.1	(48.8 - 59.3)
Morocco	48.2	(46.3 - 50.1)
Europe & Central Asia	51.1	(49.7 - 52.6)
Azerbaijan	48.8	(46.0 - 51.6)
Belarus	53.8	(51.7 - 56.0)
Georgia	60.1	(57.4 - 62.8)
Kyrgyzstan	41.2	(37.3 - 45.3)
Moldova	51.7	(49.0 - 54.4)

Notes: Estimates of prevalence were calculated with sampling weights readjusted to weigh each country equally

Appendix 14. Table of prevalence rates for the population deemed at high risk of diabetes, classified by World Bank income groups

Country	Proportion high-risk (%)	95% Confidence interval
Low-income countries	20.6	(19.8 - 21.5)
Benin	20.2	(16.7 - 24.1)
Burkina Faso	14.1	(12.3 - 16.2)
Cambodia	18.7	(17.3 - 20.3)
Eritrea	10.0	(8.7 - 11.5)
Liberia	47.9	(44.7 - 51.2)
Rwanda	10.0	(8.9 - 11.2)
Tanzania	22.3	(19.9 - 24.9)
Togo	19.0	(16.9 - 21.3)
Uganda	18.1	(16.1 - 20.3)
Zanzibar	25.9	(22.4 - 29.7)
Lower-middle income countries	38.0	(37.2 - 38.9)
Bangladesh	26.7	(25.1 - 28.3)
Bhutan	29.2	(26.6 - 32.1)
Eswatini	52.3	(48.6 - 56)
Georgia	60.1	(57.4 - 62.8)
Kenya	23.0	(20.1 - 26.1)
Kyrgyzstan	41.2	(37.3 - 45.3)
Laos	30.6	(27.7 - 33.7)
Lesotho	33.4	(30.2 - 36.8)
Moldova	51.7	(49 - 54.4)
Morocco	48.2	(46.3 - 50.1)
Myanmar	26.5	(22.3 - 31.1)
Nepal	30.7	(28.3 - 33.1)
Samoa	85.1	(81.1 - 88.3)
Solomon Islands	45.5	(41.8 - 49.3)
Sri Lanka	32.8	(30.9 - 34.8)
Sudan	25.1	(23 - 27.2)
Timor Leste	17.0	(15.1 - 19.1)
Vanuatu	50.2	(47.8 - 52.6)
Vietnam	25.5	(23.4 - 27.7)
Zambia	25.6	(23.2 - 28.3)
Upper-middle income countries	57.5	(54.3 - 60.6)
Algeria	48.3	(46.5 - 50)
Azerbaijan	48.8	(46 - 51.6)
Belarus	53.8	(51.7 - 56)
Botswana	26.8	(24.4 - 29.4)
Chile	58.4	(55 - 61.7)
Costa Rica	52.1	(45.1 - 59)
Ecuador	55.0	(52.6 - 57.4)
Iran	59.2	(58.2 - 60.2)
Iraq	59.0	(56.2 - 61.8)
Lebanon	54.1	(48.8 - 59.3)

Marshall Islands	66.0	(66 - 66)
Namibia	37.4	(35.2 - 39.6)
Seychelles	76.7	(76.7 - 76.7)
Tokelau	84.0	(78.4 - 88.4)
Tuvalu	82.4	(79.1 - 85.3)

Notes: Estimates of prevalence were calculated with sampling weights readjusted to weigh each country equally

Appendix 15. Table of proportion of the population deemed at high risk of diabetes who reported receiving physical activity counseling, classified by World Bank income groups

Country	Unweighted N	Proportion who received (95% CI)
Low-income countries	352	20.2 (17.2 - 23.6)
Benin	140	14.7 (10.7 - 19.9)
Burkina Faso	100	22.7 (18.1 - 28.1)
Uganda	112	23.2 (17.3 - 30.4)
Lower-middle income countries	6,626	36.9 (35.5 - 38.2)
Bangladesh	252	27.1 (22.9 - 31.8)
Bhutan	427	52.8 (47.7 - 57.8)
Eswatini	506	58.0 (53.4 - 62.6)
Georgia	510	27.9 (25.0 – 31.0)
Kenya	124	17.8 (13.4 - 23.3)
Kyrgyzstan	521	43.0 (37.5 - 48.6)
Moldova	1,018	57.4 (53.1 - 61.5)
Morocco	766	39.5 (37.2 – 42.0)
Myanmar	921	42.1 (37.2 - 47.1)
Nepal	156	35.2 (26.7 - 44.6)
Solomon Islands	282	40.7 (35.8 - 45.8)
Sri Lanka	423	33.5 (30.2 - 37)
Sudan	276	21.3 (18.5 - 24.4)
Timor Leste	119	35.8 (28.9 - 43.4)
Vietnam	258	33.0 (28.9 - 37.3)
Zambia	67	24.8 (19 - 31.6)
Upper-middle income countries	10,185	50.9 (45.9 - 55.8)
Algeria	1,098	46.4 (43.2 - 49.6)
Belarus	1,137	45.6 (41.4 - 49.8)
Botswana	504	57.3 (51.9 - 62.5)
Ecuador	749	73.9 (70.3 - 77.2)
Iran	4,911	48.2 (46.8 - 49.7)
Iraq	784	48.8 (45 - 52.5)
Lebanon	267	42.6 (35.8 - 49.8)
Seychelles	234	27.1 (27.1 - 27.1)
Tokelau	146	60.1 (56.3 - 63.7)
Tuvalu	355	58.8 (51.5 - 65.7)

Notes: Estimates of prevalence were calculated with sampling weights readjusted to weigh each country equally

Appendix 16. Table of proportion of the population deemed at high risk of diabetes who reported receiving body weight counseling, classified by World Bank income groups

Country	Unweighted N	Proportion who received (95% CI)
Low-income countries	296	17.1 (14.1 - 20.6)
Benin	113	11.6 (7.7 - 17)
Burkina Faso	79	18.7 (13.9 - 24.6)
Uganda	104	21.1 (15.2 - 28.5)
Lower-middle income countries	6,076	32.8 (31.5 - 34.1)
Bangladesh	244	23.1 (19.2 - 27.5)
Bhutan	322	41.1 (36.4 – 46.0)
Eswatini	431	48.7 (43.9 - 53.6)
Georgia	547	29.3 (26.4 - 32.4)
Kenya	127	18.0 (13.7 - 23.2)
Kyrgyzstan	520	41.4 (36.6 - 46.5)
Moldova	1,044	58.3 (54.1 - 62.3)
Morocco	665	33.9 (31.6 - 36.2)
Myanmar	765	32.6 (27.8 - 37.9)
Nepal	112	30.4 (21.9 - 40.4)
Solomon Islands	264	37.9 (32.7 - 43.5)
Sri Lanka	438	33.8 (30.3 - 37.4)
Sudan	243	19.7 (16.5 - 23.2)
Timor Leste	100	30.1 (23.5 - 37.7)
Vietnam	200	25.9 (22.3 - 29.8)
Zambia	54	20.6 (14.6 - 28.3)
Upper-middle income countries	9,409	48.8 (44.8 - 52.9)
Algeria	1,065	44.8 (41.4 - 48.2)
Belarus	1,302	52.3 (48.3 - 56.3)
Botswana	468	52.3 (47 - 57.6)
Costa Rica	470	42.6 (36.5 - 48.9)
Ecuador	771	75.2 (71.5 - 78.6)
Iran	3,495	33.8 (32.5 - 35.2)
Iraq	830	50.7 (46.9 - 54.4)
Lebanon	287	44.3 (36.9 - 52.1)
Seychelles	243	28.0 (28.0 – 28.0)
Tokelau	138	57.4 (50.0 - 64.4)
Tuvalu	340	55.9 (49.5 - 62.1)

Notes: Estimates of prevalence were calculated with sampling weights readjusted to weigh each country equally

Appendix 17. Table of proportion of the population deemed at high risk of diabetes who reported receiving dietary fruit and vegetable counseling, classified by World Bank income groups

Country	Unweighted N	Proportion who received (95% CI)
Low-income countries	373	20.2 (17.3 - 23.5)
Benin	142	15.4 (11.3 - 20.7)
Burkina Faso	73	14.1 (11 - 18)
Uganda	158	31.2 (25 - 38.1)
Lower-middle income countries	7,329	42.9 (41.5 - 44.3)
Bangladesh	422	42.5 (37.4 - 47.8)
Bhutan	539	62.3 (57.6 - 66.8)
Eswatini	517	58.4 (53.5 - 63.2)
Georgia	461	25.0 (22.1 - 28.1)
Kenya	192	24.9 (20.2 - 30.3)
Kyrgyzstan	665	51.4 (45.8 - 57.1)
Moldova	1,191	65.8 (62.0 - 69.4)
Morocco	711	35.9 (33.5 - 38.2)
Myanmar	999	45.1 (38.4 – 52.0)
Nepal	261	58.2 (48.8 - 67.1)
Solomon Islands	265	38.0 (32.5 - 43.7)
Sri Lanka	447	35.0 (31.6 - 38.6)
Sudan	277	20.8 (17.8 - 24.1)
Timor Leste	151	45.5 (38.5 - 52.7)
Vietnam	131	41.2 (34.9 - 47.8)
Zambia	100	36.1 (29.8 - 42.9)
Upper-middle income countries	9,733	(48.2 - 51.2)
Algeria	905	37.3 (33.9 - 40.8)
Belarus	1,100	43.7 (39.5 - 48)
Botswana	494	52.3 (46.9 - 57.5)
Ecuador	765	72.4 (68.6 - 75.8)
Iran	4,976	48.1 (46.6 - 49.6)
Iraq	800	48.2 (44.4 – 52.0)
Lebanon	219	33.3 (27.2 – 40.0)
Tokelau	144	59.2 (55.8 - 62.6)
Tuvalu	330	52.9 (46.8 – 59.0)

Notes: Estimates of prevalence were calculated with sampling weights readjusted to weigh each country equally

Appendix 18. Table of proportion of the population deemed at high risk of diabetes who reported receiving blood glucose screening, classified by World Bank income groups

Country	Unweighted N	Proportion who received (95% CI)
Low-income countries	1,003	15.4 (14.1 - 16.8)
Benin	44	5.4 (3.2 - 8.7)
Burkina Faso	60	12.6 (9.1 - 17.4)
Cambodia	236	20.3 (17.5 - 23.3)
Eritrea	100	19.6 (15.3 - 24.7)
Liberia	56	8.8 (6.4 - 11.9)
Rwanda	44	8.1 (5.9 - 10.9)
Tanzania	158	20.7 (15.4 - 27.2)
Togo	58	16.6 (12.0 - 22.5)
Uganda	72	14.2 (10.8 - 18.4)
Zanzibar	175	28.0 (23.3 - 33.2)
Lower-middle income countries	8,951	32.6 (31.6 - 33.7)
Bangladesh	690	40.8 (37.3 - 44.3)
Bhutan	220	23.7 (20.0 - 27.8)
Eswatini	303	33.7 (30.0 - 37.7)
Georgia	833	42.5 (39.0 – 46.0)
Kenya	232	25.6 (21.1 - 30.7)
Kyrgyzstan	407	31.5 (27.8 - 35.5)
Laos	119	21.0 (16.9 - 25.7)
Lesotho	205	26.3 (20.9 - 32.5)
Moldova	1,354	68.0 (64.5 - 71.4)
Morocco	914	45.0 (42.6 - 47.4)
Myanmar	585	20.2 (15.7 - 25.5)
Nepal	322	24.1 (19.4 - 29.6)
Samoa	265	28.8 (22.9 - 35.5)
Solomon Islands	268	37.1 (31.9 - 42.7)
Sri Lanka	794	62.6 (58.8 - 66.3)
Sudan	364	25.9 (22.4 - 29.6)
Timor Leste	10	3 .0 (1.6 - 5.7)
Vanuatu	645	36.0 (31.8 - 40.4)
Vietnam	337	41.4 (37.2 - 45.8)
Zambia	84	15.2 (12.0 - 19.1)
Upper-middle income countries	17,278	57.6 (53.5 - 61.7)

Algeria	1,290	53.1 (49.6 - 56.6)
Azerbaijan	492	36.4 (32.5 - 40.4)
Belarus	2,256	91.5 (89.3 - 93.3)
Botswana	396	46.4 (41.4 - 51.5)
Chile	1,274	56.6 (52.6 - 60.6)
Costa Rica	710	55.6 (49.7 - 61.4)
Ecuador	930	55.2 (51.9 - 58.4)
Iran	6,614	66.1 (64.7 - 67.4)
Iraq	887	76.4 (72.3 - 80.1)
Lebanon	238	31.6 (24.9 - 39.2)
Marshall Islands	553	54.3 (54.3 - 54.3)
Namibia	395	33.4 (29.6 - 37.4)
Seychelles	749	87.4 (87.4 - 87.4)
Tokelau	170	70.2 (63.1 - 76.4)
Tuvalu	324	50.2 (43.1 - 57.2)

Notes: Estimates of prevalence were calculated with sampling weights readjusted to weigh each country equally

Appendix 19. Univariate regression models of physical activity counseling

Variables	Risk ratio	Robust 95% CI
Age		
35-44 years	1.04	(0.96 - 1.12)
45-54 years	1.07*	(0.99 - 1.16)
55-64 years	1.11***	(1.03 - 1.20)
65 years and older	1	(0.91 - 1.11)
Observations	40435	
Number of countries	30	
Sex		
Female	1.03	(0.98 - 1.07)
Observations	40435	
Number of countries	30	
Education		
Less than primary school	1.22***	(1.13 - 1.32)
Primary school completed	1.46***	(1.35 - 1.57)
Observations	39,538	
Number of countries	29	

*** p<0.01, ** p<0.05, * p<0.1

Notes: Univariate Poisson regressions estimated with country-level fixed effects and sampling weights rescaled to weigh each country equally.

Appendix 20. Univariate regression models of body weight counseling

Variables	Risk ratio	Robust 95% CI
Age		
35-44 years	1.06	(0.99 - 1.14)
45-54 years	1.03	(0.95 - 1.12)
55-64 years	1.09**	(1.01 - 1.18)
65 years and older	1.01	(0.91 - 1.11)
Observations	41659	
Number of countries	31	
Sex		
Female	1.14***	(1.09 - 1.19)
Observations	41658	
Number of countries	31	
Education		
Less than primary school	1.28***	(1.18 - 1.40)
Primary school completed	1.54***	(1.41 - 1.67)
Observations	40,697	
Number of countries	30	

*** p<0.01, ** p<0.05, * p<0.1

Notes: Univariate Poisson regressions estimated with country-level fixed effects and sampling weights rescaled to weigh each country equally.

Appendix 21. Univariate regression models of dietary fruit and vegetable counseling

Variables	Risk ratio	Robust 95% CI
Age		
35-44 years	1.02	(0.95 - 1.10)
45-54 years	1.07**	(1.00 - 1.14)
55-64 years	1.12***	(1.05 - 1.19)
65 years and older	1.16***	(1.06 - 1.26)
Observations	39173	
Number of countries	29	
Sex		
Female	1.08***	(1.03 - 1.12)
Observations	39173	
Number of countries	29	
Education		
Less than primary school	1.04	(0.97 - 1.11)
Primary school completed	1.09**	(1.02 - 1.17)
Observations	38,277	
Number of countries	28	

*** p<0.01, ** p<0.05, * p<0.1

Notes: Univariate Poisson regressions estimated with country-level fixed effects and sampling weights rescaled to weigh each country equally.

Appendix 22. Univariate regression models of blood glucose measuring

Variables	Risk ratio	Robust 95% CI
Age		
35-44 years	1.21***	(1.13 - 1.31)
45-54 years	1.40***	(1.29 - 1.51)
55-64 years	1.52***	(1.41 - 1.64)
65 years and older	1.58***	(1.46 - 1.71)
Observations	58045	
Number of countries	45	
Sex		
Female	1.11***	(1.07 - 1.14)
Observations	58044	
Number of countries	45	
Education		
Less than primary school	1.26***	(1.18 - 1.34)
Primary school completed	1.64***	(1.54 - 1.75)
Observations	57,262	
Number of countries	44	

*** p<0.01, ** p<0.05, * p<0.1

Notes: Univariate Poisson regressions estimated with country-level fixed effects and sampling weights rescaled to weigh each country equally.

Appendix 23. Multivariate regression model of physical activity counseling

Variables	Risk ratio	Robust 95% CI
Age		
35-44 years	1.07*	(0.99 – 1.16)
45-54 years	1.13***	(1.04 – 1.22)
55-64 years	1.22***	(1.13 – 1.32)
65 years and older	1.16***	(1.05 – 1.28)
Sex		
Female	1.07***	(1.03 – 1.12)
Education		
Less than primary school	1.26***	(1.17 – 1.36)
Primary school completed	1.55***	(1.44 – 1.67)
Observations	39,538	
Number of countries	29	

*** p<0.01, ** p<0.05, * p<0.1

Notes: Multivariate Poisson regressions estimated with country-level fixed effects and sampling weights rescaled to weigh each country equally.

Appendix 24. Multivariate regression models of body weight counseling

Variables	Risk ratio	Robust 95% CI
Age		
35-44 years	1.10**	(1.02 – 1.18)
45-54 years	1.09**	(1.01 – 1.18)
55-64 years	1.21***	(1.12 – 1.31)
65 years and older	1.19***	(1.08 – 1.32)
Sex		
Female	1.19***	(1.13 – 1.24)
Education		
Less than primary school	1.35***	(1.24 – 1.47)
Primary school completed	1.66***	(1.52 – 1.81)
Observations	40,696	
Number of countries	30	

*** p<0.01, ** p<0.05, * p<0.1

Notes: Multivariate Poisson regressions estimated with country-level fixed effects and sampling weights rescaled to weigh each country equally.

Appendix 25. Multivariate regression model of dietary fruit and vegetable counseling

Variables	Risk ratio	Robust 95% CI
Age		
35-44 years	1.04	(0.96 – 1.12)
45-54 years	1.09**	(1.01 – 1.17)
55-64 years	1.16***	(1.07 – 1.24)
65 years and older	1.23***	(1.12 – 1.35)
Sex		
Female	1.10***	(1.06 – 1.15)
Education		
Less than primary school	1.08**	(1.01 – 1.16)
Primary school completed	1.17***	(1.09 – 1.26)
Observations	38,277	
Number of countries	28	

*** p<0.01, ** p<0.05, * p<0.1

Notes: Multivariate Poisson regressions estimated with country-level fixed effects and sampling weights rescaled to weigh each country equally.

Appendix 26. Multivariate regression model of blood glucose measuring

Variables	Risk ratio	Robust 95% CI
Age		
35-44 years	1.25***	(1.15 – 1.36)
45-54 years	1.49***	(1.36 – 1.63)
55-64 years	1.72***	(1.57 – 1.88)
65 years and older	1.89***	(1.72 – 2.07)
Sex		
Female	1.16***	(1.12 – 1.19)
Education		
Less than primary school	1.39***	(1.30 – 1.47)
Primary school completed	1.93***	(1.81 – 2.05)
Observations	57,261	
Number of countries	44	

*** p<0.01, ** p<0.05, * p<0.1

Notes: Multivariate Poisson regressions estimated with country-level fixed effects and sampling weights rescaled to weigh each country equally.

Appendix 27. Defining high-risk for developing diabetes using the Finnish Diabetes Risk Score (FINDRISC)

We were able to leverage the FINDRISC calculator developed by Lindström and Tuomilehto (2003) to define the high-risk population for a sensitivity analysis. Each component of the original FINDRISC calculator is included in the below table. We were able to apply some of the criteria in their original forms, with exceptions made for “Use of blood pressure medication,” “History of high blood glucose,” “Daily consumption of fruits or vegetables,” “Physical activity < 4 hours a week,” and “Family history of diabetes.” For “Use of blood pressure medication,” we counted either reporting hypertension medication or having hypertension as affirmative use of blood pressure medication. For “History of high blood glucose,” we considered a fasting plasma glucose measure between 6.1 and 6.9 mmol/L inclusive as an affirmative. For “Daily consumption of fruits or vegetables,” we summed the days out of the week the respondent reported fruit consumption and the days out of the week reported vegetable consumption and considered all sums greater than or equal seven as daily fruit or vegetable consumption. Because of a lack of data, the categories for “Physical activity < 4 hours a week” and “Family history of diabetes” were omitted (asterisked and highlighted in the table), resulting in the maximum score possible in our analysis to be 19 (relative to the original score of 26). As such, the threshold for ‘high risk’ was lowered from 13 in the original form of the calculator to 9 for the purpose of this study.

Risk factor		Points
Age		
< 45 years of age		0
45 – 54 years of age		2
55 – 64 years of age		3
> 64 years of age		4
BMI (kg/m²)		
< 25		0
25 – 30		1
> 30		3
Waist circumference (cm)		
Men	Women	
< 94	< 80	0
94 – 101	80 – 87	3
> 101	> 87	4
Use of blood pressure medication		
No		0
Yes		2
History of high blood glucose		
No		0
Yes		5
Physical activity < 4 hours a week*		
No		0
Yes		2
Daily consumption of fruits or vegetables		
Yes		0
No		1
Family history of diabetes*		
No		0
Yes, 2nd degree relative		3
Yes, 1st degree relative		5

Reference:

Lindström J, Tuomilehto J. The diabetes risk score: a practical tool to predict type 2 diabetes risk. Diabetes Care. 2003 Mar;26(3):725-31. doi: 10.2337/diacare.26.3.725. PMID: 12610029.

Appendix 28. Multivariate regression model of physical activity counseling, adjusting for wealth

Variables	Risk ratio	Robust 95% CI
Age		
35-44 years	1.08	(0.98, 1.20)
45-54 years	1.16***	(1.06, 1.27)
55-64 years	1.24***	(1.13, 1.36)
65 years and older	1.16**	(1.01, 1.34)
Sex		
Female	1.11***	(1.05, 1.18)
Education		
Less than primary school	1.24***	(1.13, 1.35)
Primary school completed	1.43***	(1.29, 1.57)
Wealth quintile		
Poorer	1.02	(0.92, 1.12)
Middle	1.09**	(1.00, 1.20)
Richer	1.25***	(1.13, 1.38)
Richest	1.23***	(1.12, 1.36)
Observations	29,448	
Number of countries	23	

*** p<0.01, ** p<0.05, * p<0.1

Notes: Multivariate Poisson regressions estimated with country-level fixed effects and sampling weights rescaled to weigh each country equally.

Appendix 29. Multivariate regression model of body weight counseling, adjusting for wealth

Variables	Risk ratio	Robust 95% CI
Age		
35-44 years	1.14***	(1.04, 1.25)
45-54 years	1.18***	(1.07, 1.29)
55-64 years	1.31***	(1.17, 1.47)
65 years and older	1.29***	(1.14, 1.45)
Sex		
Female	1.24***	(1.16, 1.32)
Education		
Less than primary school	1.38***	(1.25, 1.53)
Primary school completed	1.57***	(1.41, 1.75)
Wealth quintile		
Poorer	1.06	(0.96, 1.18)
Middle	1.12**	(1.02, 1.23)
Richer	1.34***	(1.21, 1.48)
Richest	1.32***	(1.20, 1.46)
Observations	29,448	
Number of countries	23	

*** p<0.01, ** p<0.05, * p<0.1

Notes: Multivariate Poisson regressions estimated with country-level fixed effects and sampling weights rescaled to weigh each country equally.

Appendix 30. Multivariate regression model of dietary fruit and vegetable counseling, adjusting for wealth

Variables	Risk ratio	Robust 95% CI
Age		
35-44 years	1.07	(0.97, 1.18)
45-54 years	1.16***	(1.06, 1.27)
55-64 years	1.22***	(1.11, 1.34)
65 years and older	1.34***	(1.18, 1.52)
Sex		
Female	1.15***	(1.08, 1.21)
Education		
Less than primary school	1.11**	(1.02, 1.2)
Primary school completed	1.19***	(1.09, 1.3)
Wealth quintile		
Poorer	1	(0.92, 1.09)
Middle	1.08*	(0.99, 1.17)
Richer	1.17***	(1.07, 1.29)
Richest	1.11**	(1.02, 1.21)
Observations	28,614	
Number of countries	22	

*** p<0.01, ** p<0.05, * p<0.1

Notes: Multivariate Poisson regressions estimated with country-level fixed effects and sampling weights rescaled to weigh each country equally.

Appendix 31. Multivariate regression model of blood glucose measuring, adjusting for wealth

Variables	Risk ratio	Robust 95% CI
Age		
35-44 years	1.31***	(1.16, 1.47)
45-54 years	1.56***	(1.37, 1.76)
55-64 years	1.89***	(1.67, 2.15)
65 years and older	2.03***	(1.73, 2.39)
Sex		
Female	1.18***	(1.13, 1.23)
Education		
Less than primary school	1.34***	(1.24, 1.46)
Primary school completed	1.75***	(1.61, 1.90)
Wealth quintile		
Poorer	1.08**	(1.01, 1.16)
Middle	1.2***	(1.12, 1.28)
Richer	1.33***	(1.23, 1.44)
Richest	1.61***	(1.50, 1.74)
Observations	40,927	
Number of countries	36	

*** p<0.01, ** p<0.05, * p<0.1

Notes: Multivariate Poisson regressions estimated with country-level fixed effects and sampling weights rescaled to weigh each country equally.

Appendix 32. Multivariate regression model of physical activity, adjusting for rurality

Variables	Risk ratio	Robust 95% CI
Age		
35-44 years	1.06	(0.95, 1.19)
45-54 years	1.11	(1.00, 1.24)
55-64 years	1.22	(1.09, 1.35)
65 years and older	1.16	(1.02, 1.33)
Sex		
Female	1.03	(0.98, 1.09)
Education		
Less than primary school	1.17	(1.06, 1.28)
Primary school completed	1.49	(1.34, 1.65)
Rurality		
Rural	0.84	(.79, .9)
Observations	29,757	
Number of countries	18	

*** p<0.01, ** p<0.05, * p<0.1

Notes: Multivariate Poisson regressions estimated with country-level fixed effects and sampling weights rescaled to weigh each country equally.

Appendix 33. Multivariate regression model of body weight counseling, adjusting for rurality

Variables	Risk ratio	Robust 95% CI
Age		
35-44 years	1.06	(0.94, 1.19)
45-54 years	1.08	(0.97, 1.21)
55-64 years	1.17	(1.05, 1.31)
65 years and older	1.20	(1.04, 1.39)
Sex		
Female	1.17	(1.10, 1.25)
Education		
Less than primary school	1.26	(1.13, 1.41)
Primary school completed	1.66	(1.47, 1.87)
Rurality		
Rural	0.81	(0.76, 0.87)
Observations	29,757	
Number of countries	18	

*** p<0.01, ** p<0.05, * p<0.1

Notes: Multivariate Poisson regressions estimated with country-level fixed effects and sampling weights rescaled to weigh each country equally.

Appendix 34. Multivariate regression model of dietary fruit and vegetable counseling, adjusting for rurality

Variables	Risk ratio	Robust 95% CI
Age		
35-44 years	1.07	(0.96, 1.20)
45-54 years	1.12	(1.01, 1.23)
55-64 years	1.18	(1.06, 1.31)
65 years and older	1.31	(1.16, 1.48)
Sex		
Female	1.09	(1.03, 1.15)
Education		
Less than primary school	1.06	(0.98, 1.15)
Primary school completed	1.16	(1.06, 1.28)
Rurality		
Rural	0.95	(0.89, 1.02)
Observations	29,330	
Number of countries	18	

*** p<0.01, ** p<0.05, * p<0.1

Notes: Multivariate Poisson regressions estimated with country-level fixed effects and sampling weights rescaled to weigh each country equally.

Appendix 35. Multivariate regression model of blood glucose measuring,, adjusting for rurality

Variables	Risk ratio	Robust 95% CI
Age		
35-44 years	1.23	(1.11, 1.35)
45-54 years	1.44	(1.31, 1.59)
55-64 years	1.60	(1.45, 1.76)
65 years and older	1.76	(1.60, 1.95)
Sex		
Female	1.17	(1.13, 1.22)
Education		
Less than primary school	1.30	(1.21, 1.39)
Primary school completed	1.72	(1.60, 1.85)
Rurality		
Rural	0.76	(0.72, 0.79)
Observations	39,546	
Number of countries	25	

*** p<0.01, ** p<0.05, * p<0.1

Notes: Multivariate Poisson regressions estimated with country-level fixed effects and sampling weights rescaled to weigh each country equally.

Appendix 36. Multivariate regression model of physical activity counseling, restricted to those considered at high risk of diabetes due to BMI and age.

Variables	Risk ratio	Robust 95% CI
Age		
35-44 years	1.01	(0.93, 1.09)
45-54 years	1.06	(0.98, 1.15)
55-64 years	1.16***	(1.07, 1.26)
65 years and older	1.12**	(1.00, 1.24)
Sex		
Female	1.06***	(1.02, 1.11)
Education		
Less than primary school	1.29***	(1.20, 1.40)
Primary school completed	1.58***	(1.46, 1.71)
Observations	35,889	
Number of countries	29	

*** p<0.01, ** p<0.05, * p<0.1

Notes: Multivariate Poisson regressions estimated with country-level fixed effects and sampling weights rescaled to weigh each country equally.

Appendix 37. Multivariate regression model of body weight counseling, restricted to those considered at high risk of diabetes due to BMI and age.

Variables	Risk ratio	Robust 95% CI
Age		
35-44 years	1.03	(0.95, 1.12)
45-54 years	1.00	(0.92, 1.09)
55-64 years	1.13***	(1.04, 1.22)
65 years and older	1.12**	(1.01, 1.25)
Sex		
Female	1.17***	(1.11, 1.23)
Education		
Less than primary school	1.35***	(1.24, 1.48)
Primary school completed	1.66***	(1.51, 1.82)
Observations	37,012	
Number of countries	30	

*** p<0.01, ** p<0.05, * p<0.1

Notes: Multivariate Poisson regressions estimated with country-level fixed effects and sampling weights rescaled to weigh each country equally.

Appendix 38. Multivariate regression model of dietary fruit and vegetable counseling, restricted to those considered at high risk of diabetes due to BMI and age

Variables	Risk ratio	Robust 95% CI
Age		
35-44 years	1.03	(0.95, 1.12)
45-54 years	1.07	(0.98, 1.16)
55-64 years	1.14***	(1.05, 1.23)
65 years and older	1.23***	(1.11, 1.36)
Sex		
Female	1.09***	(1.04, 1.14)
Education		
Less than primary school	1.08**	(1.01, 1.16)
Primary school completed	1.18***	(1.09, 1.28)
Observations	34,878	
Number of countries	28	

*** p<0.01, ** p<0.05, * p<0.1

Notes: Multivariate Poisson regressions estimated with country-level fixed effects and sampling weights rescaled to weigh each country equally.

Appendix 39. Multivariate regression model of blood glucose measuring, restricted to those considered at high risk of diabetes due to BMI and age

Variables	Risk ratio	Robust 95% CI
Age		
35-44 years	1.22***	(1.12, 1.33)
45-54 years	1.43***	(1.31, 1.56)
55-64 years	1.65***	(1.51, 1.80)
65 years and older	1.83***	(1.66, 2.01)
Sex		
Female	1.13***	(1.09, 1.17)
Education		
Less than primary school	1.37***	(1.28, 1.46)
Primary school completed	1.89***	(1.77, 2.02)
Observations	51,498	
Number of countries	44	

*** p<0.01, ** p<0.05, * p<0.1

Notes: Multivariate Poisson regressions estimated with country-level fixed effects and sampling weights rescaled to weigh each country equally.

Appendix 40. Table of prevalence rates for the population deemed at high risk of diabetes by FINDRISC, classified by World Bank income group.

Country	Proportion high-risk (%)	95% Confidence interval
Low-income countries	6.6	(6.1 - 7.1)
Benin	3.7	(2.8 - 4.9)
Burkina Faso	2.5	(2.1 - 2.9)
Cambodia	4.8	(4.0 - 5.7)
Eritrea	17.0	(14.6 - 19.7)
Liberia	1.8	(1.4 - 2.2)
Rwanda	6.3	(5 - 7.9)
Tanzania	6.7	(5.4 - 8.3)
Togo	9.5	(7.9 - 11.5)
Zanzibar	7.0	(5.4 - 9.1)
Lower-middle income countries	13.6	(13.0 - 14.2)
Bangladesh	7.1	(6.2 - 8.1)
Bhutan	7.6	(6.3 - 9.3)
Eswatini	20.7	(17.7 - 24)
Georgia	32.5	(30.1 – 35.0)
Kenya	8.3	(6.9 – 10.0)
Kyrgyzstan	18.6	(16.5 - 20.9)
Laos	5.6	(4.3 - 7.2)
Lesotho	9.1	(7.8 - 10.5)
Moldova	24.6	(22.6 - 26.7)
Morocco	21.8	(20.5 - 23.3)
Myanmar	6.1	(5.2 - 7.3)
Nepal	6.8	(5.6 - 8.1)
Samoa	38.9	(34.1 - 43.9)
Solomon Islands	11.9	(9.9 - 14.1)
Sri Lanka	8.5	(7.5 - 9.6)
Sudan	9.7	(8.5 – 11.0)
Timor Leste	3.0	(2.3 – 4.0)
Vanuatu	18.9	(16.9 - 21.1)
Vietnam	3.0	(2.4 - 3.8)
Zambia	9.1	(7.9 - 10.5)
Upper-middle income countries	27.0	(25.8 - 28.2)
Algeria	19.7	(18.5 - 21.1)
Azerbaijan	22.1	(20.1 - 24.2)
Belarus	30.3	(28.4 - 32.2)
Botswana	12.8	(11.1 - 14.7)
Chile	29.4	(26.8 - 32.3)
Costa Rica	26.7	(22.4 - 31.5)
Ecuador	25.5	(23.4 - 27.7)
Iran	26.7	(25.7 - 27.7)
Iraq	33.3	(30.8 – 36.0)

Lebanon	20.3	(16.9 - 24.2)
Tokelau	40.0	(31.2 - 49.6)
Tuvalu	37.3	(33.4 - 41.4)

Notes: Estimates of prevalence were calculated with sampling weights readjusted to weigh each country equally

Appendix 41. Proportion of the population deemed high-risk by FINRISC who self-reported having received each diabetes preventive intervention by World Bank income group

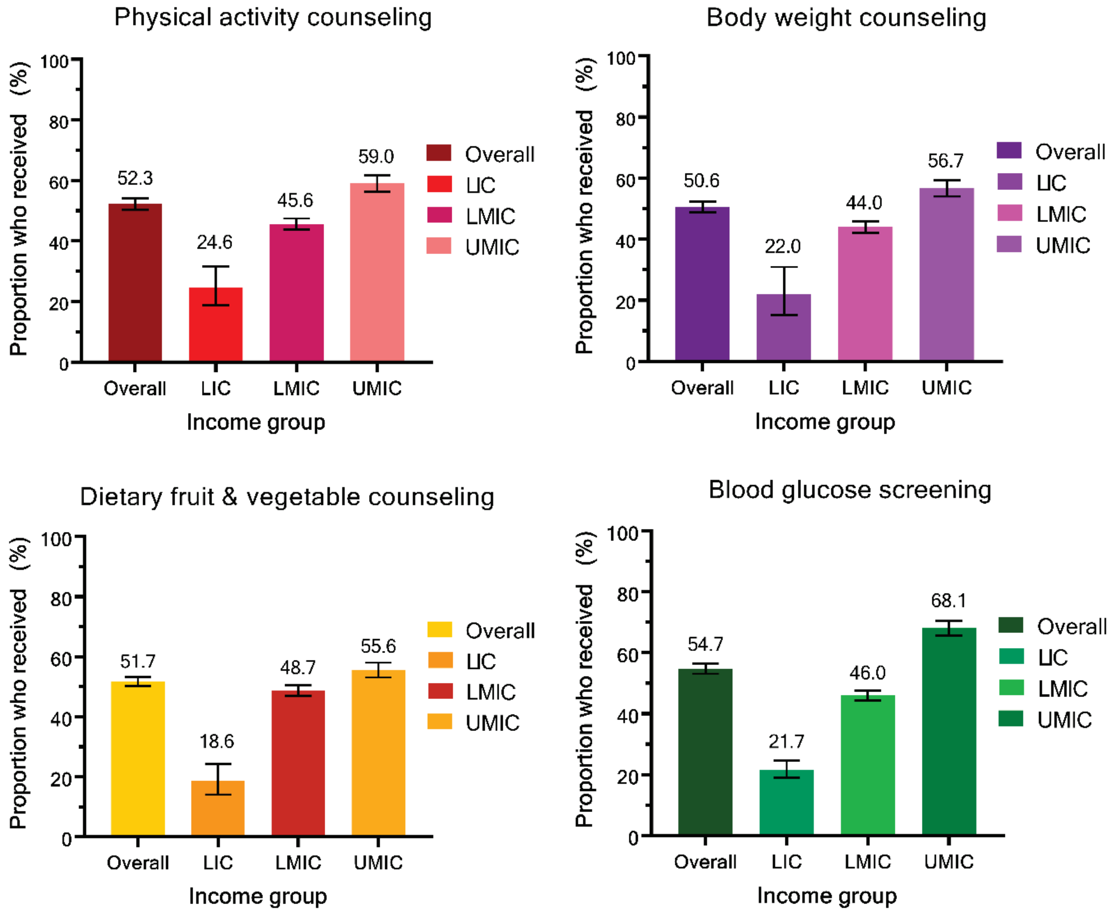

Note: Error bars represent 95% confidence intervals. Sampling weights were adjusted to weigh each country equally

Appendix 42: STROBE checklist

	Item No	Recommendation
**Title and abstract**	1	(*a*) Indicate the study's design with a commonly used term in the title or the abstract **The abstract includes this information.**
		(*b*) Provide in the abstract an informative and balanced summary of what was done and what was found **This information is included in the Abstract.**
**Introduction**		
Background/rationale	2	Explain the scientific background and rationale for the investigation being reported **This information is provided throughout the Introduction.**
Objectives	3	State specific objectives, including any prespecified hypotheses **This information is provided in the final paragraph of the Introduction.**
**Methods**		
Study design	4	Present key elements of study design early in the paper **This information is provided in the Methods, in the subsection on Data sources.**
Setting	5	Describe the setting, locations, and relevant dates, including periods of recruitment, exposure, follow-up, and data collection **This information is provided in the Methods, in the subsection on Data Sources.**
Participants	6	(*a*) Give the eligibility criteria, and the sources and methods of selection of participants. Describe methods of follow-up **This information is provided in the Methods, in the subsection on Data Sources.**
		(*b*) For matched studies, give matching criteria and number of exposed and unexposed **N/A**
Variables	7	Clearly define all outcomes, exposures, predictors, potential confounders, and effect modifiers. Give diagnostic criteria, if applicable **This information is provided in the Methods, subsections on Biomarkers for the diagnosis of prediabetes, Anthropometric measurement of overweight and obesity, Outcomes of interest, and Sociodemographic and healthcare utilization variables**
Data sources/ measurement	8*	For each variable of interest, give sources of data and details of methods of assessment (measurement). Describe comparability of assessment methods if there is more than one group **This information is provided in the Methods, under the subsections on Data Sources, Biomarkers for the diagnosis of prediabetes, Anthropometric measurement of overweight and obesity, Outcomes of interest, and Sociodemographic and healthcare utilization variables**
Bias	9	Describe any efforts to address potential sources of bias **This information is provided in the Methods section, under the subsection on Statistical Analyses.**

Study size	10	Explain how the study size was arrived at<br>**This information is provided in the Methods, under the subsection on Study sample and Definition of population at risk of diabetes**
Quantitative variables	11	Explain how quantitative variables were handled in the analyses. If applicable, describe which groupings were chosen and why<br>**This information is provided in the Methods, under the subsections on Data Sources, Biomarkers for the diagnosis of prediabetes, Anthropometric measurement of overweight and obesity, Outcomes of interest, Sociodemographic and healthcare utilization variables, and Statistical analyses**
Statistical methods	12	(*a*) Describe all statistical methods, including those used to control for confounding<br>**This information is provided in the Methods section, under the subsection on Statistical Analyses.**
		(*b*) Describe any methods used to examine subgroups and interactions<br>**This information is provided in the Methods section, under the subsection on Statistical Analyses.**
		(*c*) Explain how missing data were addressed<br>**This information is provided in the Appendix**
		(*d*) If applicable, explain how loss to follow-up was addressed<br>**N/A**
		(*e*) Describe any sensitivity analyses<br>**This information is provided in the Methods, under the subsection on Sensitivity analyses**
**Results**		
Participants	13*	(a) Report numbers of individuals at each stage of study—eg numbers potentially eligible, examined for eligibility, confirmed eligible, included in the study, completing follow-up, and analysed<br>**A flow diagram is included in the Appendix.**
		(b) Give reasons for non-participation at each stage<br>**A flow diagram is included in the Appendix.**
		(c) Consider use of a flow diagram<br>**A flow diagram is included in the Appendix.**
Descriptive data	14*	(a) Give characteristics of study participants (eg demographic, clinical, social) and information on exposures and potential confounders<br>**This information is provided in the Results Section and in the Appendix**
		(b) Indicate number of participants with missing data for each variable of interest **This information is provided in the Appendix**
		(c) Summarise follow-up time (eg, average and total amount)<br>**N/A**
Outcome data	15*	Report numbers of outcome events or summary measures over time<br>**This information is provided in the Results Section**
Main results	16	(*a*) Give unadjusted estimates and, if applicable, confounder-adjusted estimates and their precision (eg, 95% confidence interval). Make clear which confounders were adjusted for and why they were included<br>**This information is provided in the Results Section and in the Appendix**

		(*b*) Report category boundaries when continuous variables were categorized<br>**These are reported in the Results**
		(*c*) If relevant, consider translating estimates of relative risk into absolute risk for a meaningful time period<br>**N/A**
Other analyses	17	Report other analyses done—eg analyses of subgroups and interactions, and sensitivity analyses<br>**These results are reported in the Appendix.**
**Discussion**		
Key results	18	Summarise key results with reference to study objectives<br>**This information is provided in the Discussion.**
Limitations	19	Discuss limitations of the study, taking into account sources of potential bias or imprecision. Discuss both direction and magnitude of any potential bias<br>**This information is provided in the Discussion.**
Interpretation	20	Give a cautious overall interpretation of results considering objectives, limitations, multiplicity of analyses, results from similar studies, and other relevant evidence<br>**This information is provided in the Discussion.**
Generalisability	21	Discuss the generalisability (external validity) of the study results<br>**This information is provided in the Discussion.**
**Other information**		
Funding	22	Give the source of funding and the role of the funders for the present study and, if applicable, for the original study on which the present article is based<br>**We have provided this information in the section titled "Funding".**

*Give information separately for exposed and unexposed groups.
